# Exploring chromatin hierarchical organization via Markov State Modelling

**DOI:** 10.1371/journal.pcbi.1006686

**Published:** 2018-12-31

**Authors:** Zhen Wah Tan, Enrico Guarnera, Igor N. Berezovsky

**Affiliations:** 1 Bioinformatics Institute (BII), Agency for Science, Technology and Research (A*STAR), Matrix, Singapore; 2 Department of Biological Sciences (DBS), National University of Singapore (NUS), Singapore; CNAG - Centre Nacional d’Anàlisi Genòmica and CRG - Centre de Regulació Genòmica, SPAIN

## Abstract

We propose a new computational method for exploring chromatin structural organization based on Markov State Modelling of Hi-C data represented as an interaction network between genomic loci. A Markov process describes the random walk of a traveling probe in the corresponding energy landscape, mimicking the motion of a biomolecule involved in chromatin function. By studying the metastability of the associated Markov State Model upon annealing, the hierarchical structure of individual chromosomes is observed, and corresponding set of structural partitions is identified at each level of hierarchy. Then, the notion of effective interaction between partitions is derived, delineating the overall topology and architecture of chromosomes. Mapping epigenetic data on the graphs of intra-chromosomal effective interactions helps in understanding how chromosome organization facilitates its function. A sketch of whole-genome interactions obtained from the analysis of 539 partitions from all 23 chromosomes, complemented by distributions of gene expression regulators and epigenetic factors, sheds light on the structure-function relationships in chromatin, delineating chromosomal territories, as well as structural partitions analogous to topologically associating domains and active / passive epigenomic compartments. In addition to the overall genome architecture shown by effective interactions, the affinity between partitions of different chromosomes was analyzed as an indicator of the degree of association between partitions in functionally relevant genomic interactions. The overall static picture of whole-genome interactions obtained with the method presented in this work provides a foundation for chromatin structural reconstruction, for the modelling of chromatin dynamics, and for exploring the regulation of genome function. The algorithms used in this study are implemented in a freely available Python package ChromaWalker (https://bitbucket.org/ZhenWahTan/chromawalker).

## Author summary

A new era in chromatin research started with the availability of Hi-C data and new experimental techniques driving improvements in data resolution enable us to achieve a deeper understanding of the chromatin structure and function, while calling, at the same time, for the development of more advanced analytical methods. Though instrumental in the analysis of Hi-C data, both model-driven polymer models and data-driven statistical approaches are always based on several assumptions and require tweaking parameters. We interpret the Hi-C frequencies of chromatin interactions in terms of pairwise contact energies, obtaining corresponding energy landscape that represents the structure and interactions in chromatin. The ruggedness of this landscape is explored by the random walk of a travelling probe, which is formalized in the framework of a Markov State Model. The multilevel energy landscape induces metastability in the Markov process, revealing the hierarchy of chromatin structural organization. Structural partitions determined by the basins in the energy landscape are, thus, naturally obtained at different levels of hierarchy without any preliminary assumptions. Effective interactions between partitions are evaluated, providing a blueprint of the whole-genome organization and functional interactions, which is further substantiated by mapping of information on gene expression regulators and different epigenetic factors. The notion of affinity between partitions complements the picture by reflecting the degrees of association between partitions, calling for the modelling of chromatin dynamics and exploring its functional modulation.

## Introduction

The packing of two meters of DNA in the few-micrometer nucleus results in a structure that performs multiple roles, from forming a structural scaffold of chromatin to facilitating active expression and silencing of genetic material [1, 2]. The beginning of interest in the biophysical characterization of chromatin dates to about 50 years ago, spanning from experimental measurements of DNA persistence length [3–5] and thermal stability [4] to pulling individual DNA-protein (DNP) fibrils by convection flows in solution [6], exploring fibril morphology and stability under different media conditions [7], and exposure to ionizing radiation [5].

Before the chromosome conformation capture (3C) [8] era, the classical view of chromatin organization included several successive levels of packing with archetypal structural patterns, ranging from the compaction of nucleosome-bound 10nm fibers [9] with a roughly 200 base-pair periodicity, to the transient 30nm solenoid (hard to detect *in vivo*) presumably working in the regulation of gene expression [10, 11], then to the 30-100kbp loops/domains that are apparently instrumental in shaping large-scale chromatin organization and gene expression [1, 12–19]. With the development of the chromosome conformation capture (3C) protocol [8], it has become possible to study chromatin interactions between distant genomic loci. In less than a decade, the original 3C protocol evolved from the analysis of selected pairs of genomic loci to the detection of pairwise interactions between loci and the rest of the genome using chromosome conformation capture on-chip (one-to-all, 4C, [20]), carbon copy (many-to-many, 5C, [21]), and high-throughput 3C (all-to-all, Hi-C, [22]). Finally, improvement of the signal-noise ratio was achieved by performing DNA proximity ligation before nuclear lysis, implemented in *in-situ* Hi-C [23].

Computational approaches for the analysis of chromatin interaction data developed in recent years can be classified as model-driven or data-driven [24]. Generally, the goal of model-driven studies is to validate physical polymer simulations using Hi-C data. Among them are models of chromatin as a crumpled (fractal) globule [22, 25–27], scenarios of loop formation [28, 29], analyses of the role of epigenetic factors in driving the chromatin organization [30–34], to name a few. In data-driven studies, on the other hand, experimental Hi-C interaction maps are used for extracting information on statistically significant chromatin interactions, for defining topologically associating domains (TADs) and A/B compartments [35, 36], and for the 3D reconstruction of chromatin. Several algorithms have been introduced to study the hierarchical organization of chromatin and its correlation with the distribution of various epigenetic features [37–40], including graph-based approaches for exploring sparse networks of Hi-C interaction peaks, as well as ChIA-PET and HiChIP interaction pairs [41–43]. A recent work by Pancaldi *et al*. defined chromatin assortativity as a metric for the analysis of correlation between distributions of epigenetic marks and chromatin structure [44]. To date, many methods developed for domain detection [23, 45–47] essentially adopt an image segmentation approach aimed at identifying domain regions as a function of short-range interactions along the chromosome, and domain boundary positions are often highly sensitive to the choice of heuristic tuning parameters [48]. Recent network-based methods incorporate effects of long-range interactions in characterizing structural organization [37, 39, 49] and observe spatial couplings at multiple scales associated with the regulation of gene expression [50]. Spatial reconstructions of chromatin using Hi-C interaction data yield consensus structures [51, 52] or ensembles of possible chromosomal conformations [53, 54], providing an overall picture of chromatin organization [55].

In this work, we propose a new approach for extracting robust genomic partitions from Hi-C data, seeking to capture the footprints of chromatin structure and organization by considering the entire interaction landscape of this complex system. Specifically, our objectives here are to identify and study structural features of chromatin from Hi-C interaction data and to find a connection between these features and data on epigenetic regulation. Introducing a Markov State Model (MSM) with minimal assumptions and parameters on the chromatin interaction network, we aim to identify structural partitions and interactions between them. By analogy with a biomolecule moving and interacting in condensed chromatin, the MSM allows one to explore chromatin structure using a “probe” randomly walking in the contact energy landscape derived from Hi-C data. Given the multiscale nature of the data-derived contact energy landscape and the metastability of the corresponding MSM, we can identify regions of dense intra- and inter-chromosomal interactions, linkers between these regions, as well as the overall topology of individual chromosomes and the complex structures that chromosomes form by interacting with each other. We found that multiple levels of hierarchy exist in the structure of each chromosome with a layer-by-layer splitting of partitions into subunits with distinct structural and epigenetic features, and presumably, distinct roles. The notion of effective interaction between partitions is introduced and shown to be instrumental in uncovering the hierarchical organization, as well as functional dynamics and epigenetic modulation, of individual chromosomes. Looking at the whole-genome picture, the matrix of effective interactions delineates how chromosomal partitions form a major cluster—with several chromosomes linked by significant inter-chromosomal interactions—as a structural scaffold for genome architecture. The notion of affinity between partitions complements the picture of effective interactions by evaluating the degree of association between partitions, which may contribute to the formation of topologically associated domains, transcription factories and other functional elements, thereby organizing the regulation of genome expression.

## Results

In this paper, we propose a novel computational method for exploiting Hi-C data in the study of chromatin organization. Since Hi-C reads represent interactions between pairs of loci, it is natural to consider Hi-C data as an undirected network of contacts between genomic loci, which, as a highly complex system at a resolution of 50kbp, contains tens of thousands of nodes at the whole-genome level. In the following, we first provide the motivations for adopting a Markov State Model approach for the analysis of Hi-C data, then introduce a toy model of a chromosome that serves to elucidate the most important notions associated with the method. Finally, a specific description of the major steps in the proposed Markov State Model approach is presented for the case of a single chromosome (human chromosome 17), followed by a genome-wide analysis.

### Markov State Modelling: A toy chromosome as an example

A common strategy to study complex network data structures is to combine them with a discrete state Markov process, commonly called a Markov State Model (MSM), with the goal of characterizing hidden network properties [56–58]. MSMs enable one to systematically explore network structure via random walks, where traveling probes form virtual trajectories through the whole network by connecting pairs of nodes. It has been shown that studying the spectral and metastability properties of the network-associated MSM allows one to obtain a reduced description of the underlying complex data.

**Fig 1:**
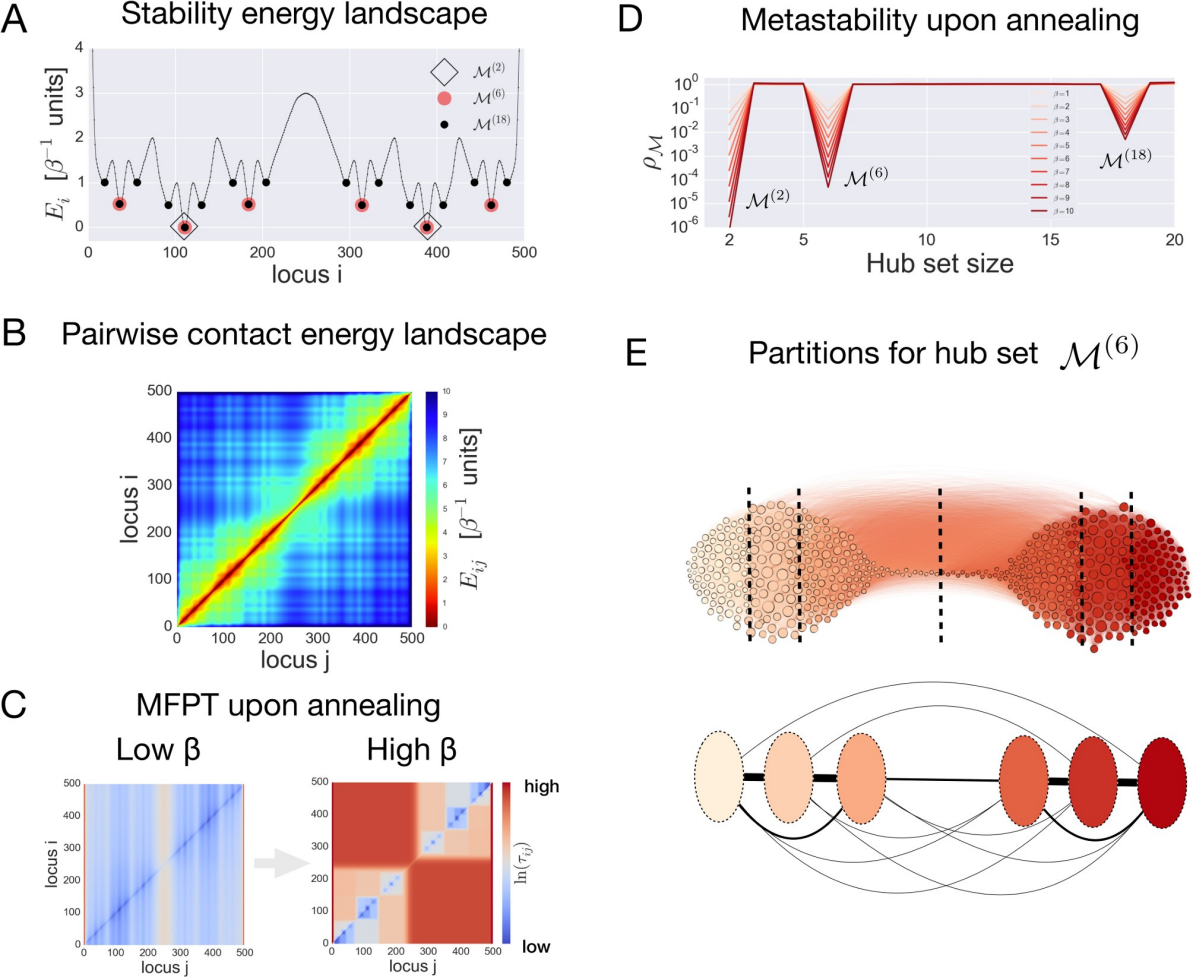
The toy model of a chromosome with a hierarchical energy landscape. **(**A) The energy landscape with three levels of hierarchy considered here. (B) Pairwise contact energy landscape for the toy model of a chromosome of length 500. (C) Illustration of the effect of annealing on the mean-first passage time (MFPT) between the states. (D) Optimization of the metastability index results in profiles, revealing three levels of hierarchy corresponding to three hub sets $M^{\left(2\right)},M^{\left(6\right)}$, and $M^{\left(18\right)}$, respectively. (E, top) A network representation of the toy chromosome interactions with nodes colored according to the partitions associated with the hub set $M^{\left(6\right)}$; (E, bottom) reduced network constructed from the partitions associated with the hub set $M^{\left(6\right)}$, with links depicting effective interactions between partitions calculated via Eq 11 in Materials and methods.

In order to illustrate how MSMs can be used for studying Hi-C data, we introduce here a toy model of a chromosome. Let us consider a linear system characterized by a discrete set of loci *S* = {1,…,*N*}, with *N* = 500. We assume that the number of loci *N* determines the maximal resolution of this relatively large system. Each locus of the system is associated with an energy *E*_*i*_, which is linked to the intrinsic stability of the locus *i* at the given resolution. For the sake of argument, we assume the intrinsic stabilities *E*_*i*_ to follow a hierarchically shaped energy profile (Fig 1A). The energy profile considered here contains 18 wells separated by barriers ranging from 0.5 to 2 energy units. On the first level of hierarchy, there are two basins separated by a barrier of 2 energy units (black diamonds in Fig 1A), each divided into three sub-basins (indicated as red circles in Fig 1A), which in turn are split into three basins on the third level of hierarchy (black circles in Fig 1A).

A traveling probe in such an energy profile is assumed to make two types of moves: sliding between adjacent loci and hopping between non-adjacent ones. We do not make any assumption about the three-dimensional structure of the system and assume a power law contact probability between non-adjacent loci, namely (*d*_0_/*d*_*ij*_)^*α*^, where *d*_0_ and *d*_*ij*_ are the distance between adjacent and any non-adjacent loci respectively, which is equivalent to the genomic distance between loci *i* and *j* and such that *d*_*ij*_ = *d*_0_ for adjacent loci. Thus, for each pair of loci *i* and *j*, we define the contact energy landscape *E*_*ij*_ = (*E*_*i*_ + *E*_*j*_)/2 − *α*ln *d*_0_/*d*_*ij*_ with *α* = 1.5. Assumptions on the power law dependence and the value of exponent *α* are made on the basis of empirical observations on Hi-C data and polymer models of chromosomes [22]. The contact energy landscape is represented in Fig 1B.

To construct the MSM describing the motion of a probe, we define the corresponding Markov generator *L* for transitions between loci *i* → *j* by the Laplacian $L_{ij}=e^{-\beta (E_{j}-E_{i})/2}e^{\beta \ln{(d_{0}/d_{ij})}^{\alpha }}$ and *L*_*ii*_ = −∑_*j*≠*i*_*L*_*ij*_, with transition matrix *p*_*ij*_ = *L*_*ij*_/∑_*j*≠*i*_*L*_*ij*_,*p*_*ii*_ = 0(∑_*j*_*p*_*ij*_ = 1), flux *π*_*ij*_ = *L*_*ij*_*μ*_*j*_, where steady state probabilities are given by $\mu_{i}=e^{-\beta E_{i}}/\sum_{j}e^{-\beta E_{j}}$, and *β* is an inverse temperature parameter. A network of nodes (loci) and edges (contacts) is obtained from the matrix of fluxes *π*_*ij*_, which represents the symmetric probability of contact between a pair of loci. With the set of rules given by the above Markov generator, a probe will tend to explore regions of the network in the neighborhood of the loci that are more stable, *i*.*e*., within an energy well, and will rarely connect loci in different energy wells. This property relates to the “metastability” of the corresponding Markov process. Specifically, in a metastable MSM only a few nodes function as “hubs” of the network, which means that the probe tends to spend most of the time in the neighborhood of these hub nodes, instead of anywhere else. In other words, a probe departing from a generic node in the network is likely to hit the closest hub node in the set hub-nodes M. Additionally, the probability for a probe departing from a hub-node in M to return to itself is larger than that for the probe to reach another hub-node in M. As a result, nodes in the neighborhood of hubs tend to cluster together in a modular manner. This is a condition that allows one to find a reduced size MSM that approximates the original Markov process associated with the initial network. One can quantify how well the probe motion satisfies this condition by defining a metastability index $\rho_{M}$. A metastability index is the ratio of two probabilities (see Eq 6 in Methods for a precise definition): the probability *P*_*out*_ for a probe to connect two different hubs in the set M (as small as possible) over the probability *P*_*in*_ for a walker to hit any hub in the set M irrespective of the starting point (as large as possible) [59]. In a metastable MSM the metastability index is expected to be a small number ($\rho_{M}=P_{out}/P_{in}<1$) characteristic of the hub set M.

To understand how metastability works, it is instructive to consider the large changes in kinetic properties of the MSM upon increase of the inverse temperature parameter *β* (annealing condition). These changes are clearly illustrated by the mean first passage time MFPT *τ*_*ij*_, which is the average time (number of steps) a probe takes to connect the pair of states *i* and *j* (where states represent loci of the toy chromosome, see Eq 4 in Methods). Fig 1C shows the MFPT matrices in the case of low *β* = 1 and high *β* = 10, respectively. A clear separation of time scales emerges upon increasing 𝛽, as reflected in the partitioning of the MFPT matrices. The nested squares emerging in the MFPT matrix (Fig 1C) at high *β* identify pairs of states/loci (*i*,*j*) with comparable values of the MFPT *τ*_*ij*_, which is a result of the hierarchically shaped energy profile. As *β* is increased, the emerging separation of time scales in the MFPT matrix is the result of the dominant barrier that separates a given pair of loci in the energy landscape (see Fig 1A): each of the separated regions contains one or more hubs that cause probes to stay within its vicinity. As a result, the effect of high *β* on the MFPTs of the MSM elucidates how dominant interactions in the system can be captured using just a subset of loci, the hub set M.

To quantitatively identify the hub set, an optimization procedure is performed in order to find the sets M that minimize the metastability index $\rho_{M}$ (see details in Materials and Methods) as a function of increasing *β*. Fig 1D shows the optimized profile of the index $\rho_{M}$ as a function of the hub set sizes, and at different values of *β*. All the profiles of $\rho_{M}$ clearly show three minima corresponding to the hub sets $M^{\left(2\right)},\;M^{\left(6\right)}$, and $M^{\left(18\right)}$ (of sizes 2, 6 and 18, respectively), which correctly identify locations of the energy wells in the hierarchically shaped energy landscape in Fig 1A. The hub sets obtained by optimizing the index $\rho_{M}$ are suitable as cores of partitions, which characterize the coarse-grained state space of an approximated MSM. Fig 1E (top) depicts the toy network associated with the contact energy landscape shown in Fig 1B. Nodes are colored according to the partitions constructed around nodes in the hub set $M^{\left(6\right)}$. A reduced network corresponding to the hub set $M^{\left(6\right)}$ is also shown in Fig 1E (bottom). The nodes in this network are defined as soft partitions of the initial set of loci *S*, whereas the links characterize the “effective interactions” between nodes with values *F*_*ab*_ = ∑_*i*∈*S*_*q*_*a*_(*i*)*π*_*ib*_ (see Eq 11 in Materials and Methods). The quantity *q*_*a*_(*i*) is a committor probability, which is the probability for a probe departing from a locus *i* to hit the locus a∈M before any other locus in the hub set M (see Eq 8 in Materials and Methods).

Using the intuition acquired with the help of this toy model, we describe in the following section how a MSM can be constructed from the Hi-C dataset of a single chromosome and how metastability analysis can be performed in order to infer chromosomal architecture and effective interactions between partitions.

### Hi-C data and Markov State Modelling: From a single chromosome to the whole genome

**Fig 2:**
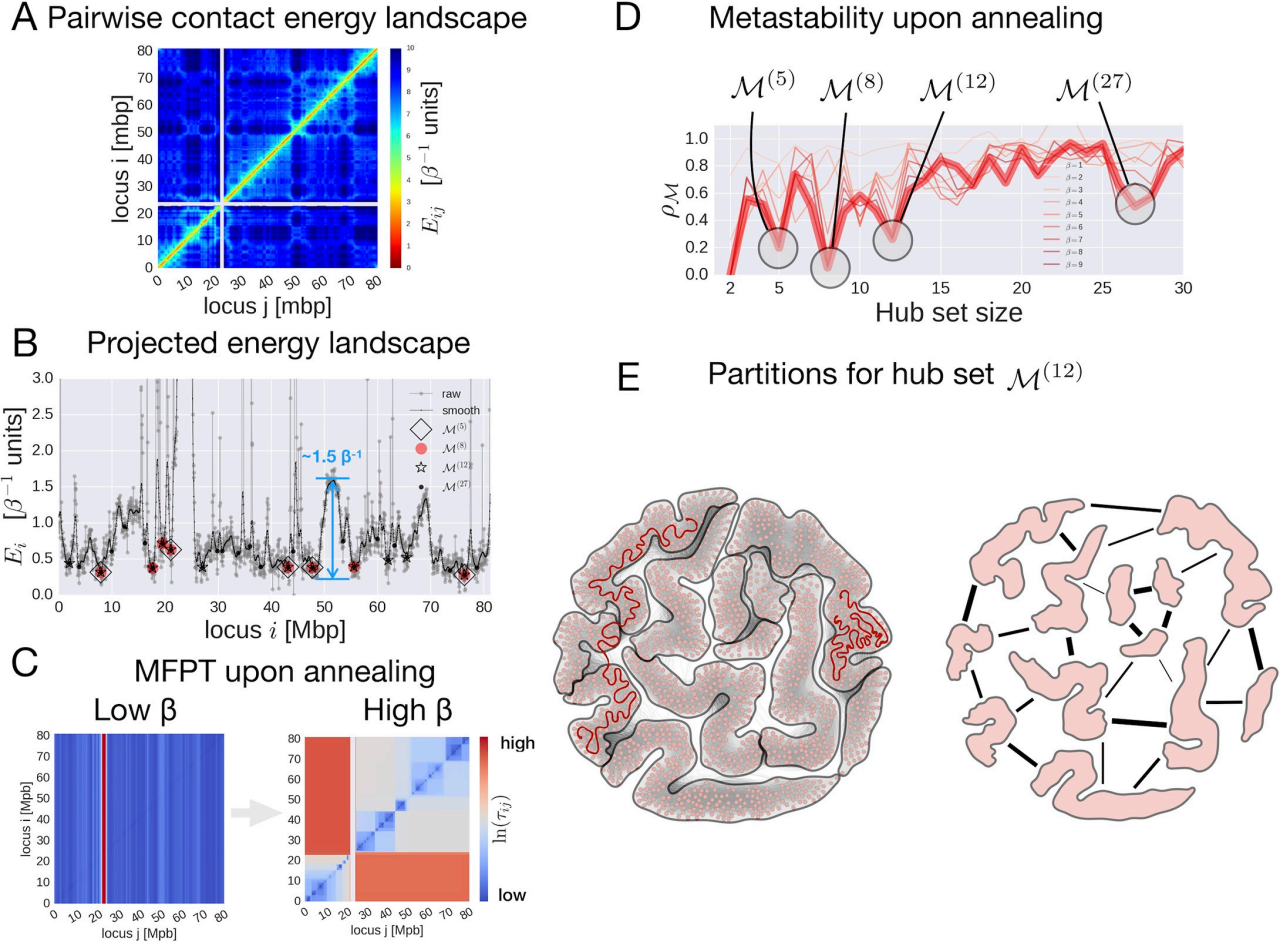
Analysis of human chromosome 17 using a MSM-based computational framework. **(**A) Pairwise contact energy landscape of human chromosome 17. (B) 1D projection of the chromosome 17 energy landscape. (C) Effect of annealing on the mean-first passage time (MFPT) between loci. (D) Optimization of the metastability index $\rho_{M}$ under different annealing conditions (*β* = 1 to 9). (E) Partitioning of chromosome 17 determined by the hub set $M^{\left(12\right)}$ (left, visualization obtained by the Fruchterman-Reingold visualization algorithm implemented in Gephi [60]) and schematic illustration of the effective interactions between 12 observed partitions (right).

We now consider the random walk through the interaction network of a single chromosome, using the example of Hi-C data on human chromosome 17 in the human B lymphoblastoid cell line GM12878 at 50kbp resolution [23] and describing it via a Markov process. To do that, we start from the number of times *f*_*ij*_ a pair of genomic loci *i* and *j* is found in a contact. After applying a Gaussian smoothing filter on the raw data (see Hi-C data preprocessing in Materials and Methods), a pairwise contact energy *E*_*ij*_ = −ln*f*_*ij*_ is defined for each pair of genomic loci. With this interpretation, the larger the contact frequency the more stable (lower contact energy) pair of genomic loci is involved. The representation of this two-dimensional contact energy landscape is shown in Fig 2A.

A probe moving in such a landscape is expected to spend most of the time in pairs characterized with low contact energy and rarely connecting across high contact energy pairs. In the toy model presented in the previous section, a pairwise contact energy landscape (Fig 1B) was constructed from the one-dimensional energy landscape (Fig 1A). Here, we use a reverse logic and consider the one-dimensional projection (Fig 2B) of the two-dimensional contact energy landscape (Fig 2A). To do that we define the contact energy of a genomic locus *i* as *E*_*i*_ = −ln*f*_*i*_, where *f*_*i*_ = ∑_*j*_*f*_*ij*_ is the total number of times a genomic locus *i* is found in any contact, hence loci involved in more contacts are more stable as they exhibit lower contact energy. Fig 2B shows the 1D projection of the pairwise contact energy landscape (for both raw and Gaussian-smoothed data), which presents multiple features—minima, maxima, and barriers—characterizing the architecture of the chromosome.

Here, we briefly describe the metastability analysis applied to chromosome 17 (steps 1–4) and consider whole-genome interactions (step 5) using a coarse-grained approximation.

**Step 1.** In order to explore chromosomal architecture, the MSM describing the motion of a probe in the contact energy landscape *E*_*ij*_ is implemented by introducing the Maxwell-Boltzmann probability $\pi_{ij}(\beta )=e^{-\beta E_{ij}}/Z(\beta )$, where $Z(\beta )=\sum_{\left(i,j\right)}e^{-\beta E_{ij}}$ is the partition function, *β* is the inverse temperature parameter, and *π*_*ij*_ is the symmetric flux of probes between pairs of loci. Using a 50kbp resolution for the Hi-C dataset, a total of *N* = 1625 genomic loci comprise the state space *S* of the MSM for chromosome 17. The transition matrix associated with the MSM is defined as *p*_*ij*_ = *π*_*ij*_/*μ*_*i*_, where *μ*_*i*_(*β*) = ∑_*j*_*π*_*ij*_(*β*) is the Boltzmann weighted probability (steady state probability distribution) of observing locus *i* involved in any contact. The effect of annealing (increasing the inverse temperature *β*) on the kinetics of a random walker is clearly reflected in the MFPT matrices (Fig 2C), obtained at low and high *β*, respectively. While at low *β* (*β* = 1) MFPTs show no partitioning, a separation of time scales becomes evident at high *β* (*β* = 9). Indeed, the 1D projection of the pairwise pseudo-energy landscape (Fig 2B) shows that, apart from the centromere that naturally separates the two chromosome arms, the highest barrier in the 1D projection is about 1.5 in *β*^−1^ units (see Fig 2B). Therefore, partitioning of MFPTs scales is observed only for significantly higher values of *β*.

**Step 2.** Optimization of the metastability index $\rho_{M}$ (see details in Materials and Methods) over the hub set M of different sizes was performed as a function of the inverse temperature parameter *β*, revealing the levels of structural hierarchy of chromosome 17. The $\rho_{M}$ profiles upon increasing *β* (Fig 2D) converge towards five minima, which correspond to the hub sets $M^{\left(2\right)},M^{\left(5\right)},M^{\left(8\right)},M^{\left(12\right)},$ and $M^{\left(27\right)}$, of sizes 2, 5, 8, 12, and 27, respectively. The $M^{\left(2\right)}$ hub set is not considered as it trivially identifies the chromosome arms separated by the centromere. It should be noted that the locations of the obtained hub sets correspond to the locations of the multiple wells present in the projected contact energy landscape, as shown in Fig 2B.

**Step 3.** Given the hub sets obtained at different levels of structural hierarchy, one can identify chromosomal partitions, namely regions of the chromosome compacted around corresponding hubs and, at the same time, separated from one another. Soft partitions are defined around corresponding hubs using the committor probability *q*_*a*_(*i*) [59], which in this case is interpreted as the probability for a locus ***i*** to belong to the partition defined by the hub a∈M. To identify physical partitions of the chromosome in relation to other chromosomes, a coarse-grained description is adopted here by considering hard partitions. In this case, a step function *θ*_*A*_(*i*) characterizes whether a locus ***i*** belongs to a partition *A*, specifically *θ*_*A*_(*i*) = 1 if *i* ∈ *A*, *θ*_*A*_(*i*) = 0 otherwise, and ∑_*A*_*θ*_*A*_(*i*) = 1 for any locus ***i*** (see Eq 9 in Materials and Methods). Fig 2E illustrates the partitioning of the network for human chromosome 17 that is obtained from the hub set $M^{\left(12\right)}$.

**Step 4.** To complete the description of chromosome structure, one needs also to characterize the strength of interactions between the partitions obtained at different levels of hierarchy. As in the example illustrated in the toy model, we consider the effective interaction between two soft partitions located around the hub loci *a* and *b* of a chromosome as the mean contact energy acting between them, which corresponds to the weighted flux connecting loci *a* and *b* via the committor probability *q*_*a*_(*i*), namely *F*_*ab*_ = ∑_*i*∈*c*_*q*_*a*_(*i*)*π*_*ib*_ (see Eq 11 in Methods).

**Step 5.** In the context of whole-genome interactions, a coarse-grained description is adopted (see Step 3) for estimating the mean contact energy between pairs of partitions in the 23 chromosomes: *F*_*AB*_ = ∑_*i*∈*g*_*θ*_*A*_(*i*)∑_*j*∈*g*_*π*_*ij*_*θ*_*B*_(*j*), where *θ*_*A*_(*i*) and *θ*_*B*_(*i*) are step functions and *π*_*ij*_ is the flux of probes between corresponding loci (see Materials and Methods for details).

### Effective interaction between eu- and heterochromatic partitions in the structural hierarchy of chromosome 17

**Fig 3:**
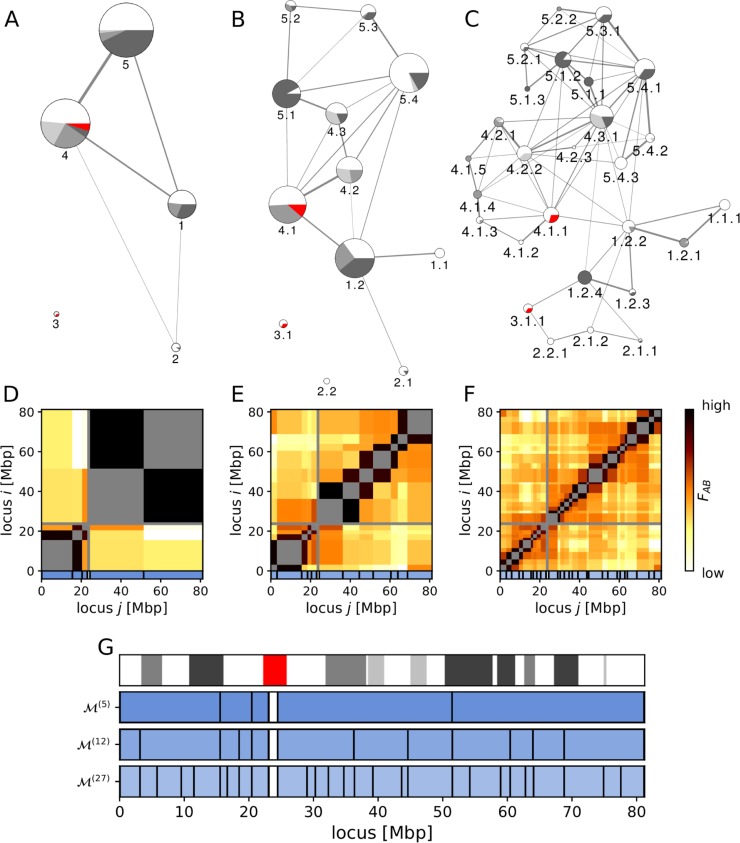
Architecture of the chromosome 17 at three levels of structural hierarchy. (A-C) Graph representation of three levels of structural hierarchy. The nodes represent partitions, the node sizes scale with partition sizes, and pie charts in nodes indicate the euchromatin/heterochromatin composition of corresponding partitions obtained from the Giemsa staining (with red denoting the centromere). The color gradient (25, 50, 75, and 100%) corresponds to heterochromatic bands with corresponding degrees of compactness. The width of edges indicates the effective interaction strength, which is obtained from the effective interaction matrices at each level of hierarchy (D-F). Partitions are labelled at each level to reflect the strict hierarchy: partition 1 contains sub-partitions 1.1 and 1.2, partition 1.2 contains sub-partitions 1.2.1, 1.2.2, 1.2.3, and 1.2.4, and so on. Weak interaction edges are omitted for clarity (see Materials and Methods for details). (G) Band representation of partitioning at the three levels of hierarchy ($M^{\left(5\right)},\;M^{\left(12\right)},M^{\left(27\right)}$). Partition boundaries observed at the lowest level persist in higher levels of hierarchy, indicating the presence of a strict hierarchy in the chromosome structural organization. To guide the eye on how the different types of chromatin packing are distributed across partitions, Giemsa staining bands are also shown on top of the partition diagrams for all levels of hierarchy.

Fig 3A–3C show the partitioning of chromosome 17 at three levels of hierarchy with corresponding effective interaction matrices (Fig 3D–3F), and the band representation of partitions at all three levels ($M^{\left(5\right)},\;M^{\left(12\right)},M^{\left(27\right)}$; Fig 3G). The major partition boundaries that emerge at the first level of hierarchy persist through the other levels (Fig 3G; similar for all chromosomes, see S1 Fig) and show a qualitative agreement with the borders of euchromatic and heterochromatic bands obtained from Giemsa staining (Fig 3G and S1 Fig). Unfortunately, as Giemsa staining is a very basic and crude cytological method for identifying densely-packed (heterochromatic, dark stain) and low-density (euchromatic, light stain) genomic regions, it is not possible to perform an accurate quantitative analysis on staining bands [61, 62].

At the lowest level of hierarchy (Fig 3A), we observed the bulk topology of the chromosome where two chromosomal arms are brought together via strong interactions between partitions 1, 4, and 5. Most partitions at the lowest levels of hierarchy are found to contain both euchromatic and heterochromatic bands, and they have highly distinct structural and/or functional characteristics. At the second level of hierarchy (Fig 3B), partitions 1.2, 4.1, 4.2, 5.1, and 5.4 form several non-adjacent contacts, working as hubs responsible for most of the network structure. The third level of hierarchy (Fig 3C) yields further details of chromosomal architecture: the p-arm is loosely connected and is weakly centered on 1.2.2 and 1.2.4, while the q-arm is densely connected by multiple hubs (4.1.1, 4.2.2, 4.3.1, 5.1.2, and 5.4.1). At this level many partitions are homogeneous, either eu- or heterochromatic, interacting more strongly with partitions with similar packing densities, resembling the phenomenology of the so-called A/B (active/inactive) chromatin compartments [22]. For instance, partition 1.2 is split into mostly euchromatic (1.2.2, 1.2.3) and heterochromatic (1.2.1, 1.2.4) partitions, while partition 4.1 is split into predominantly euchromatic (4.1.1, 4.1.2, 4.1.3) and heterochromatic (4.1.4, 4.1.5) ones. Partition 4.1.1 is the largest among these, forming significant interactions with the p-arm through partition 1.2.2. Another noticeable interaction between chromosomal arms occurs via the partition 4.3.1, which links heterochromatic partitions 5.1.1–2 and 1.2.4. Interestingly, the mostly euchromatic partition 1.2.2 is responsible for many non-adjacent contacts with the q-arm, whereas heterochromatic 1.2.4 forms non-adjacent contacts only with 4.3.1 and 3.1.1. With these observations, one may conclude that heterochromatic partition 1.2.4 acts as a structural foundation that link the mostly euchromatic partitions 1.2.2, 1.2.3, 2.1.1, 2.1.2, and 3.1.1.

### Structure-function relations between partitions of chromosome 17: Partition sizes and distributions of CTCF and cohesin

**Fig 4:**
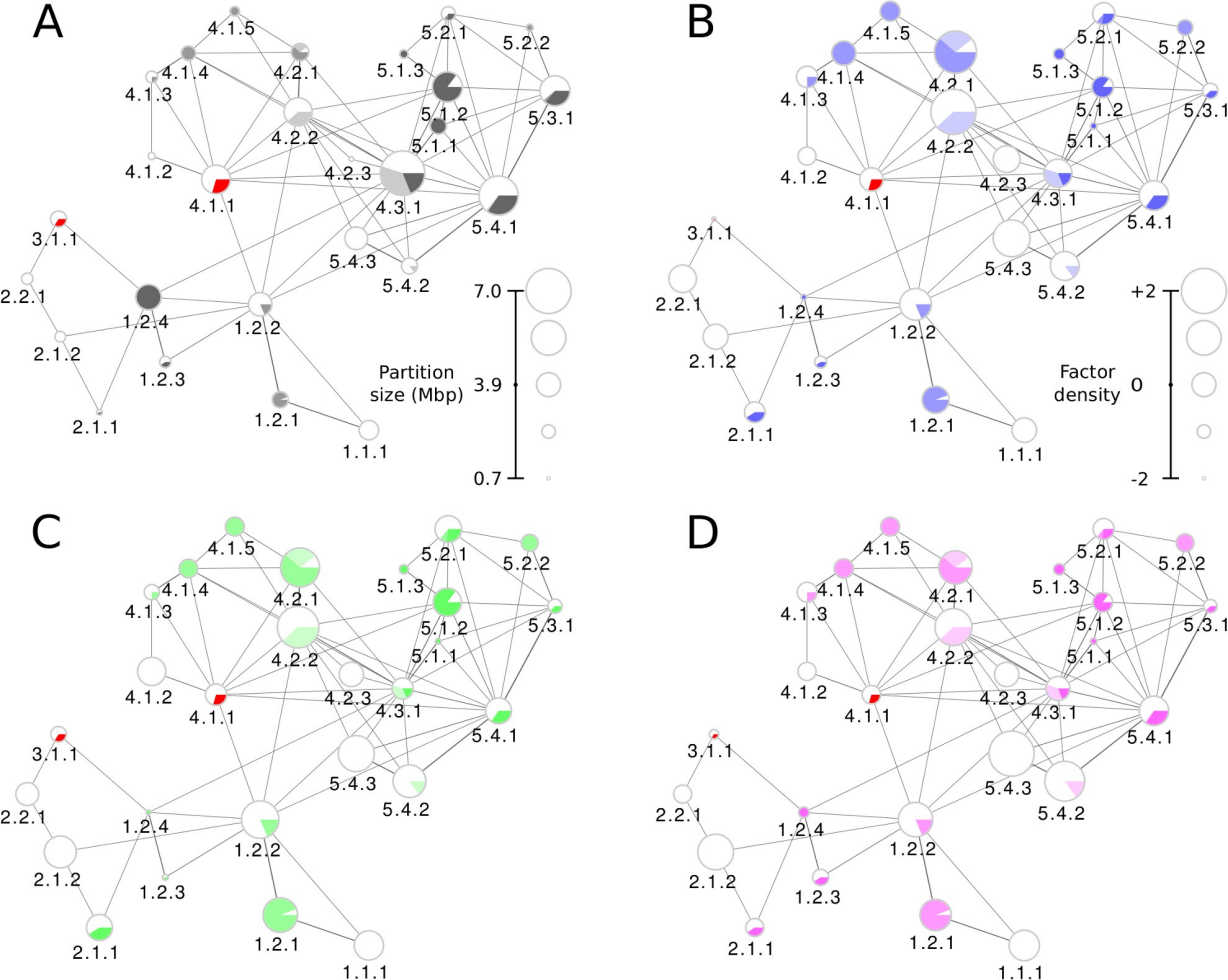
Distribution of epigenetic factors in partitions of chromosome 17 at the third level of structural hierarchy. Partitions are represented as pie-charted nodes depicting the presence of eu- and heterochromatin (on the basis of Giemsa-staining) within the partition. The node sizes are set according to the partition size or Z-scored density of the factor in the corresponding partitions (see scales in corresponding panels). Edge widths correspond to effective interaction strengths, while node sizes in each panel represent the (A) partition size, and factors’ Z scores for (B) CTCF, (C) H3K9ac, and (D) DNase-Seq epigenetic factors. Visual legends show how the values corresponding to each partition scale with the node size.

To investigate how the hierarchical organization of chromosomes facilitates their function, we first analyzed the average density of various epigenetic factors in partitions (Fig 4 and S3 Fig), using chromosome 17 as an illustration for this analysis and operating at the third level of structural hierarchy. Fig 4A, in which node sizes depict partition sizes, shows that heterochromatic partitions 5.1.1 and 5.1.2 apparently form a structural foundation of chromosome 17 architecture, linking the p- and q-arms through the large mixed partition 4.3.1 and the heterochromatic partition 1.2.4. Next, we consider two transcription factors commonly associated with chromatin structure studies, namely CTCF (transcriptional repressor, Fig 4B) and RAD21 (cohesin, S3H Fig). The CTCF graph (Fig 4B) shows that the heterochromatic partitions (1.2.4, 5.1.1, 5.1.3, and 5.2.2) and the pericentromeric partition 3.1.1 have the lowest CTCF levels, while the highest CTCF levels were found on 4.2.2, 4.2.1, and 5.4.3. The euchromatic or mostly euchromatic partitions 1.1.1, 1.2.2, 2.1.2, 2.2.1, 4.2.3, 5.4.1, and 5.4.2 show average levels of CTCF in the overall eight-fold variation in the density of this transcription factor across partitions. Among the hub partitions, namely those that form extensive non-adjacent contacts, only 4.2.2 shows high CTCF levels. Unlike CTCF, RAD21 (a component of cohesin) exhibits only a two-fold variation in densities across partitions at this level of hierarchy. The correlation between CTCF and cohesin binding sites has been noted previously [63, 64], and indeed the distribution of RAD21 (S3H Fig) chiefly follows the same general trends as that of CTCF. The strongest among the few exceptions are the increased density of RAD21 in 4.2.3 and decreased density in 5.4.3.

### Abundance and potential functional role of histone modifications in partitions of chromosome 17

Turning to histone modifications, we note that H3K9ac (Fig 4C) and H3K9me3 (S3A Fig) are associated with activation and silencing of transcription in corresponding promoter regions and, therefore, are expected to show opposite density trends. Indeed, densely packed heterochromatic partitions (1.2.4, 5.1.1, 5.1.3) and pericentromeric 3.1.1 show very low levels of the activating H3K9ac histone modification, while the silencing H3K9me3 modification shows increased density in these partitions (highest in the case of 3.1.1). At the same time, euchromatic and mostly euchromatic partitions 1.1.1, 1.2.2, 2.1.2, 4.1.2, 4.2.2, 5.4.2, and 5.4.3 show an increased density of both epigenetic factors, with some slight variations. The opposing trends are observed in heterochromatic partitions for the activating H3K27ac (decreased density, S3B Fig) and inhibiting H3K27me3 (increased density, S3C Fig) modifications, with the most pronounced effects being on 1.2.1, 4.1.4, 4.1.5, 5.1.1, and 5.1.3. Distributions of the H3K4me1 (S3D Fig) and H3K4me3 (S3E Fig) modifications—both activators—show higher densities in most euchromatic partitions, and in few heterochromatic ones—1.2.1, 4.2.1, and 5.1.2. Interestingly, the heterochromatic partitions 1.2.1, 4.2.1, and 5.1.2 are enriched in all activating histone modifications considered here (H3K4me1, H3K4me3, H3K9ac, H3K27ac), and, at the same time, are depleted in the inhibiting modifications H3K9me3, H3K27me3. These trends suggest that the above partitions may contain facultative heterochromatin that switches between active and repressed states.

### DNA accessibility and density of RNA polymerases II and III in partitions of chromosome 17

Overall, the DNA accessibility graph, indicating the DNase-Seq signal (Fig 4D), shows that most of the euchromatic partitions (1.1.1, 1.2.2, 2.1.2, 4.2.2, 5.4.2, and 5.4.3) are rather open and accessible for contacts or interactions. Increased accessibility observed for partitions 4.2.1 and 1.2.1 is consistent with the conclusion that these partitions may contain facultative heterochromatin, which was inferred from the distribution of activating and inhibiting histone modifications. The partition 5.1.2, on the contrary, is less accessible, suggesting that it contributes mostly to the structure formation. Finally, the distributions of RNA polymerases II and III (S3F and S3G Fig) complement the picture of the potential functional involvement of different partitions in chromosome 17. RNA polymerase II (POL2), crucial component of mRNA synthesis, is distributed quite evenly in both euchromatic and heterochromatic partitions (except the high level in 2.2.1). The synthesis of tRNA, 5S rRNA, and small RNAs through the action of RNA Polymerase III (POL3) is distributed in a more specific way across different partitions. The POL3 signal is high in euchromatic partitions 1.2.2, 4.1.2, 4.2.3, in mixed 4.2.1 and 5.3.1, as well as in some heterochromatic ones (4.1.4, 4.1.5, 5.1.2, 5.1.3, and 5.2.2).

### The whole-genome interactions between partitions of all chromosomes

Peculiarities in distributions of epigenetic factors, DNA accessibility, and RNA polymerases revealed in the analysis of individual chromosomes should be further considered in the framework of whole-genome organization, exploring the interplay between intra- and inter-chromosomal interactions in the regulation of gene expression. To this end, we moved from single-chromosome analysis to studying the whole-genome effective interaction matrix. Given that chromosomes are spatially segregated into chromosomal territories (CTs), one can approximate the whole-genome organization by merging single-chromosome partitioning schemes at appropriate levels. Using a selected representative level from each chromosome (see Materials and Methods: Chromosome partitioning), we formed a whole-genome description with 539 partitions, with an average partition size of about 5Mbp (S2 Table).

**Fig 5:**
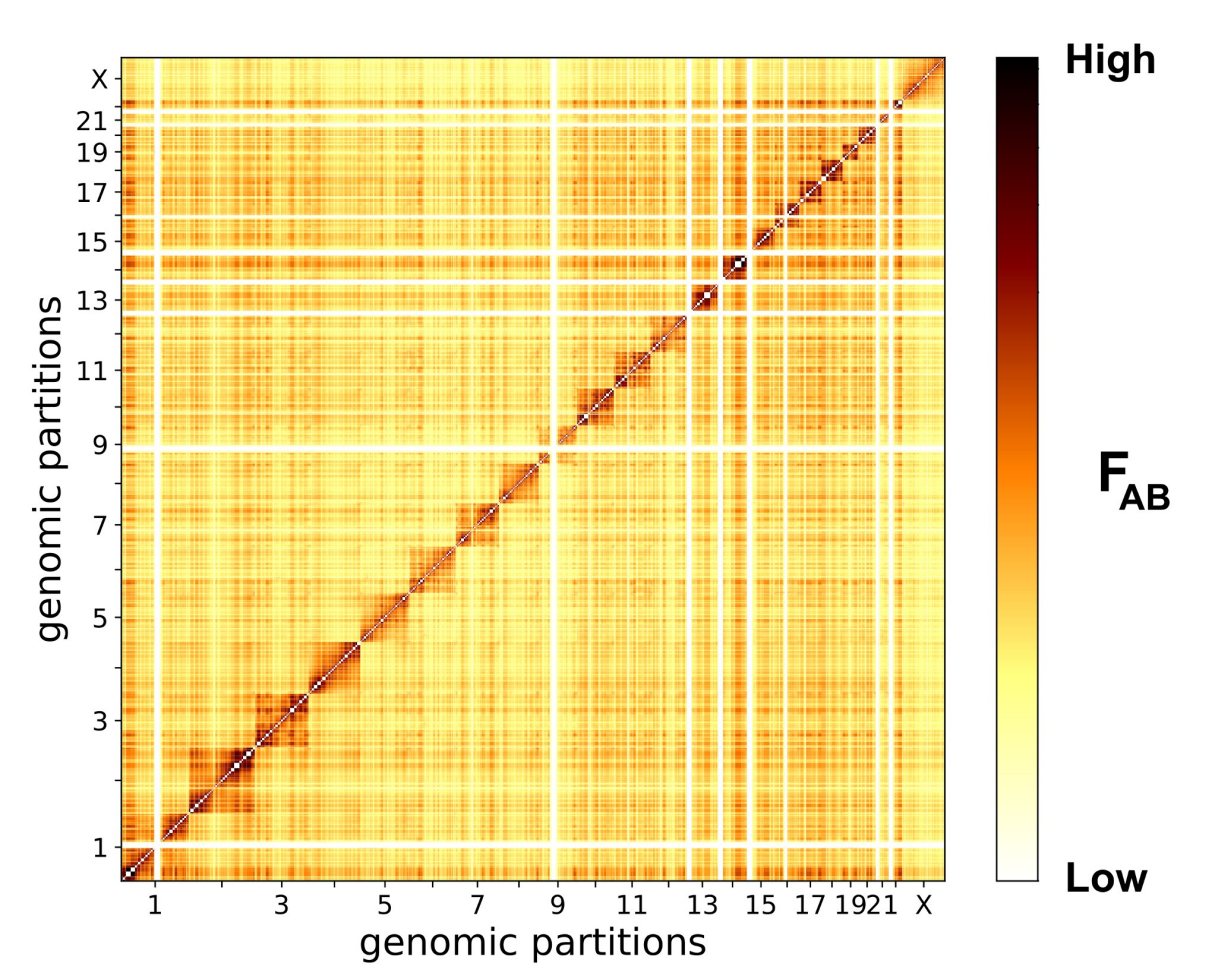
Matrix of whole-genome effective interactions between 539 partitions. Effective interactions between partitions, calculated according to Eq 12 (see Materials and Methods) and plotted on a logarithmic color scale. Massive interactions are formed by chromosomes 14–20, 22, and 1 (clusters of dark pixels), whereas chromosomes 4, 5, 9, 21, and X are not involved in many interactions (lighter pixels). To construct a representation of inter-chromosomal interactions via partitions of comparable sizes, we used partitions of the third level of hierarchy in chromosomes 1–12 and X, second level in chromosomes 13–21, and first level in chromosome 22.

**Fig 6:**
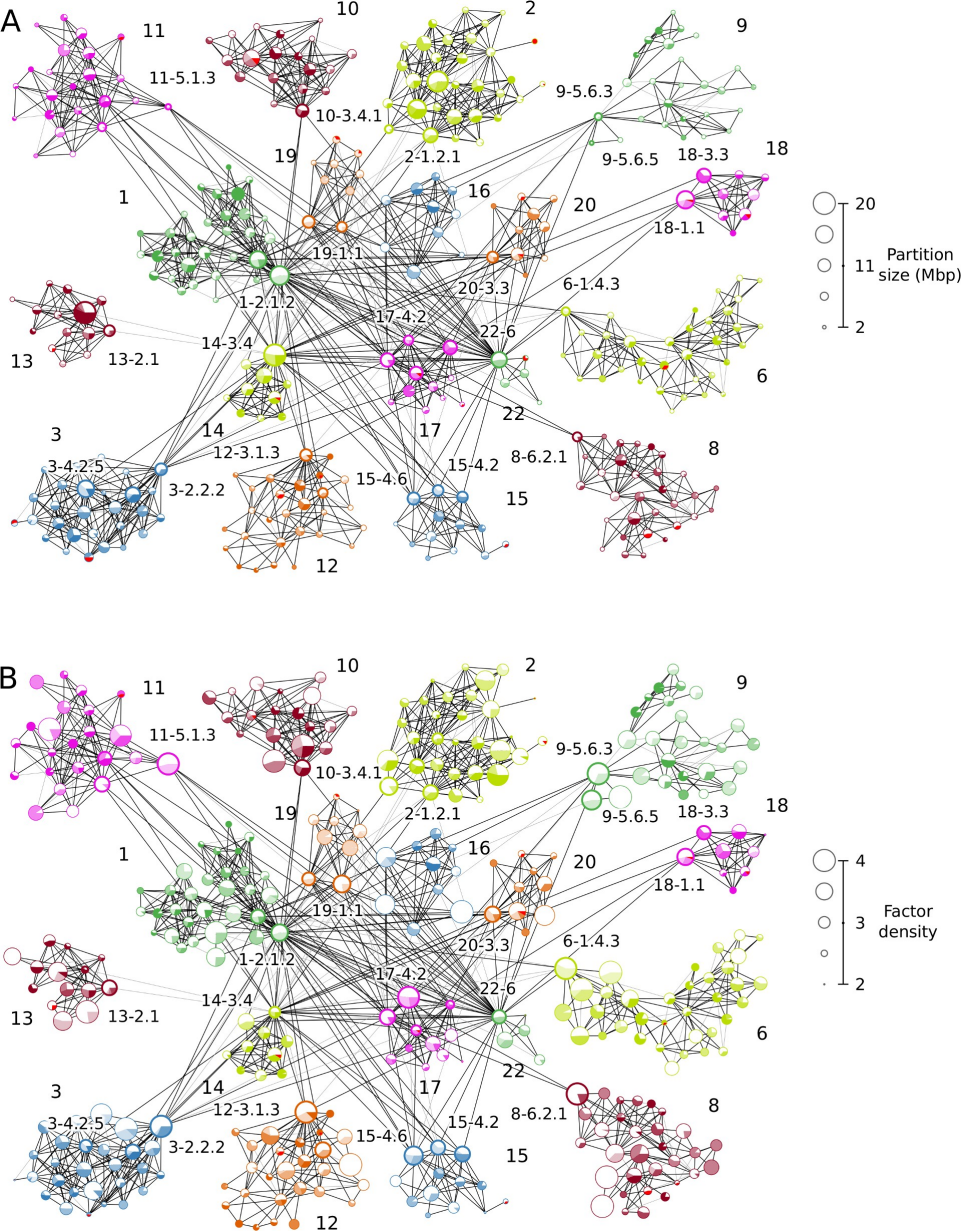
The major cluster in whole-genome partition interactions. The nodes represent (A) partition sizes and (B) CTCF Z-scores. Tight sub-clusters of partitions show chromosomal territories, which are differentiated by the node colors characteristic for different chromosomes. Black edges between nodes represent scaffold-layer interactions, and grey edges, layer 1 interactions (see also S4 Fig and corresponding explanations on the classification of effective interaction strengths in “Data sets, processing, and visualization” of the Materials and Methods). The chromosomes that are not shown here form only single-chromosome clusters that do not strongly interact with chromosomes of the major cluster.

The matrix of effective interactions between chromosomal partitions (Fig 5) provides a general view of the overall physical interactions in chromatin. It shows that chromosome 1 and small chromosomes (14–20 and 22) massively interact with others, while chromosomes 4, 5, 9, 21, and X appear to be relatively isolated from the rest of the genome (Figs 5 and 6). Several partitions form consistently stronger intra- and inter-chromosomal interactions with other partitions. We classified interaction strengths into 5 layers with equally-spaced threshold values: the scaffold layer is the strongest, followed by layers 1, 2, etc. (see also Materials and Methods for the definition of the interaction strength at different layers).

Fig 6A shows the major cluster in the whole-genome partition set: partitions from different chromosomes form tight sub-clusters highlighted by color and marked by chromosome labels. All displayed partitions are linked by the two strongest layers of interactions (scaffold interactions are represented by black edges, and layer 1 by grey edges).

It is easy to see that most of the intra-chromosomal contacts and some inter-chromosomal interactions are established on the scaffold layer, giving rise to a structural foundation for genome-wide architecture (Fig 6). Specifically, chromosomes 1, 14, 16, 17, 19, 20, and 22 are densely interconnected, while other chromosomes in the major cluster are linked to them via only a few interactions. Partitions 1–2.1.2, 14–3.4, and 22–6, for example, act as contact hubs between these massively interacting chromosomes and others. On the other hand, partitions such as 3–2.2.2, 8–6.2.1, 10–3.4.1, connect less-strongly interacting chromosomes to the strongly interacting ones (see S3 Table for interaction strength layers for these interactions between chromosomes). Notably, chromosomes 1 and 2, the two largest ones (about 250Mbp each), behave differently in the context of the whole-genome interactions. While chromosome 1 serves as a hub in the interactions between the highly- and less-interacting chromosomes, chromosome 2 does not show many interactions with other chromosomes (Fig 6A). Turning to functional regulation, most of the partitions involved in significant inter-chromosomal interactions exhibit higher densities of several epigenetic factors, such as CTCF (Fig 6B), H3K9ac (S5A Fig), and DNase accessibility (S5B Fig). These partitions may participate in the formation of active epigenetic compartments facilitated by the structural role of CTCF [65]. Active processing of genomic information taking place in these structures is regulated by the activating histone modifications (H3K9ac) and transcriptional repressors (CTCF). The opposite trend is observed for partition 14–3.4, which is coupled with a higher density of the silencing H3K9me3 histone modification. Therefore, partition 14–3.4 and its interactions with partitions in other chromosomes, for instance 10–3.4.1, with low activating factor densities may indicate the formation of dense structural heterochromatin and/or silencing facilitated by Polycomb bodies [1, 66].

### Correlation between epigenetic signal and effective interactions

To evaluate how the distribution of epigenetic signals may be associated with interaction between partitions, we calculated correlations between effective interaction strengths and the expected enrichment of factor densities across partition pairs that are mostly euchromatic (EC) or heterochromatic (HC). The enrichment of factor densities is estimated here as the product of factor densities per partition (S12 Fig). To obtain the strongest signals, we limited our consideration to interactions between partitions that are dominated by either eu- or heterochromatin (see legend for S12 Fig for the definition of EC and HC partitions): EC-EC pairs (S12A Fig), HC-HC pairs (S12B Fig), and EC-HC pairs (S12C Fig). Despite the relatively weak correlations, general trends appear to be quite clear, with the strongest ones seen between euchromatic (EC-EC) partitions (S12A Fig). Transcription factors CTCF and RAD21 are always positively correlated, as well as POL2 in EC-EC (S12A Fig) and EC-HC (S12C Fig) pairs, whereas POL3 shows no correlation. The positive correlation for CTCF and RAD21 with effective interaction strength agrees with current literature on the role of CTCF and cohesin in mediating chromatin structure through looping interactions [65, 67, 68]. Stronger interactions between EC partitions appear to be linked to higher transcriptional activity, as suggested by the positive correlation with active histone modifications and POL2. Absence of correlation for HC-HC pairs in the case of POL2 can be related to the fact that transcriptional activity is suppressed in heterochromatin. Potential active involvement of interacting euchromatic partitions in the formation of transcription factories is corroborated by the most pronounced correlation observed for DNA accessibility in pairs of euchromatic partitions (S12A Fig). Activating histone modifications, except for H3K4me3, show positive correlations in all types of interacting partition pairs. Interestingly, silencing histone modifications appear also to be weakly correlated with effective interactions between partitions.

### Partitioning analysis on biological replicates and other cell lines

The original partitioning analysis was performed on the GM12878_primary (B lymphoblastoid) Hi-C dataset by Rao *et al*. [23] (GEO accession GSE63525). We also applied our model to four other datasets: GM12878_replicate (a biological replicate of GM12878_primary dataset), IMR90 (lung fibroblast), HUVEC (umbilical vein endothelial cells), and HMEC (mammary epithelial cells). Our goal in this analysis was two-fold: (i) to benchmark robustness and reproducibility of the method using the replicate dataset; (ii) to examine the sensitivity of the method in detecting alterations in chromatin organization in different cell lines, associated with corresponding genome functional states and gene expression levels. S13 Fig shows side-by-side comparisons of the partitioning schemes for GM12878_primary and the other datasets, and S6 Table shows some indicative statistics comparing the results from each case. First, we observed a high consistency between the biological replicates of GM12878: S13A Fig shows that the partitioning was highly consistent between the two sets of Hi-C data, with partition boundaries being identical in most cases, resulting in the high Rescaled Mutual Information (RMI) of 0.70 (see Chromosome partitioning in Materials and Methods for definition of RMI). The composition of the major cluster was also largely identical. Comparing the results from other cell lines, we observed significant differences: IMR90, HUVEC and HMEC cells each had significantly shifted partition boundaries compared to GM12878_primary, leading to lower RMI values of 0.39 to 0.48. The major-cluster structures in these cell lines are also significantly different (see S16 Fig for IMR90 and HUVEC), especially that of HMEC, where no strong inter-chromosomal interactions were observed between partitions, and the chromosomes remained isolated in the whole-genome network. Notably, in both IMR90 and HUVEC, a large partition on chromosome 9 forms extensive inter-chromosomal interactions: the overlapping region (chr9:1268000000–1412500000) contains two genes (OLFM1 and MVB12B) with the RNA-expression profiles different from that of GM12878. The MVB12B (a component of endocytic protein system [69]) gene is activated in both IMR90 (lung fibroblast) and HUVEC (umbilical vein endothelial cells) cell lines, and OLFM1 (lung cancer marker [70]) in IMR90, while both genes are silenced in GM12878. These preliminary observations call for future in-depth investigation of the structural basis, functional mechanisms, and specifics of epigenetic regulation behind the observed differences between cell types.

### Affinity between partitions as an indicator of associations and potential functional interactions in the genome

**Fig 7:**
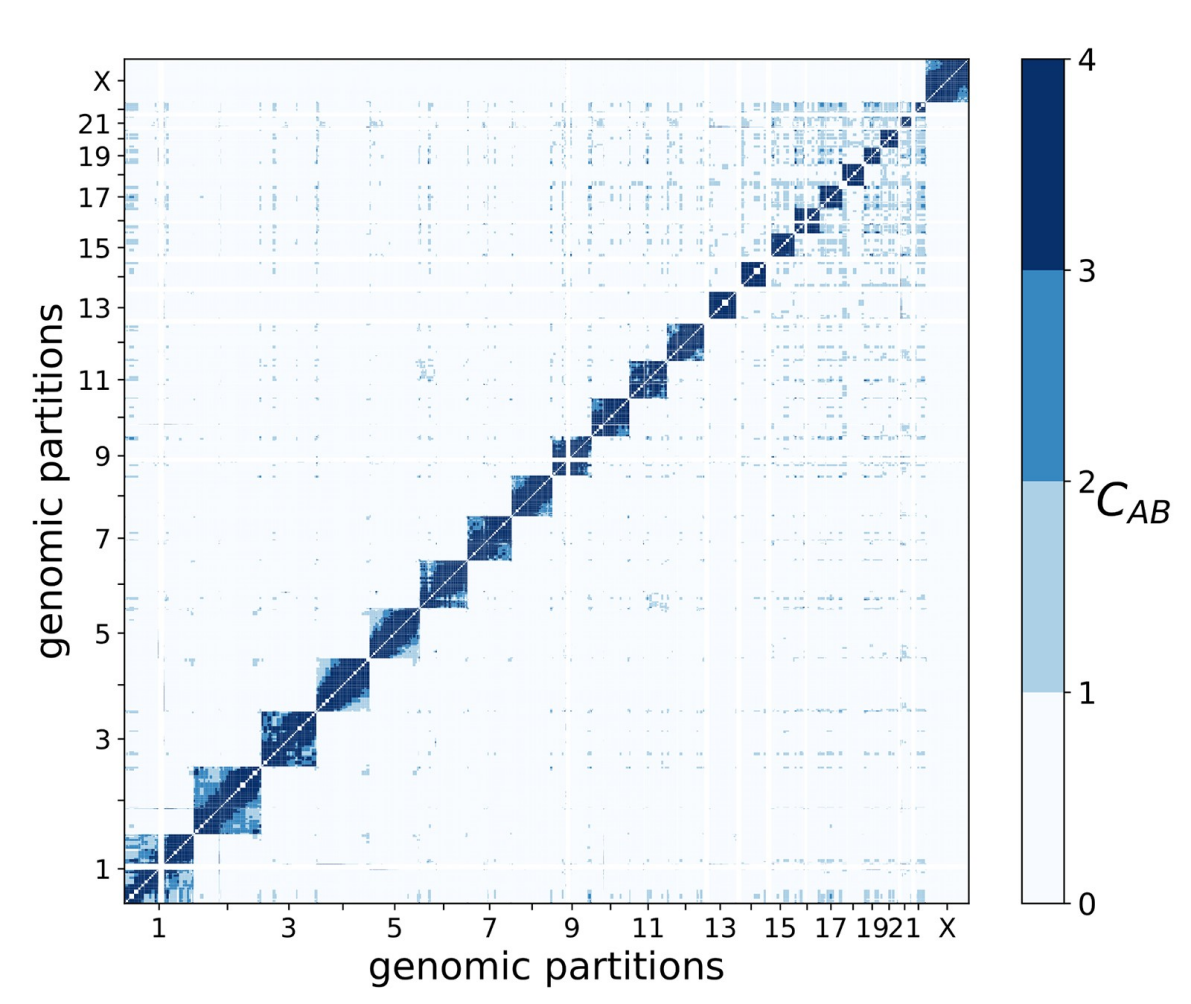
Matrix of the whole-genome affinity between 539 partitions. A color gradient from white to blue is used to show the affinity change from low to high.

While effective interactions between partitions characterize the overall architecture of genome organization, it may not fully discriminate functionally relevant interactions between chromosomes and their parts. Indeed, most partitions are presumably in constant motion within the nucleus, and as Hi-C experiments are typically conducted on unsynchronized cell populations, effective interactions capture the average contact probability arising from both random diffusion and specific transient interactions. Therefore, in addition to effective interactions, the affinity between partitions was also calculated, which reflects how the observed interaction frequency differs from the expected frequency (from random diffusion), because of possible associations between partitions. Defined as the ratio between observed and expected contact probabilities between pairs of partitions (see Eq 15 in Methods), the affinity is indicative of the degree of association between partitions, and high affinity values may serve as a manifestation of biologically-relevant contacts. Fig 7 contains the whole-genome matrix of pairwise affinities (blue: high affinity, white: low affinity) between corresponding partitions. Like the observations in the whole-genome effective interaction matrix (Fig 5), the largest chromosomes 1 and 2 exhibit different behavior, with chromosome 1 containing partitions with high affinity to those in several other chromosomes (especially with chromosomes 14–22) and chromosome 2 generally showing low affinity to partitions in other chromosomes. Smaller chromosomes 14–22 form more, presumably functional, contacts with each other, compared to other chromosomes. At the same time, the number of partition pairs with high affinity is much lower than number of pairs with significant effective interactions (compare Fig 5 and Fig 7). In total, we observed 687 high-affinity pairs (S4 Table), which are seemingly crucial for whole-genome structural organization and function.

**Fig 8:**
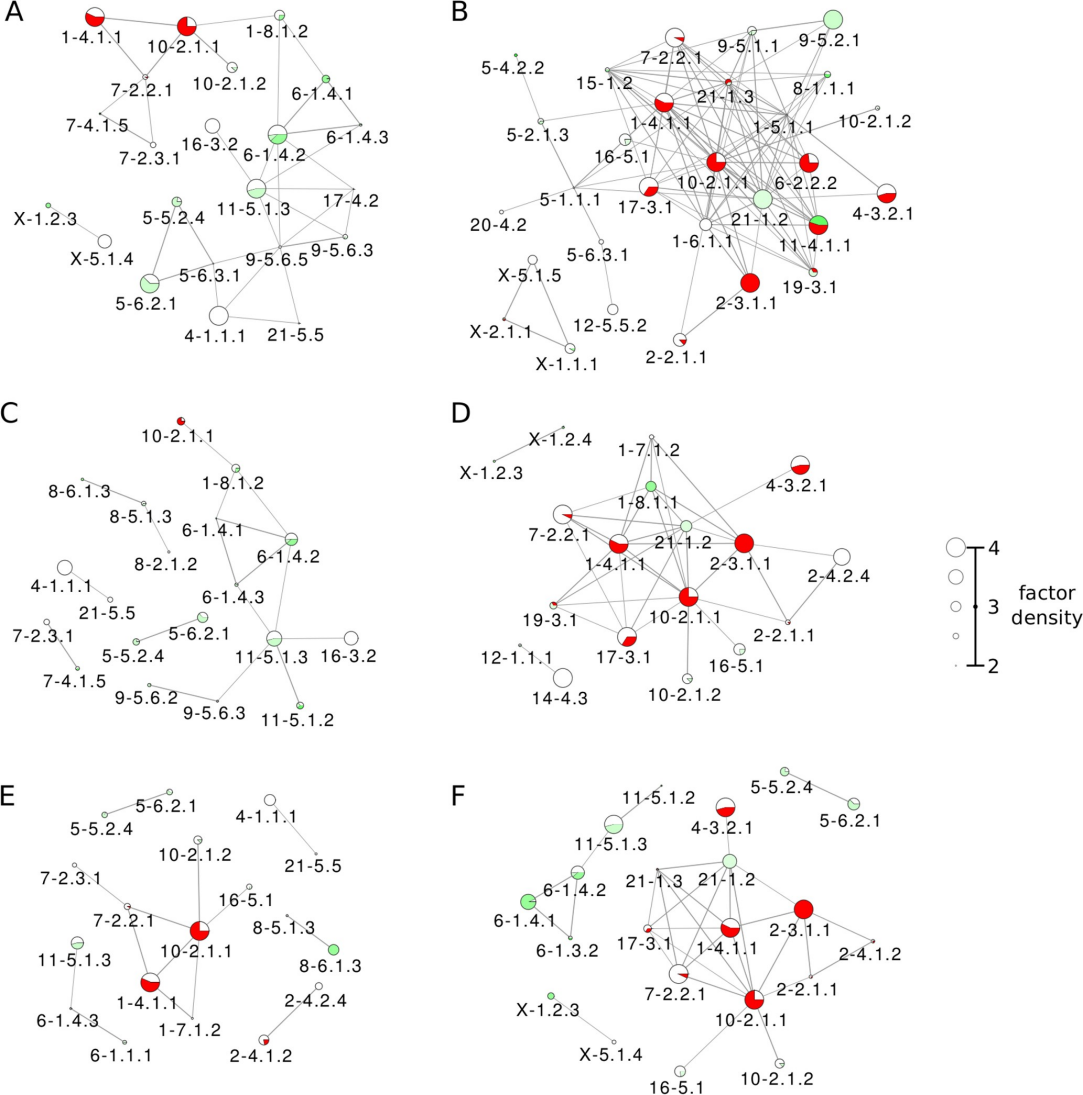
High-affinity clusters enriched in various histone modifications. Node sizes represent the factor Z scores, and edge widths represent affinity values. The following histone modifications are considered; (A) H3K9ac, (B) H3K9me3, (C) H3K27ac, (D) H3K27me3, (E) H3K4me1, and (F) H3K4me3. In each case, only partitions with factor Z-scores above 2 and only edges connecting partition pairs with high affinity ***C*** > **2** are shown.

Interestingly, several large partition pairs (>2Mb) with high effective interactions and affinity are located in the telomeric regions of corresponding chromosomes (yellow cells in S5A Table), having moderately high densities of epigenetic/transcription factors and increased DNA accessibility (S5A Table). Two other groups of partitions with high affinities are characterized by smaller partition sizes and highly elevated concentrations of various transcription factors and epigenetic modifications (S5B Table): (i) pericentromeric partitions (red cells in Table) show high concentrations of activating (H3K4me3) and silencing (H3K27me3 and H3K9me3) histone modifications and high levels of POL3 and RAD21; (ii) telomeric partitions (yellow cells in Table) show strongly increased concentrations of all activating histone modifications, POL2, and CTCF, as well as high DNA accessibility. This separation between types of activating histone modifications, transcription factors, and DNA accessibility in centromeric and telomeric regions signals a specificity of functional interactions between partitions with high affinities to each other. Examples of partitions involved in high-affinity interactions and characterized by the over-representation of different epigenetic factors and modifications are collected in Fig 8 and S6 Fig, where high-affinity clusters of partitions enriched in these epigenetic marks are plotted.

A comparison of the inter-chromosomal interactions in the major cluster of effective interactions (Fig 6) with interactions in affinity clusters (Fig 8 and S6 Fig) highlights several relatively-small partitions, *e*.*g*. 9–5.6.3, 9–5.6.5, and 11–5.1.3, that act as junctures between different chromosomes. These partitions yield increased density of CTCF along with other juncture-partitions (3–2.2.2, 6–1.4.3, 8–6.2.1 to name a few), pointing to the potential importance of these partitions in whole-genome structural organization. This inference is further supported by multiple interactions detected for partitions 8–6.2.1, 9–5.6.5, and 11–5.1.3 in the CTCF affinity graph (S6D Fig). Additionally, H3K9ac (Fig 8A) and H3K27ac (Fig 8C) affinity graphs hint at the functional importance of some of these partitions: the central part of the H3K9ac graph is formed by partitions 9–5.6.3, 9–5.6.5, and 11–5.1.3, while 9–5.6.3 and 11–5.1.3 are also present in the H3K27ac graph.

Focusing on individual epigenetic factors, the activating H3K4me3 mark links more partitions than the activating H3K4me1 histone modification. The silencing H3K9me3 histone modification functionally links many centromeric partitions, whereas the activating H3K9ac modification works in both mostly euchromatic and mixed euchromatic/weakly-heterochromatic non-centromeric regions. Similarly, the activating H3K27ac modification affects mostly non-centromeric partitions, unlike the very active silencing H3K27me3, for example, in partitions 1–4.11.1, 2–3.1.1, and 10–2.1.1 (Fig 8). These partitions are also characterized by the high levels of POL3 (S6B Fig) and RAD21 (cohesin, S6C Fig), whereas the insulator CTCF links several euchromatic partitions across different chromosomes (S6D Fig).

It is evident that centromeric partitions 1–4.11.1, 2–3.1.1, and 10–2.1.1 are enriched with almost all regulatory factors (see Fig 8 and S6 Fig), yielding high affinities to other partitions and pointing to important functional interactions and intense regulation taking place in these partitions. Interestingly, while activating histone marks (Fig 8A, 8C, 8E and 8F) are dominant in several euchromatic partitions, these marks are also present in partitions containing large sections of heterochromatin and centromeres, which are commonly associated with dense packing and transcriptional repression. Similarly, silencing histone marks (Fig 8B and 8D) are dominant not only in heterochromatic and centromeric partitions, but also in some partitions that are mostly euchromatic. Furthermore, dominating regions for the transcription factors CTCF and cohesin (S6C and S6D Fig) appear to have significant overlap with activating and silencing histone marks, respectively. These overlaps show the complexity of functional interactions in chromatin, even at the coarse-grained level of partitions: opposing factors are found acting in the same regions, allowing for switching between transcriptional states in response to other biochemical cues.

## Discussion

We proposed here a computational framework for exploring chromatin organization based on Markov State Modelling of chromatin interactions. Given the multilevel hierarchical packing of chromatin, we introduced a reduced description of the complex network of chromatin interactions and its organization via interactions between structural units at different levels of hierarchy. By interpreting Hi-C data as a pairwise contact energy landscape, a Markov State Model approach was used to explore the chromatin interaction network through the random walk of a probe. While steady-state distributions obtained from the Markov process of randomly-moving molecules can serve as a measure of the chromatin accessibility for epigenetic factors [71], taken alone they describe neither the genome architecture, nor structural and functional interactions between genome partitions and regulatory factors. In this work, analysis of the Markov State Model under thermal annealing shows the key role played by the ruggedness of the contact energy landscape in shaping chromosome structural organization. Specifically, metastability analysis of the Markov State Model associated with the chromatin interaction network allowed us to identify levels of structural hierarchy and to observe structural units—partitions of different scales. These structural partitions serve as a coarse-grained description of chromosomes, which form the basis for introducing the notion of intra- and inter-chromosomal network of effective interactions. The analysis of effective interaction networks across levels of hierarchy in individual chromosomes shows that chromosomes adopt highly varied topologies. While the lower levels reveal an overall architecture of the folded chromosome, the higher levels can provide structural details in relation to functional organization and regulation of gene expression.

Biological insight on the structural organization of chromosomes can be obtained with our method by considering peculiarities in the distributions of transcription and epigenetic factors in eu- and heterochromatic partitions in relation to interactions between them. Partitions at the highest levels of hierarchy may be seen as analogous to TADs, or to the so-called A/B (active/inactive) epigenomic compartments [72]. In the future, with the development of common standards and benchmarks by the community, it would be important to compare results and insight obtained from various genomic segmentation approaches. In this work, however, we based our analysis on the obtained sets of partitions, showing how studying distributions of activating and silencing histone modifications in these partitions can help to understand the role of structural organization of chromosomes in the regulation of gene expression.

Shifting our focus from structural analysis of individual chromosomes and the functional involvement of partitions to whole-genome architecture, we considered the set of 539 partitions obtained at high levels of hierarchy in corresponding chromosomes with effective interactions between them, which were obtained by adopting a fast “mean field” approximation. In the context of the “partition space”, an analysis of genome-wide effective interactions provides a blueprint of inter-chromosomal contacts, showing that despite the strong crowding of partitions in chromosome territories, most chromosomes are significantly connected with each other, giving rise to a bulky cluster in the core of the effective interaction network. The strongest interactions were observed between chromosomes 14–22, which are characterized by small chromosome sizes. Heterochromatic partitions are apparently mostly involved in the formation of chromosomal territories, interacting within the corresponding chromosomes and providing structural integrity, and showing only low levels of activating factors. On the other hand, most of the inter-chromosomal juncture partitions, while relatively small in size, are enriched with CTCF, H3K9ac, and DNase-Seq, which may lead one to conclude that these partitions are involved in the formation of inter-chromosomal active epigenomic compartments [72]. Correlations of effective interactions between partitions with distributions of epigenetic factors in these partitions show that: (i) most active regulation apparently takes place in pairs of interacting euchromatic partitions; (ii) DNA accessibility, CTCF and activating histone modifications H3K4me1, H3K9ac, and H3K27ac are major potential contributors in the regulation of genome function. Additional biological insight was obtained by determining partitions that may form transient function-related contacts, thereby triggering alternate chromatin states. To this end, the affinity measure was introduced here to evaluate the level of association between partitions, so as to identify partitions with functionally-related interactions. Irrespective of the effective interaction strength, high affinity between partitions point to the mutual functional involvement of corresponding partitions. Since different factors are likely to play dominant roles in different genomic regions, our affinity analysis is complementary to the concept of chromatin assortativity introduced by Pancaldi et al. [44], which may identify epigenetic factors associated with multiple high-affinity communities across the whole genome.

There are different challenges in extending the original analyses of the Hi-C data to exploring the structure-function relationships in the genome. Several previous studies using a hierarchical clustering approach for the analysis of Hi-C data are based on the *a priori* assumption of the existence of structural hierarchy in chromatin [38–40]. While the work by Boulos et al. is free from such an assumption [37], it employs a tunable scale parameter in establishing the hierarchy. Our approach is based on an energy landscape representation of the chromatin interaction network, which is formalized and explored via a Markov State Model. Metastability analysis of the Markov State Model allows one to detect natural levels of hierarchy in chromatin structure. The novelty of our approach does not free it, however, from certain limitations. For example, the “mean-field” approximation for coarse-graining of whole-genome interactions, rooted in the computational challenge in calculating committor probabilities on very large networks, is currently a necessary step for processing massive whole-genome datasets. On the other hand, because of its rapid computational time, this approximation allows us to explore higher resolution Hi-C datasets and to obtain partitions of smaller sizes and, ultimately, uncovering higher levels of hierarchy. Further, as the rugged morphology of the energy landscape is key to determining chromosomal partitions, the effect of noise associated with the Hi-C data is of critical importance. For example, all contact frequencies and derived quantities are affected by the characteristic noise, since most Hi-C experiments are conducted on unsynchronized cell populations. While a possible solution would be to study single-cell Hi-C [73] data, which shows great promise in capturing differences between transient states in chromatin organization, the current protocol yields too few interaction pairs for a meaningful analysis of the interaction network. Although specialized variants of the Hi-C protocol, such as capture Hi-C (cHi-C) [74], do not provide a full view of the physical organization of chromatin, they can nonetheless be useful in targeting specific subsets of genomic loci, such as promoters. The biological implications of our analysis, particularly on the relationship between factor enrichment and effective interaction strength and affinity, may also be strengthened by incorporating additional experimental approaches, such as ChIA-PET [75] and HiChIP [76], which identify interacting genomic elements that are concurrently associated with specific binding proteins. Genome architecture mapping (GAM), a newly devised experimental protocol that determines the frequency at which genomic loci lie on the same spatial plane by sequencing fragments isolated in cryosections of the nucleus [77], can be a great source of constraints for future 3D whole-genome reconstruction.

To conclude, there is no doubt that scientific interest in chromatin structure will continue to drive the development of a variety of specialized experiments and computational approaches in the field of 3D genomics. The method presented here, aimed at detecting and characterizing the hierarchical organization of chromatin, is a step towards unravelling causal relationships in chromatin structure and dynamics of function-related transient molecular phenotypes. The great potential of new experimental data combined with constant methodological improvement are critical in the quest for a more detailed understanding of chromatin architecture, 3D reconstruction, dynamics, and epigenetic regulation.

## Materials and methods

### Ethics statement

No human or animal subjects and/or tissue were used in the work.

Ethics rules of the Bioinformatics Institute, A*STAR were followed during the work on the project and preparation of the paper.

### Markov State Model of the genome

In the following, a Markov jump process is introduced to describe a random walk in the chromatin interaction network, where a probe connects pairs of interacting genomic loci, which represent the states of the Markov State Model (MSM). We first focus on a single chromosome *c* and denote the corresponding matrix element of Hi-C counts *f*_*ij*_ for a pair of loci (*i*,*j*). We define a pairwise interaction pseudo-energy *E*_*ij*_ = −ln*f*_*ij*_, which characterizes a strength of interaction between a pair of loci (*i*,*j*): the higher the observed counts the more stable the corresponding interactions (lower pseudo-energy) are. Therefore, a Maxwell-Boltzmann probability distribution of counts is defined as

$$\pi_{ij}\left(\beta \right)=\frac{1}{Z\left(\beta \right)}\exp\left(-\beta E_{ij}\right)$$ (1)

where *Z*(*β*) = ∑_(*i*,*j*)∈*c*_ exp(−*BE*_*ij*_) is the partition function and *β* is the thermal parameter (inverse of a temperature). Eq 1 evaluates the joint interaction probability of a pair of loci (*i*,*j*), which can be modulated by the thermal parameter *β*. For low values of *β* (high temperature), the interaction energies tend to contribute equally in the exponential, whereas for high values *β* (annealing, low temperature) only the highly interacting loci contribute significantly in the exponential. The transition probability associated with the Markov jump process is defined by the following conditional probability

$$p_{ij}=\frac{\pi_{ij}}{\mu_{i}}$$ (2)

where

$$\mu_{i}=\underset{j\in c}{\sum }\pi_{ij}$$ (3)

is the probability for the genomic locus *i* to form any interaction in chromosome *c*. Additionally, *μ*_*i*_ is the steady state distribution of the transition matrix *p*_*ij*_. The transition probability matrix in Eq 2 uniquely identifies a discrete time Markov jump process that governs the trajectories of a random walker across the state space. A random walker is interpreted as a probe particle traveling between genomic loci, for instance a protein such as a transcription factor. Accordingly, the steady state distribution *μ*_*i*_ can be interpreted as a distribution of probes in a locus *i*, whereas the distribution $\pi_{ij}^{c}$ in Eq 1 describes an undirected flux of probes connecting the loci *i* and *j*.

The kinetic distance between pairs of loci is the mean first passage time (MFPT), the mean number of discrete steps *τ*_*ij*_ between two different genomic loci (*i*,*j*) in chromosome *c*, which is obtained by solving the system of equations [78]

$$\tau_{ij}=p_{ij}+\underset{k\neq i,j}{\sum }p_{ik}\left(1+\tau_{kj}\right)$$ (4)

If the departure and arrival states coincide, the MFPT is called mean recurrence time MRT *τ*_*i*_, which gives the mean time for a walker to return to its initial state *i*. The MRT is obtained from the MFPTs in Eq 4 via the formula

$$\tau_{i}=p_{ii}+\underset{k\neq i}{\sum }p_{ik}\left(1+\tau_{ki}\right)$$ (5)

S7 Fig shows the MFPT matrices (with the MRTs in diagonal) for chromosomes 1, 17, and 20 at *β* = 1 (left) compared to annealing condition at high *β* (right). A separation of time scales emerges upon increasing the *β* parameter, which is reflected in the partitioning of the MFPT matrices. The squares depicted in the annealed MFPT matrices identify sets of pairs (*i*,*j*) with a similar *τ*_*ij*_, which are the results of the partitioning in the network of interactions. S8 Fig shows the MFPT matrices for all chromosomes at corresponding high *β*. For each chromosome, the value of *β* was chosen as large as possible to observe the fine partitioning structure in the chromosome, while at the same time avoiding singular values in the calculations of the MFPTs and MRTs. The values of *β* used are listed in S1 Table.

### Metastability and optimal hub sets

#### Metastability index

The major goal of associating a Markov process with Hi-C network data is to use a random walk as a tool to explore chromosomal structure. By traveling across the chromosome, a random walking probe connects pairs of genomic loci. Among the loci that the probe encounters, some are more special than others. These special loci are the hubs in the underlying network structure that correspond to highly interacting and centrally located loci, which implies that they act as attractors of the MSM. Typically, a probe departing from a non-hub locus will likely hit a hub and stay around it, whereas a probe departing from a hub locus will likely stay around it rather than hitting another hub. This property points to the metastability of the corresponding Markov process, as described elsewhere [59]. The metastability of the MSM that is associated with a chromatin interaction network is reflected in the multiple highly-connected hubs that form the cores of partitions of different sizes. In the following we present an optimization method for identifying hub sets in metastable networks. The method was previously introduced and justified in the context of metastable dynamical systems [59], and is adapted here to the context of chromatin.

The method is essentially a complexity reduction scheme: to identify the hub set M that best captures the metastable dynamics of the network, a metastability index $\rho_{M}$ is optimized across all possible candidate hub sets. Briefly, the metastability index is defined as the ratio of two distinct probabilities: the probability for a walker to connect two hub loci (as small as possible in a metastable hub set) and the probability for a walker to hit any hub locus (as large as possible in a metastable hub set). In mathematical terms, for a given chromosome *c* the metastability index can be defined as

$$\rho_{M}=\frac{\max_{i\in M}\;\max_{j\in M\backslash \left\{i\right\}}\Gamma_{ij}}{\min_{i\notin M}\;\max_{j\in M}\Gamma_{ij}}$$ (6)

where *Γ*_*ij*_ is a pairwise committor probability, which is the probability for a probe departing from locus *i* to hit locus *j* before returning to *i*, and it is expressed in terms of the MFPT and MRT values in Eqs 4 and 5 [59]

$$\Gamma_{ij}=\frac{\tau_{i}}{\tau_{ij}+\tau_{ji}}$$ (7)

The probabilities *Γ*_*ij*_ and the metastability index $\rho_{M}$ are functions of the thermal parameter *β*. In particular, upon annealing conditions (high *β*) the time scales associated with the MSM random walks increase exponentially with *β*, which, in turn, increases metastability. As pointed out above, *β* values that are too high can lead to numerical instabilities which can result in singularities in the calculation of the MFPTs and MRTs and, consequently, on the pairwise committor probability *Γ*_*ij*_ of Eq 7. Thus, for each chromosome, the highest integer value of *β* was chosen under the condition that singular values in the MFPTs calculations are avoided.

#### Optimization procedure

The procedure for optimization of the metastability index $\rho_{M}$ and for finding the optimal hub sets for each chromosome starts from constructing putative trial hub sets of increasing size *n*, which are, then, processed via a Monte Carlo (MC) minimization/optimization scheme to find the hub set of size *n* with the lowest metastability index. The procedure consists of three steps. **Step 1.** The first hub set {*a*,*b*} is putatively constructed such that *a* = argmax_*i*_ *μ*_*i*_ (see Eq 3), i.e. the locus with the highest density of probes, and *b* = argmin_*i*_ *ρ*_{*a*,*i*}_, as *b* minimizes the corresponding metastability index. **Step 2.** The MC scheme is applied to the current hub set by combining two types of move sets in order to sample trial hub sets. In the first move set type, a random hub locus *a* is replaced according to the rule *a*^†^(*a*) = argmax_*i*_ *Γ*_*ai*_, whereas in the second type *α* is replaced with a random locus that is not present in the current hub set. Since both move sets can, in principle, generate duplicate loci in the trial hub set, the move is rejected and a new one casted if duplicate loci are generated. The metastability index is calculated on the trial hub sets, which are accepted or rejected according to a Metropolis criterion. The number of iterations for the MC optimization is 500. Among all the hub sets sampled, only the optimal one with the lowest $\rho_{M}$ is selected as the hub set of size *n*. **Step 3.** A trial hub set of size *n* + 1 is constructed by adding one more locus to the optimal hub set of size *n*. Subsequently, step 2 is performed to obtain the optimal hub set of size *n* + 1.

Steps 2 and 3 are iterated until the upper limit for hub set size is reached. The upper limit of hub set size was set to 50 for each of the chromosomes as this was sufficient for defining 3 levels of hierarchy for each chromosome (see section Chromosome partitioning).

The final goal of the above optimization procedure is to obtain a profile of the metastability index $\rho_{M}$ as a function of set size. S14 Fig shows the metastability index profiles for each of the chromosomes in the dataset calculated for increasing values of the inverse temperature parameter *β*. Each of the profiles exhibits multiple minima that correspond to hub sets of different sizes. As discussed below, the hub sets corresponding to minima can be used as starting points for constructing the hierarchy of chromosome partitions.

### Chromosome partitioning

#### Committor probability

From the optimization procedure of the metastability index, a list of optimal hub sets can be obtained for each of the chromosomes. An optimal hub set M is a concise map of the intra-chromosomal network of interactions at a given resolution. The map is accurate as much as it identifies highly interacting regions in the chromosome—partitions. A way to identify *soft partitions* out of the hub set M is to use a committor probability *q*_*a*_(*i*) [59], which is the probability for any locus *i* to belong to the partition defined by hub a∈M rather than that by any other hub in M\{a}, with the normalization $\sum_{a\in M}q_{a}(i)=1$ for any locus *i*. Given chromosome *c* and the hub set $M,\;q_{a}(i)$ solves the following system of equations with boundary conditions

$$\{\begin{array}{l} \sum_{j\in c}L_{ij}q_{a}\left(j\right)=0,\;i\notin M\\ q_{a}\left(i\right)=0,\;i\in M\backslash \left\{a\right\}\\ q_{a}\left(i\right)=1,\;i=a \end{array}$$ (8)

where *L*_*ij*_ is the Laplacian associated with the transition matrix such that *L*_*ij*_ = *p*_*ij*_ − *δ*_*ij*_. The lowercase index *a* indicates both a hub locus in the hub set M and its related soft-partition characterized by the committor *q*_*a*_(*i*). If the hub set M is a good representation of the network, *i*.*e*. $\rho_{M}$ is low, *hard-partitions* can provide a coarse-grained representation of the chromosome (see S9 Fig for illustration of the difference between the concepts of hard and soft partitions). A hard partition is defined when a locus either belongs to it or not, with corresponding committor defined as

$$\theta_{A}\left(i\right)=\{\begin{array}{l} 1,a={\mathrm{argmax}}_{b}q_{b}\left(i\right),i\in A\\ 0,\;\mathrm{otherwise},i\notin A \end{array}$$ (9)

such that $\sum_{a\in M}\theta_{A}(i)=1$ and with the uppercase index *A* denoting the hard partition linked to a hub locus a∈M, which is the set of loci *i* where *θ*_*A*_(*i*) = 1. In this work, we obtain the partition set P linked to the hub set M using the hard-partition scheme.

#### Detecting hierarchical levels in a chromosome

For a given chromosome *c*, since the metastability index $\rho_{M}$ is a measure of how accurately the hub set M represents the intra-chromosomal network of interactions, only sets M corresponding to minima in the profile $\rho_{M}$ are considered. In determining the levels of structural hierarchy for each chromosome corresponding to different hub sets, an operational threshold is used to select the hub sets corresponding to minima with $\rho_{M}<0.8$ (S10 Fig). Ignoring the trivial level *n* = 2, a hierarchy of levels of sizes *n*_*i*_ is established according to the empirical rule *n*_*i*_ ≥ 2*n*_*i*−1_, which ensures that hub sets across consecutive levels are not too similar. For the largest chromosomes 1 and 2, the second-smallest *n* at which $\rho_{M}$ is minimum is chosen as the first level of hierarchy, *n*_1_, in order to have an average partition size in these chromosomes comparable to those of smaller chromosomes.

#### Consistency of the chromosome partitioning

In order to check the robustness of our chromosome partitioning method, the same analysis was performed on two biological replicates of the Hi-C data set [23], and the consistency between partitioning schemes across data sets was tested using mutual information. Briefly, to be consistent, similar portions of chromosomes’ genomic loci should be shared across partitions in the partition sets obtained on the different data replicas. Given chromosome *c* and two partition sets $P_{1}$ and $P_{2}$ corresponding to two replicas of the Hi-C data, we define the normalized mutual information

$$I\left(P_{1},P_{2}\right)=\frac{1}{H\left(P_{1}\right)+H\left(P_{2}\right)}\sum_{A\in P_{1},B\in P_{2}}\nu_{AB}\ln\frac{\nu_{AB}}{\nu_{A}\nu_{B}}$$ (10)

where *ν*_*AB*_ is the normalized fraction of loci that are present in both partitions $A\in P_{1}$ and $B\in P_{2},\;\nu_{A},\;\nu_{B}$ are the normalized fractions of loci that are present in $A\in P_{1}$ and $B\in P_{2}$, respectively, and $H(P_{1})$ and $H(P_{2})$ are the Shannon entropies associated with the partition sets. The normalized mutual information between two data sets is greater than 0.9 for most chromosomes. The consistency between partitioning schemes was also checked across Hi-C data resolutions and upon annealing of the thermal parameter *β*: using the interaction matrices at 25kbp, 100kbp, and 200kbp resolution does not affect the partition boundaries significantly; hard-scheme partition boundaries do not change for *β* ≥ 4 (see S15 Fig). We calculated the mean normalized mutual information between partitioning schemes with random boundaries, which for 2000 random samples resulted in 0.77. Therefore, we report a rescaled mutual information (RMI) between partitioning sets as $RMI(P_{1},P_{2})=(I(P_{1},P_{2})-0.77)/0.23$, which assumes values between 0 and 1 (S6 Table). The RMI is also used here as a quantitative measure of similarity between partitioning schemes across different cell lines.

### Effective interactions between partitions

The optimization of the metastability index upon annealing conditions allows one to obtain an optimal hub set M. It has been shown that in a MSM with a large state space, an optimal hub set and its corresponding partitions can be used for reducing the state space and obtaining a smaller MSM [59]. In the context of our model of chromosome interactions, we introduce a scheme for coarse-graining chromatin structure and quantifying effective interactions between the obtained partitions. In the previous section, we have described the interaction network of a chromosome *c* in terms of a MSM with transition matrix *p*_*ij*_ (Eq 2), representing the probability for a probe to reach locus *j* from locus *i*, with the steady-state distribution of probes *μ*_*i*_ in locus *i* (Eq 3), and with the undirected flux of probes *π*_*ij*_ between loci *i* and *j* defined in Eq 1. Considering the optimal hub set M with its associated committor probability *q*_*a*_(*i*) in the chromosome *c*, the effective flux of probes between any two soft-partitions *a*,*b* is given by

$$F_{ab}=\underset{i\in c}{\sum }q_{a}\left(i\right)\pi_{ib}$$ (11)

where *π*_*ib*_ is the undirected flux of probes between the loci *i* and *b* (Eq 1) in chromosome *c*. In other words, Eq 11 measures the portion of flux between any locus *i* and hub *b* passing through hub *a* (see S2 Fig for illustration of the notion of effective interactions). Eq 11 is an exact calculation on a single chromosome.

We are also interested in evaluating the effective fluxes between hub loci in different chromosomes. However, Eq 11 cannot be simply extended to the whole genome as the committor is by construction *q*_*a*_(*i*) = 0 for any locus *i* ∉ *c*. As computational limitations do not allow us to calculate the exact committor for the entire genome, a mean field formulation of Eq 11 was used to estimate the effective flux between any two partitions *A*,*B* in the genome. To this end, the effective flux between partitions, irrespective of the chromosomes to which they belong is calculated as

$$F_{AB}=\sum_{i\in g}\theta_{A}\left(i\right)\sum_{j\in g}\pi_{ij}\theta_{B}\left(j\right),$$ (12)

where the summations are carried over the entire genome *g*, *π*_*ij*_ is the flux of probes between any pair of *i* and *j* in the genome (Eq 1 with *f*_*ij*_ the Hi-C matrix of paired-end reads of counts is now extended to the entire genome), and *θ*_*A*_(*i*) the hard-partitioning committor defined in Eq 8. The rationale of Eq 12 is to efficiently, though indirectly, estimate the flux between any two partitions A, B in terms of all the intermediate pairwise fluxes *π*_*ij*_. Within the logic of a MSM, the effective fluxes in Eq 12 serve as a measure of chromatin effective interactions.

### Affinity between partitions

Chromosome partitions are obtained from the optimal hub set as a result of the metastability analysis upon annealing conditions. They offer a coarse-grained description of the genome as the interactions between partitions are characterized via effective interaction strengths (Eq 12). Given a genome-wide set of partitions obtained above, a putative reduced model of the major partition interactions can be constructed by directly coarse-graining the matrix of counts *f*_*ij*_ for the entire genome. The observed joint probability of interaction between two partitions *A* and *B* is

$$P\left(A\cap B\right)=\frac{\sum_{i\in A}\sum_{j\in B}f_{ij}}{\sum_{\left(X,Y\right),X\neq Y}\sum_{i\in X}\sum_{j\in Y}f_{ij}},\;A\neq B$$ (13)

where the summation in the denominator is carried out on the pairs (*X*,*Y*) of distinct partitions to ensure proper normalization. Because of the law of total probability, the probability for a partition *A* to be involved in any interaction other than itself is

$$P\left(A\right)=\sum_{Y\neq A}P\left(A\cap Y\right)$$ (14)

which by construction adds up to one over all possible partitions *A*. In general, in the case of independent partitions, namely with no association between them, the relation *P*(*A* ∩ *B*) = *P*(*A*)*P*(*B*) would hold for the interaction probability. Therefore, to provide a measure of the degree of association between partitions, we define the following affinity as

$$C_{AB}=\frac{P\left(A\cap B\right)}{P\left(A\right)P\left(B\right)}$$ (15)

which is a positively defined quantity. This quantity is also known as the observed to expected ratio o/e where *P*(*A* ∩ *B*) and *P*(*A*)*P*(*B*) are the observed and expected probabilities, respectively. In the case of *C*_*AB*_ > 1 where the observed probability exceeds the expected, this is interpreted as a degree of association between partitions, either a contact or functional relationship. On the contrary, if *C*_*AB*_ ≤ 1 observed and expected probabilities either coincide or the expected probability exceeds the observed one. These situations are interpreted as either no association (between partitions *C*_*AB*_ = 1) or dissociation (partitions repel each other for *C*_*AB*_ < 1). Thus, high values of affinity indicate a high degree of association between partitions, suggesting the presence of active binding and/or co-localization mechanisms. Intra-chromosomal pairs show very high affinities, typically with *C*_*AB*_ > 10, while inter-chromosomal pairs have affinities *C*_*AB*_ < 4.

### Data sets, processing, and visualization

In this work, we analyzed 50kbp in-situ Hi-C interaction maps obtained by Rao *et al*. [23] for human B lymphocyte cells (GM12878, two replicates) at both single-chromosome and whole-genome levels (GEO accession GSE63525). Three other datasets listed under the same GEO accession were also analyzed: IMR90 (lung fibroblast), HUVEC (umbilical vein endothelial cells), and HMEC (mammary epithelial cells). Epigenomic data tracks for GM12878 were obtained from the ENCODE Consortium web portal, with signal tracks for transcription factor ChIP-Seq from ENCODE/Stanford/Yale/USC/Harvard, histone ChIP-Seq from ENCODE/Broad Institute, DNase-Seq from ENCODE/OpenChrom (Duke).

Z-scored fractions of epigenetic factors were calculated in order to investigate their distributions within partitions. In the single-chromosome case, for a given signal track density *x*_*f*_(*A*) of factor *f* in a partition *A* of chromosome *c*, the Z-scored density of factor *f* is:

$$Z_{f}\left(A\right)=\frac{x_{f}\left(A\right)-\mu_{f}}{\sigma_{f}}$$ (16)

where *μ*_*f*_ and *σ*_*f*_ are the weighted mean and standard deviation of densities of factor *f* across partitions in the chromosome *c*. For the Z-score calculations on the whole-genome, the weighted mean and standard deviation across all 539 partitions were used.

For the network representation of the effective interactions, the force-directed layout in Cytoscape was used [79] with the force constants parametrized as log*F*_*AB*_, where *F*_*AB*_ is the effective interaction between partitions (Eqs 11 and 12). The node sizes are proportional to the partition size or Z-scored epigenetic factor density, respectively. Only partitions of size larger than 2Mbp are shown. Edge width scales with log*F*_*AB*_ and only interactions above a certain threshold are shown. For intra-chromosomal networks, width of edges is defined according to fixed thresholds of the interaction strength at each level of hierarchy.

In the whole-genome network of effective interactions, given the large number of partition pairs with a wide spread of effective interaction strengths, we classify interaction strengths into discrete levels and ignore weaker interactions. Histograms of the distribution of effective interaction strengths are plotted in S4 Fig, with intra-chromosomal (red), inter-chromosomal (green), and all (blue) interactions shown on the same axis. Layers of successively weaker interactions provide finer details to the interaction network structure (see S4 Fig): (i) Scaffold-Layer interactions are the strongest 2000 interactions, or the top 1.35% of all interactions; (ii) Layer 1 interactions comprise the top 1.35% to 1.5% of all interactions, compared with the Scaffold Layer; (iii) Layer 2 interactions represent the top 1.5% to 1.7% of interactions; (iv) Layer 3 interactions represent the top 1.7% to 2.0% of interactions. In our analysis for the GM12878_primary network, we considered only the scaffold and Layer 1 interactions (the top 1.5% of all interactions) to be significant.

### Hi-C data preprocessing

Before performing the MSM analysis for single chromosomes, a Gaussian Filter (GF) was employed to reduce the effects of sampling noise and systematic errors in Hi-C data: the matrices of raw interaction counts were convolved with a Gaussian kernel. With the interaction matrix at 50kbp resolution, a width parameter in the Gaussian kernel *σ* = 200kbp was used, truncated at 4*σ*. S11 Fig shows a comparison of the raw Hi-C matrices with those after the GF preprocessing on chromosomes 1, 17, and 20. Performing partitioning analysis with and without GF preprocessing showed that both approaches yielded similar hub sets and partitions, and also when *σ* is varied within reasonable bounds, but the optimization on filtered datasets converged more rapidly. The Gaussian kernel width *σ* was chosen to balance between retaining structural information and computation speed: while increasing *σ* improved convergence rate, doing so smears out structural information in the high-resolution interaction matrices. Computation of effective interactions between partitions (Eq 12) is not affected directly by GF as the raw interaction matrices are used for obtaining the *π*_*ij*_ values.

### Software implementation details

The algorithms used in this study are implemented in a freely available Python package ChromaWalker (https://bitbucket.org/ZhenWahTan/chromawalker), built on the standard SciPy stack of libraries (NumPy, SciPy, Matplotlib, and Pandas), using a serial implementation on CPU. The run time for a full genome at 50kbp resolution, on a 3.4GHz Intel Core i7 CPU with 8GB RAM, is approximately 1 week.

## Supporting information

S1 Fig Partition diagrams for all 23 chromosomes derived from 50kbp in-situ Hi-C interaction maps obtained by Rao *et al*. [23] for human B lymphocyte cells (GM12878). Three levels of hierarchy are presented and matched to the linear map of corresponding chromosomes with eu-/heterochromatic bands marked according to Giemsa staining [61].
(PDF)

S2 Fig Illustration of the “mean field” approximation used for inter-chromosomal effective interactions. Given two hub loci *a* and *b* belonging to two different chromosomes, the effective interaction between them is estimated by summing up the fluxes between *a* and *b* passing through all possible pairs of intermediate loci *i* and *j* that belong to these two chromosomes.
(PDF)

S3 Fig Architecture of chromosome 17 at the third level of structural hierarchy combined with epigenetic data (complementary to Fig 4). Edge widths correspond to effective interaction strengths, and node sizes in each panel represent Z scores for the following factors: (A) H3K9me3, (B) H3K27ac, (C) H3K27me3, (D) H3K4me1, (E) H3K4me3, (F) POL2, (G) POL3, and (H) RAD21.
(PDF)

S4 Fig Histograms of effective interactions, for intra-chromosomal (red), inter-chromosomal (green), and all interactions (blue). Vertical red lines show the respective cutoff values for classifying interaction strengths into the scaffold layer, and Layers 1 through 4. Scaffold-layer interactions are the strongest 2000 interactions, or the top 1.35% of all interactions. Layer 1 interactions comprise the top 1.35% to 1.5% of all interactions, compared with the scaffold layer. Layer 2 interactions represent the top 1.5% to 1.7% of interactions. Layer 3 interactions represent the top 1.7% to 2.0% of interactions.
(PDF)

S5 Fig Distribution of various epigenetic factors in the partitions of chromosomes in the major cluster of the whole-genome inter-chromosomal interactions (complementary to Fig 6). (A) H3K9ac, (B) DNase-Seq.
(PDF)

S6 Fig High-affinity clusters in the network of the whole-genome inter-chromosomal interactions enriched in various epigenetic factors (complementary to Fig 8). (A) POL2, (B) POL3, (C) RAD21, and (D) CTCF.
(PDF)

S7 Fig Mean first passage time (MFPT) matrices with the mean return times (MRTs) in the diagonal for individual chromosomes with and without thermal annealing. (A) Chromosome 1, *β* = 1, (B) Chromosome 1, *β* = 9, (C) Chromosome 17, *β* = 1, (D) Chromosome 17, *β* = 9, (E) Chromosome 20, *β* = 1, (F) Chromosome 20, *β* = 9.
(PDF)

S8 Fig Mean first passage time (MFPT) matrices for all 23 chromosomes under annealing conditions (high *β*) characteristic for each chromosome. (PDF)

S9 Fig Illustration of the difference between the concepts of (A) soft partitioning and (B) hard partitioning.
(PDF)

S10 FigThe dependence of partitioning on the value of metastability index $\rho_{M}$ for chromosomes 1, 17, and 20.In each of the following cases, we plot the partitioning diagrams for a chromosome at all levels that have metastability index $\rho_{M}$ below a threshold value *ρ*_*c*_: (A) Chromosome 1, *ρ*_*c*_ = 0.5, (B) Chromosome 17, *ρ*_*c*_ = 0.5, (C) Chromosome 20, *ρ*_*c*_ = 0.5, (D) Chromosome 1, *ρ*_*c*_ = 0.8, (E) Chromosome 17, *ρ*_*c*_ = 0.8, (F) Chromosome 20, *ρ*_*c*_ = 0.8.(PDF)

S11 Fig **Comparison of the raw Hi-C data matrix with the result of Gaussian Filter (GF) preprocessing on (A-B) chromosome 1, (C-D) chromosome 17, and (E-F) chromosome 20.** The width parameter σ = 200kbp, truncated at 4σ, was used for the Gaussian kernel. The original resolution of Hi-C data was 50kbp resolution. The two columns represent (A, C, E) raw matrices and (B, D, F) GF-preprocessed matrices.
(PDF)

S12 Fig Plot of the product of epigenetic factor densities in pairs of partitions against the effective interaction strength of the pair. Each panel shows the factor enrichment levels for (A) euchromatin-euchromatin partition pairs, (B) heterochromatin-heterochromatin pairs, and (C) euchromatin-heterochromatin pairs. We defined euchromatic partitions (EC) as those with more than 80% of the partition having stain levels [61] G-negative or G-positive25, and heterochromatic partitions (HC) as those with more than 80% of the partition having stain levels G-positive50, G-positive75, or G-positive100. For each interaction pair, we computed the product of ChIP-Seq signal values for each of the transcription factors and histone modifications studied in this work, and plotted it against the effective interaction strength as a density plot. We show a log-log regression line for each case, stating the r-value and p-value in each case.
(PDF)

S13 Fig Comparison of partitioning schemes obtained on different Hi-C datasets with GM12878_primary. (A) GM12878_replicate, (B) IMR90, (C) HUVEC, (D) HMEC.
(PDF)

S14 Fig Plots of metastability index *ρ* as a function of hub set size *n*, for all chromosomes. (PDF)

S15 Fig Partitioning plots for chromosome 17 at hub set size *n* = 12, for *β* ≥ 4.0. In the regime of high *β*, partitioning boundaries tend to be stable with respect to changes in the value of *β*.
(PDF)

S16 Fig Major clusters for the whole-genome effective interaction network for other cell lines. (A) IMR90, (B) HUVEC.
(PDF)

S1 Table Values of thermal annealing parameter *β* and hub set sizes *n* for three levels of hierarchy. These were the values used for the GM12878_primary dataset by Rao *et al*. [23].
(DOCX)

S2 Table Listing of the 539 partitions used for the whole-genome description. Start and end positions of partitions are provided.
(XLSX)

S3 Table Significant inter-chromosomal interactions between the central part and the periphery of the major cluster of the whole-genome inter-chromosomal effective interactions. The color of cells denotes the effective interaction strength: red cells are scaffold-layer interactions, while orange cells are Layer 1 interactions.
(PDF)

S4 Table Epigenetic factor enrichment profiles for all inter-chromosomal, high-affinity partition pairs. Partition labels are color-coded by their positions along the chromosome: pericentromeric partitions are red, and telomeric partitions are yellow. Each row represents a high-affinity partition pair (C > 3), and the cells under the factor columns represent the lower Z-score of that factor among the two partitions, using a discrete grey scale. Black cells indicate both Z-scores are above 2.0, dark grey between 1.5 and 2, and light grey for both between 1.0 and 1.5.
(XLSX)

S5 Table Subsets of inter-chromosomal, high-affinity partition pairs presented in S4 Table. (A) Cases where both partitions are large (>2Mb) and have strong effective interactions at Layer 1 or above; (B) Cases where both partitions are enriched in at least one common factor, with Z-scores above 2.0. This is also the set of all inter-chromosomal partition pairs included in high-affinity subnetworks Fig 8 and S6 Fig. Partition labels are color-coded by their positions along the chromosome: pericentromeric partitions are red, and telomeric partitions are yellow. Cells under factor columns represent Z-scores of partition pairs, using the same discrete grey scale scheme as in S4 Table.
(PDF)

S6 Table Summary statistics for partitioning network analysis on Hi-C data. Hi-C interaction matrices were obtained by Rao *et al*. [23] (GEO accession GSE63525).
(DOCX)

## References

[1] Entrevan M, Schuettengruber B, Cavalli G. Regulation of Genome Architecture and Function by Polycomb Proteins. Trends in Cell Biology. 2016;xx:1–15. 10.1016/j.tcb.2016.04.009 PMID: 27198635. 27198635

[2] Rieder D, Trajanoski Z, McNally JG. Transcription factories. Frontiers in Genetics. 2012;3:221 10.3389/fgene.2012.00221 . 23109938 PMC3478587

[3] Godfrey JE, Eisenberg H. The flexibility of low molecular weight double-stranded dna as a function of length. II. Light scattering measurements and the estimation of persistence lengths from light scattering, sedimentation and viscosity. Biophysical Chemistry. 1976;5:301–18. 10.1016/0301-4622(76)80042-7 . 987812

[4] Marmur J, Doty P. Determination of the base composition of deoxyribonucleic acid from its thermal denaturation temperature. Journal of Molecular Biology. 1962;5:109–18. 10.1016/S0022-2836(62)80066-7 . 14470099

[5] Tseitlin PI, Spitkovskii DM, Riabchenko NP. [On the relation of molecular morphology of DNA macromolecules to their radio-sensitivity. (On the problem of radio-sensitive and radio-resistant forms of DNA)]. Biofizika. 1960;5:393–7. . 13778357

[6] Spitkovskii DM. [Effect of free and forced convention currents on some structural properties of oriented desoxyribonucleoproteins]. Biofizika. 1962;7:96–8. . 13915847

[7] Spitkovskii DM, Luchkina LA, Andrianov VT, Pisarevskii AN. [Principal specific features of condensed DNP-systems depending on the variation of ionic content, pH and temperature of the medium (state of DNA in the composition of the nucleoprotein complex)]. Biofizika. 1967;12:592–605. . 5622217

[8] Dekker J, Rippe K, Dekker M, Kleckner N. Capturing chromosome conformation. Science (New York, NY). 2002;295:1306–11. 10.1126/science.1067799 . 11847345

[9] Ghirlando R, Felsenfeld G. Chromatin structure outside and inside the nucleus. Biopolymers. 2013;99:225–32. 10.1002/bip.22157 . 23348669 PMC3557801

[10] Bassett A, Cooper S, Wu C, Travers A. The folding and unfolding of eukaryotic chromatin. Current Opinion in Genetics and Development. 2009;19:159–65. 10.1016/j.gde.2009.02.010 . 19346124

[11] Ricci MA, Manzo C, García-Parajo MF, Lakadamyali M, Cosma MP. Chromatin fibers are formed by heterogeneous groups of nucleosomes in vivo. Cell. 2015;160:1145–58. 10.1016/j.cell.2015.01.054 . 25768910

[12] Canals-Hamann AZ, das Neves R, Reittie JE, Iñiguez C, Soneji S, Enver T, et al A biophysical model for transcription factories. BMC Biophysics. 2013;6:2 10.1186/2046-1682-6-2 . 23394119 PMC3740778

[13] Hübner MR, Eckersley-Maslin MA, Spector DL. Chromatin organization and transcriptional regulation. Current Opinion in Genetics and Development. 2013;23:89–95. 10.1016/j.gde.2012.11.006 . 23270812 PMC3612554

[14] Lanzuolo C, Roure V, Dekker J, Bantignies F, Orlando V. Polycomb response elements mediate the formation of chromosome higher-order structures in the bithorax complex. Nature cell biology. 2007;9:1167–74. 10.1038/ncb1637 . 17828248

[15] Li L, Lyu X, Hou C, Takenaka N, Nguyen HQ, Ong CT, et al Widespread Rearrangement of 3D Chromatin Organization Underlies Polycomb-Mediated Stress-Induced Silencing. Molecular cell. 2015;58:216–31. 10.1016/j.molcel.2015.02.023 . 25818644 PMC4402144

[16] Pombo A, Dillon N. Three-dimensional genome architecture: players and mechanisms. Nature reviews Molecular cell biology. 2015;16:245–57. 10.1038/nrm3965 . 25757416

[17] Sutherland H, Bickmore Wa. Transcription factories: gene expression in unions? Nature Reviews Genetics. 2009;10:457–66. 10.1038/nrg2592 . 19506577

[18] van Koningsbruggen S, Gierlinski M, Schofield P, Martin D, Barton GJ, Ariyurek Y, et al High-Resolution Whole-Genome Sequencing Reveals That Specific Chromatin Domains from Most Human Chromosomes Associate with Nucleoli. Molecular biology of the cell. 2010;21:3735–48. 10.1091/mbc.E10-06-0508 . 20826608 PMC2965689

[19] Edelman LB, Fraser P. Transcription factories: Genetic programming in three dimensions. Current Opinion in Genetics and Development. 2012;22:110–4. 10.1016/j.gde.2012.01.010 . 22365496

[20] Simonis M, Klous P, Splinter E, Moshkin Y, Willemsen R, de Wit E, et al Nuclear organization of active and inactive chromatin domains uncovered by chromosome conformation capture-on-chip (4C). Nature genetics. 2006;38:1348–54. 10.1038/ng1896 . 17033623

[21] Dostie J, Richmond TA, Arnaout RA, Selzer RR, Lee WL, Honan TA, et al Chromosome Conformation Capture Carbon Copy (5C): A massively parallel solution for mapping interactions between genomic elements. Genome research. 2006;16:1299–309. 10.1101/gr.5571506 . 16954542 PMC1581439

[22] Lieberman-Aiden E, van Berkum NL, Williams L, Imakaev M, Ragoczy T, Telling A, et al Comprehensive mapping of long-range interactions reveals folding principles of the human genome. Science (New York, NY). 2009;326:289–93. 10.1126/science.1181369 . 19815776 PMC2858594

[23] Rao SSP, Huntley MH, Durand NC, Stamenova EK, Bochkov ID, Robinson JT, et al A 3D Map of the Human Genome at Kilobase Resolution Reveals Principles of Chromatin Looping. Cell. 2014;159:1665–80. 10.1016/j.cell.2014.11.021 . 25497547 PMC5635824

[24] Imakaev MV, Fudenberg G, Mirny LA. Modeling chromosomes: Beyond pretty pictures. FEBS Letters. 2015;589:3031–6. 10.1016/j.febslet.2015.09.004 . 26364723 PMC4722799

[25] Grosberg A, Rabin Y, Havlin S, Neer A. Crumpled Globule Model of the Three-Dimensional Structure of DNA. Europhysics Letters. 1993;23:373–8. 10.1209/0295-5075/23/5/012

[26] Grosberg AY, Nechaev SK, Shakhnovich EI. The role of topological constraints in the kinetics of collapse of macromolecules. Journal de Physique. 1988;49:2095–100. 10.1051/jphys:0198800490120209500 PubMed PMID: 20650282.

[27] Rosa A, Everaers R. Structure and dynamics of interphase chromosomes. PLoS Computational Biology. 2008;4:e1000153 10.1371/journal.pcbi.1000153 . 18725929 PMC2515109

[28] Barbieri M, Chotalia M, Fraser J, Lavitas LM, Dostie J, Pombo A, et al Complexity of chromatin folding is captured by the strings and binders switch model. Proceedings of the National Academy of Sciences of the United States of America. 2012;109:16173–8. 10.1073/pnas.1204799109 . 22988072 PMC3479593

[29] Fudenberg G, Imakaev M, Lu C, Goloborodko A, Abdennur N, Mirny LA. Formation of Chromosomal Domains by Loop Extrusion. Cell reports. 2016;15:2038–49. 10.1016/j.celrep.2016.04.085 . 27210764 PMC4889513

[30] Amitai A, Holcman D. Polymer physics of nuclear organization and function. Physics Reports. 2017;678:1–83. 10.1016/j.physrep.2017.02.002

[31] Brackley CA, Brown JM, Waithe D, Babbs C, Davies J, Hughes JR, et al Predicting the three-dimensional folding of cis-regulatory regions in mammalian genomes using bioinformatic data and polymer models. Genome biology. 2016;17:59 10.1186/s13059-016-0909-0 . 27036497 PMC4815170

[32] Di Pierro M, Cheng RR, Lieberman Aiden E, Wolynes PG, Onuchic JN. De novo prediction of human chromosome structures: Epigenetic marking patterns encode genome architecture. Proceedings of the National Academy of Sciences. 2017;114:12126–31. 10.1073/pnas.1714980114 . 29087948 PMC5699090

[33] Jost D, Carrivain P, Cavalli G, Vaillant C. Modeling epigenome folding: Formation and dynamics of topologically associated chromatin domains. Nucleic acids research. 2014;42:9553–61. 10.1093/nar/gku698 . 25092923 PMC4150797

[34] Jost D, Vaillant C, Meister P. Coupling 1D modifications and 3D nuclear organization: data, models and function. Current Opinion in Cell Biology. 2017;44:20–7. 10.1016/j.ceb.2016.12.001 . 28040646

[35] Dixon JR, Selvaraj S, Yue F, Kim A, Li Y, Shen Y, et al Topological domains in mammalian genomes identified by analysis of chromatin interactions. Nature. 2012;485:376–80. 10.1038/nature11082 . 22495300 PMC3356448

[36] Fortin JP, Hansen KD. Reconstructing A/B compartments as revealed by Hi-C using long-range correlations in epigenetic data. Genome biology. 2015;16:180 Epub 2015/09/01. 10.1186/s13059-015-0741-y 26316348 PMC4574526

[37] Boulos RE, Tremblay N, Arneodo A, Borgnat P, Audit B. Multi-scale structural community organisation of the human genome. BMC Bioinformatics. 2017;18:209 10.1186/s12859-017-1616-x 28399820 PMC5387268

[38] Fraser J, Ferrai C, Chiariello AM, Schueler M, Rito T, Laudanno G, et al Hierarchical folding and reorganization of chromosomes are linked to transcriptional changes in cellular differentiation. Molecular Systems Biology. 2015;11:852–. 10.15252/msb.20156492 . 26700852 PMC4704492

[39] Haddad N, Vaillant C, Jost D. IC-Finder: Inferring robustly the hierarchical organization of chromatin folding. Nucleic acids research. 2017;45:e81 10.1093/nar/gkx036 . 28130423 PMC5449546

[40] Zhan Y, Mariani L, Barozzi I, Schulz EG, Blüthgen N, Stadler M, et al Reciprocal insulation analysis of Hi-C data shows that TADs represent a functionally but not structurally privileged scale in the hierarchical folding of chromosomes. Genome research. 2017;27:479–90. 10.1101/gr.212803.116 . 28057745 PMC5340975

[41] Belyaeva A, Venkatachalapathy S, Nagarajan M, Shivashankar GV, Uhler C. Network analysis identifies chromosome intermingling regions as regulatory hotspots for transcription. Proceedings of the National Academy of Sciences. 2017;114:13714–9. 10.1073/pnas.1708028115 . 29229825 PMC5748172

[42] Sandhu KS, Li G, Poh HM, Quek YLK, Sia YY, Peh SQ, et al Large-Scale Functional Organization of Long-Range Chromatin Interaction Networks. Cell reports. 2012;2:1207–19. 10.1016/j.celrep.2012.09.022 . 23103170 PMC4181841

[43] Thibodeau A, Márquez EJ, Shin DG, Vera-Licona P, Ucar D. Chromatin interaction networks revealed unique connectivity patterns of broad H3K4me3 domains and super enhancers in 3D chromatin. Scientific Reports. 2017;7:14466 10.1038/s41598-017-14389-7 . 29089515 PMC5663946

[44] Pancaldi V, Carrillo-de-Santa-Pau E, Javierre BM, Juan D, Fraser P, Spivakov M, et al Integrating epigenomic data and 3D genomic structure with a new measure of chromatin assortativity. Genome biology. 2016;17(1):152 Epub 2016/07/09. 10.1186/s13059-016-1003-3 27391817 PMC4939006

[45] Crane E, Bian Q, McCord RP, Lajoie BR, Wheeler BS, Ralston EJ, et al Condensin-driven remodelling of X chromosome topology during dosage compensation. Nature. 2015;523:240–4. 10.1038/nature14450 . 26030525 PMC4498965

[46] Filippova D, Patro R, Duggal G, Kingsford C. Multiscale identification of topological domains in chromatin. Lecture Notes in Computer Science (including subseries Lecture Notes in Artificial Intelligence and Lecture Notes in Bioinformatics). 2013;8126 LNBI:300–12. 10.1007/978-3-642-40453-5_23 PubMed PMID: 24868242.

[47] Lévy-Leduc C, Delattre M, Mary-Huard T, Robin S. Two-dimensional segmentation for analyzing Hi-C data. Bioinformatics. 2014;30:i386—92. 10.1093/bioinformatics/btu443 . 25161224 PMC4147896

[48] Kruse K, Hug CB, Hernandez-Rodriguez B, Vaquerizas JM. TADtool: Visual parameter identification for TAD-calling algorithms. Bioinformatics. 2016;32:3190–2. 10.1093/bioinformatics/btw368 . 27318199 PMC5048066

[49] Forcato M, Nicoletti C, Pal K, Livi CM, Ferrari F, Bicciato S. Comparison of computational methods for Hi-C data analysis. Nature methods. 2017;14:679–85. 10.1038/nmeth.4325 . 28604721 PMC5493985

[50] Sauerwald N, Zhang S, Kingsford C, Bahar I. Chromosomal dynamics predicted by an elastic network model explains genome-wide accessibility and long-range couplings. Nucleic acids research. 2017;45(7):3663–73. Epub 2017/03/24. 10.1093/nar/gkx172 28334818 PMC5397156

[51] Duan Z, Andronescu M, Schutz K, McIlwain S, Kim YJ, Lee C, et al A three-dimensional model of the yeast genome. Nature. 2010;465:363–7. 10.1038/nature08973 . 20436457 PMC2874121

[52] Lesne A, Riposo J, Roger P, Cournac A, Mozziconacci J. 3D genome reconstruction from chromosomal contacts. Nature methods. 2014;11:1141–3. 10.1038/nmeth.3104 . 25240436

[53] Serra F, Baù D, Goodstadt M, Castillo D, Filion G, Marti-Renom MA. Automatic analysis and 3D-modelling of Hi-C data using TADbit reveals structural features of the fly chromatin colors. PLoS Computational Biology. 2017;13:e1005665 10.1371/journal.pcbi.1005665 . 28723903 PMC5540598

[54] Zhang B, Wolynes PG. Topology, structures, and energy landscapes of human chromosomes. Proceedings of the National Academy of Sciences. 2015;112:6062–7. 10.1073/pnas.1506257112 25918364 PMC4434716

[55] Kalhor R, Tjong H, Jayathilaka N, Alber F, Chen L. Genome architectures revealed by tethered chromosome conformation capture and population-based modeling. Nature biotechnology. 2012;30:90–8. 10.1038/nbt.2057 . 22198700 PMC3782096

[56] E W, Li T, Vanden-Eijnden E. Optimal partition and effective dynamics of complex networks. Proceedings of the National Academy of Sciences of the United States of America. 2008;105(23):7907–12. Epub 2008/02/28. 10.1073/pnas.0707563105 18303119 PMC2786939

[57] Lafon S, Lee AB. Diffusion maps and coarse-graining: A unified framework for dimensionality reduction, graph partitioning, and data set parameterization. IEEE transactions on pattern analysis and machine intelligence. 2006;28(9):1393–403. Epub 2006/08/26. 10.1109/TPAMI.2006.184 . 16929727

[58] Rosvall M, Bergstrom CT. Maps of random walks on complex networks reveal community structure. Proceedings of the National Academy of Sciences of the United States of America. 2008;105(4):1118–23. Epub 2008/01/25. 10.1073/pnas.0706851105 18216267 PMC2234100

[59] Guarnera E, Vanden-Eijnden E. Optimized Markov state models for metastable systems. Journal of Chemical Physics. 2016;145:024102 10.1063/1.4954769 27421392

[60] Bastian M, Heymann S, Jacomy M. Gephi: an open source software for exploring and manipulating networks. International AAAI Conference on Weblogs and Social Media. 2009;8(2009):361–2.

[61] BAC Resource Consortium T, Cheung VG, Nowak N, Jang W, Kirsch IR, Zhao S, et al Integration of cytogenetic landmarks into the draft sequence of the human genome. Nature. 2001;409:953–8. 10.1038/35057192 11237021 PMC7845515

[62] Niimura Y, Gojobori T. In silico chromosome staining: reconstruction of Giemsa bands from the whole human genome sequence. Proceedings of the National Academy of Sciences of the United States of America. 2002;99:797–802. 10.1073/pnas.022437999 . 11792839 PMC117385

[63] Parelho V, Hadjur S, Spivakov M, Leleu M, Sauer S, Gregson HC, et al Cohesins Functionally Associate with CTCF on Mammalian Chromosome Arms. Cell. 2008;132:422–33. 10.1016/j.cell.2008.01.011 . 18237772

[64] Rubio ED, Reiss DJ, Welcsh PL, Disteche CM, Filippova GN, Baliga NS, et al CTCF physically links cohesin to chromatin. Proceedings of the National Academy of Sciences of the United States of America. 2008;105:8309–14. 10.1073/pnas.0801273105 . 18550811 PMC2448833

[65] Vietri Rudan M, Barrington C, Henderson S, Ernst C, Odom DT, Tanay A, et al Comparative Hi-C reveals that CTCF underlies evolution of chromosomal domain architecture. Cell reports. 2015;10(8):1297–309. Epub 2015/03/04. 10.1016/j.celrep.2015.02.004 25732821 PMC4542312

[66] Pirrotta V, Li HB. A view of nuclear Polycomb bodies. Current Opinion in Genetics and Development. 2012;22:101–9. 10.1016/j.gde.2011.11.004 . 22178420 PMC3329586

[67] Katainen R, Dave K, Pitkanen E, Palin K, Kivioja T, Valimaki N, et al CTCF/cohesin-binding sites are frequently mutated in cancer. Nature genetics. 2015;47(7):818–21. Epub 2015/06/09. 10.1038/ng.3335 . 26053496

[68] Zuin J, Dixon JR, van der Reijden MI, Ye Z, Kolovos P, Brouwer RW, et al Cohesin and CTCF differentially affect chromatin architecture and gene expression in human cells. Proceedings of the National Academy of Sciences of the United States of America. 2014;111(3):996–1001. Epub 2013/12/18. 10.1073/pnas.1317788111 24335803 PMC3903193

[69] Curtiss M, Jones C, Babst M. Efficient cargo sorting by ESCRT-I and the subsequent release of ESCRT-I from multivesicular bodies requires the subunit Mvb12. Molecular biology of the cell. 2007;18(2):636–45. Epub 2006/12/01. 10.1091/mbc.E06-07-0588 17135292 PMC1783790

[70] Wu L, Chang W, Zhao J, Yu Y, Tan X, Su T, et al Development of autoantibody signatures as novel diagnostic biomarkers of non-small cell lung cancer. Clinical cancer research: an official journal of the American Association for Cancer Research. 2010;16(14):3760–8. Epub 2010/05/27. 10.1158/1078-0432.ccr-10-0193 . 20501620

[71] Wang Y, Fan C, Zheng Y, Li C. Dynamic chromatin accessibility modeled by Markov process of randomly-moving molecules in the 3D genome. Nucleic Acids Res. 2017. papers3://publication/doi/ 10.1093/nar/gkx086 PMC5449544 28180283

[72] Hansen AS, Cattoglio C, Darzacq X, Tjian R. Recent evidence that TADs and chromatin loops are dynamic structures. Nucleus (Austin, Tex). 2018;9(1):20–32. Epub 2017/10/28. 10.1080/19491034.2017.1389365 29077530 PMC5990973

[73] Nagano T, Lubling Y, Stevens TJ, Schoenfelder S, Yaffe E, Dean W, et al Single-cell Hi-C reveals cell-to-cell variability in chromosome structure. Nature. 2013;502(7469):59–64. Epub 2013/09/27. 10.1038/nature12593 24067610 PMC3869051

[74] Jager R, Migliorini G, Henrion M, Kandaswamy R, Speedy HE, Heindl A, et al Capture Hi-C identifies the chromatin interactome of colorectal cancer risk loci. Nature communications. 2015;6:6178 Epub 2015/02/20. 10.1038/ncomms7178 25695508 PMC4346635

[75] Wei CL, Wu Q, Vega VB, Chiu KP, Ng P, Zhang T, et al A global map of p53 transcription-factor binding sites in the human genome. Cell. 2006;124(1):207–19. Epub 2006/01/18. 10.1016/j.cell.2005.10.043 . 16413492

[76] Mumbach MR, Rubin AJ, Flynn RA, Dai C, Khavari PA, Greenleaf WJ, et al HiChIP: efficient and sensitive analysis of protein-directed genome architecture. Nature methods. 2016;13(11):919–22. Epub 2016/11/01. 10.1038/nmeth.3999 27643841 PMC5501173

[77] Beagrie RA, Scialdone A, Schueler M, Kraemer DC, Chotalia M, Xie SQ, et al Complex multi-enhancer contacts captured by genome architecture mapping. Nature. 2017;543(7646):519–24. Epub 2017/03/09. 10.1038/nature21411 28273065 PMC5366070

[78] Kemeny JG, Snell JL. Finite Markov Chains: With a New Appendix "Generalization of a Fundamental Matrix": Springer New York; 1983.

[79] Cline MS, Smoot M, Cerami E, Kuchinsky A, Landys N, Workman C, et al Integration of biological networks and gene expression data using cytoscape. Nature Protocols. 2007;2:2366–82. 10.1038/nprot.2007.324 . 17947979 PMC3685583

