# Supplementary figures and images for: Exploring chromatin hierarchical organization via Markov State Modelling
Source: PLoS Comput Biol. 2018 Dec 31;14(12):e1006686. doi: 10.1371/journal.pcbi.1006686 (PMC6355033; doi:10.1371/journal.pcbi.1006686)

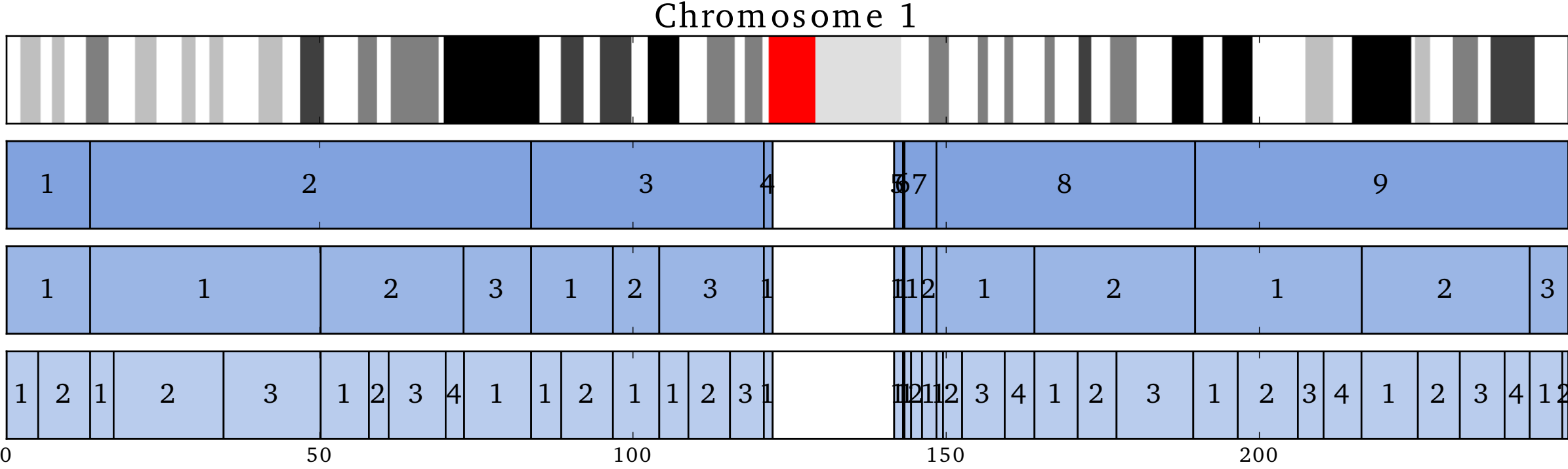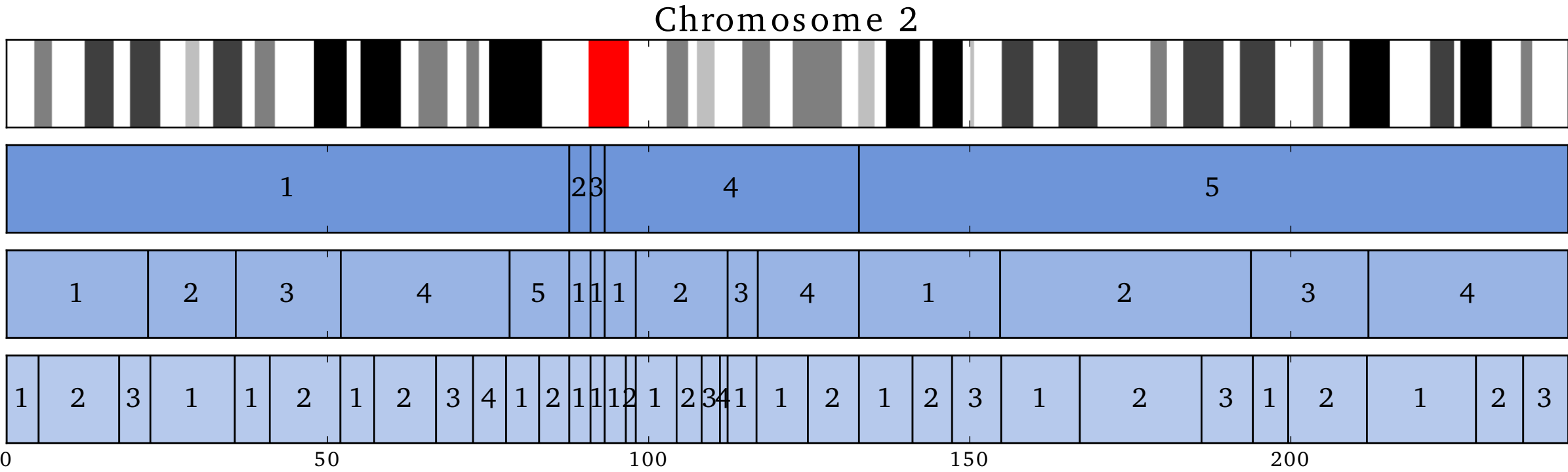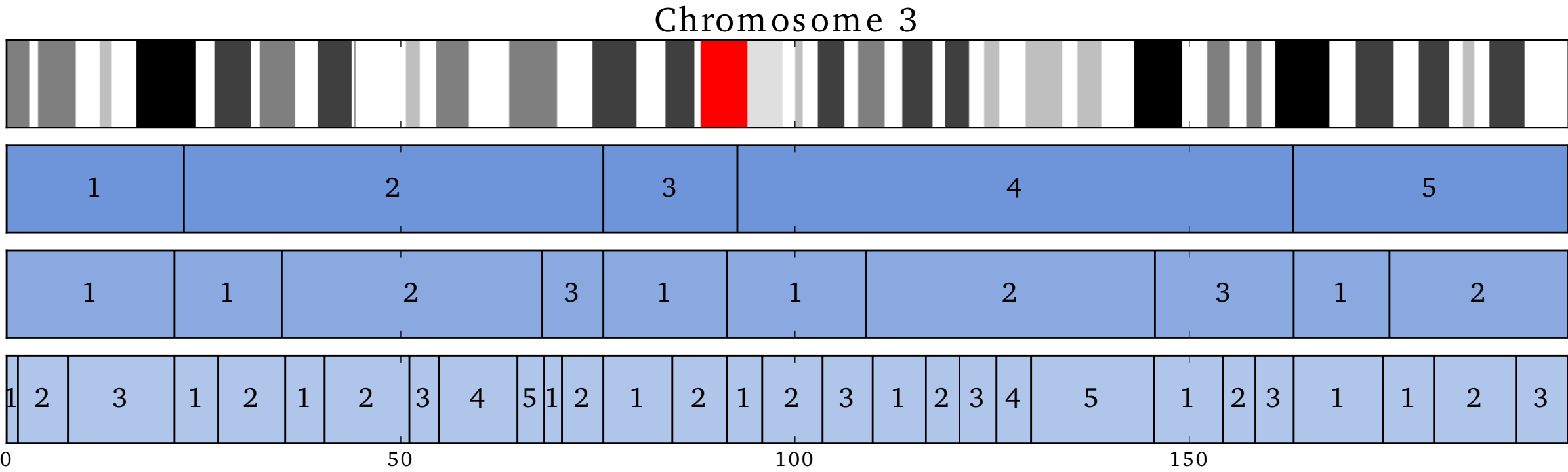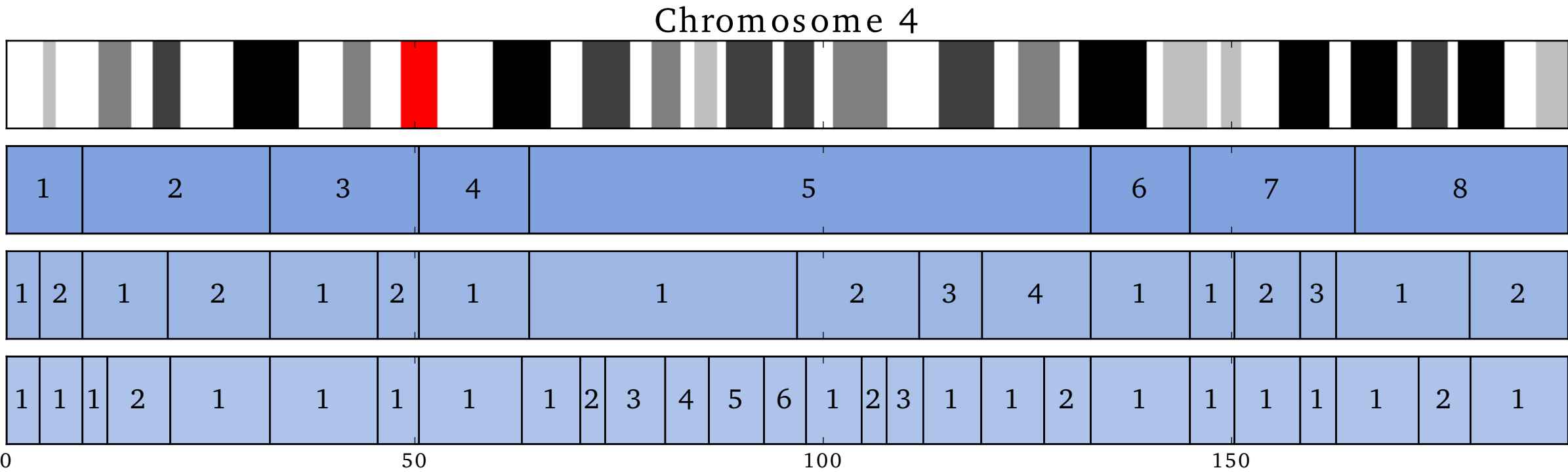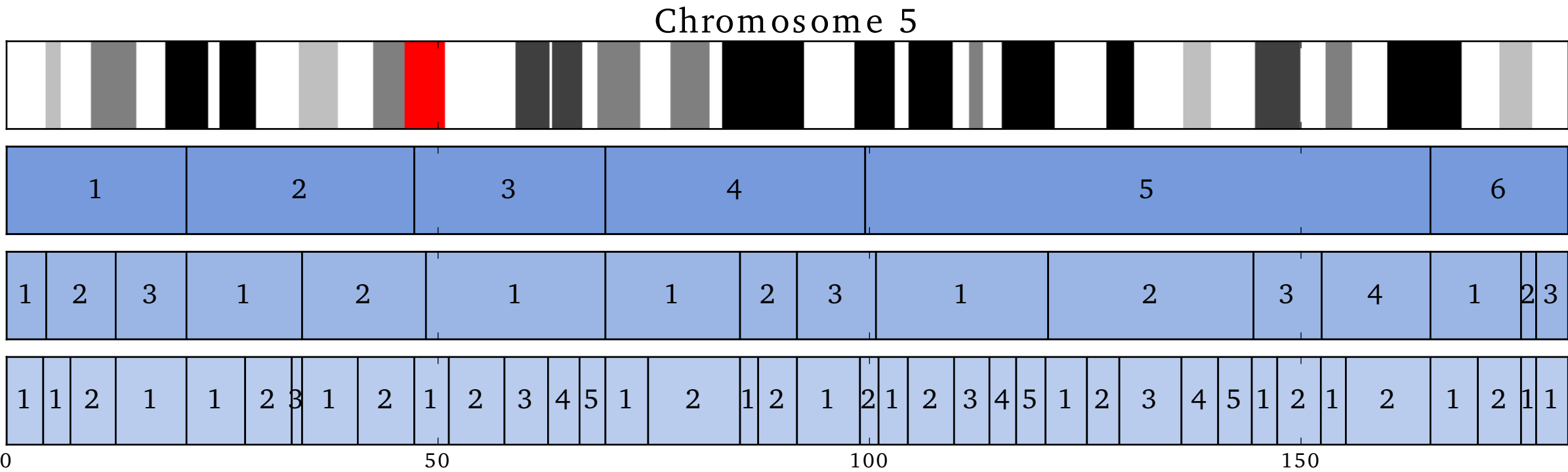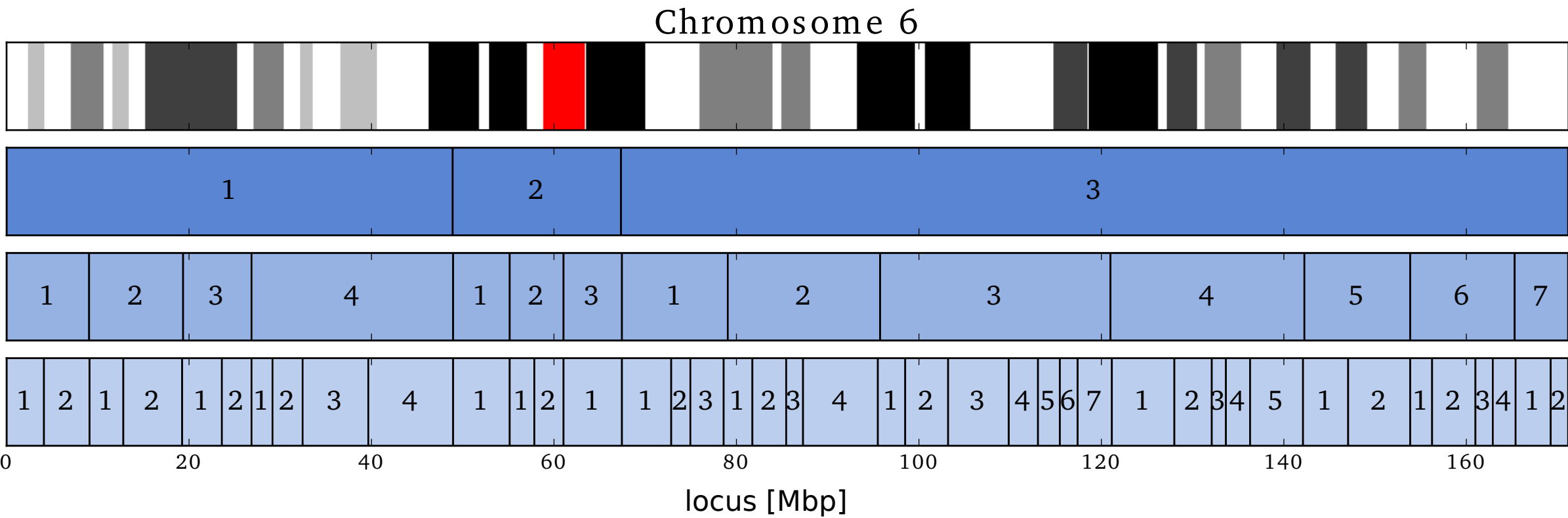

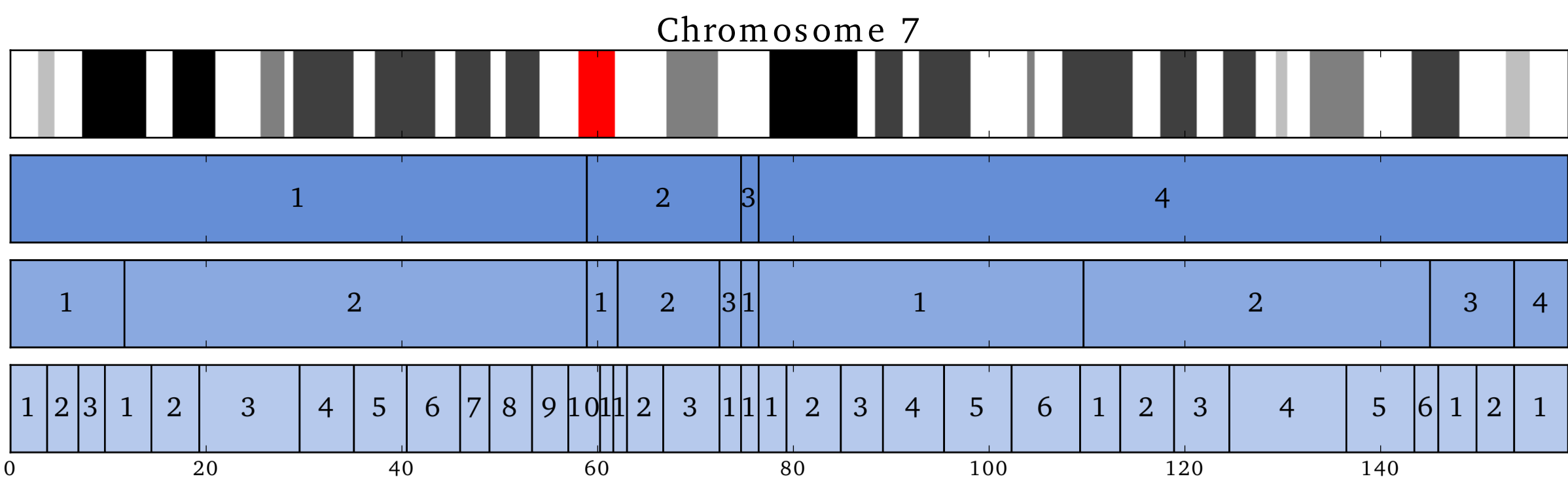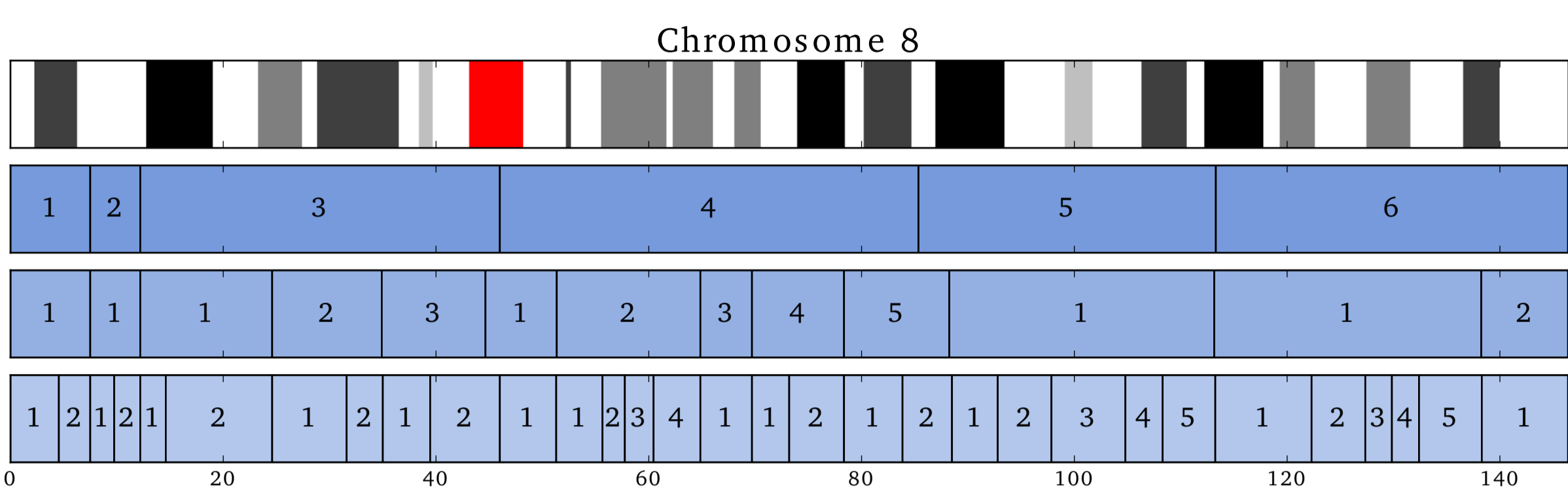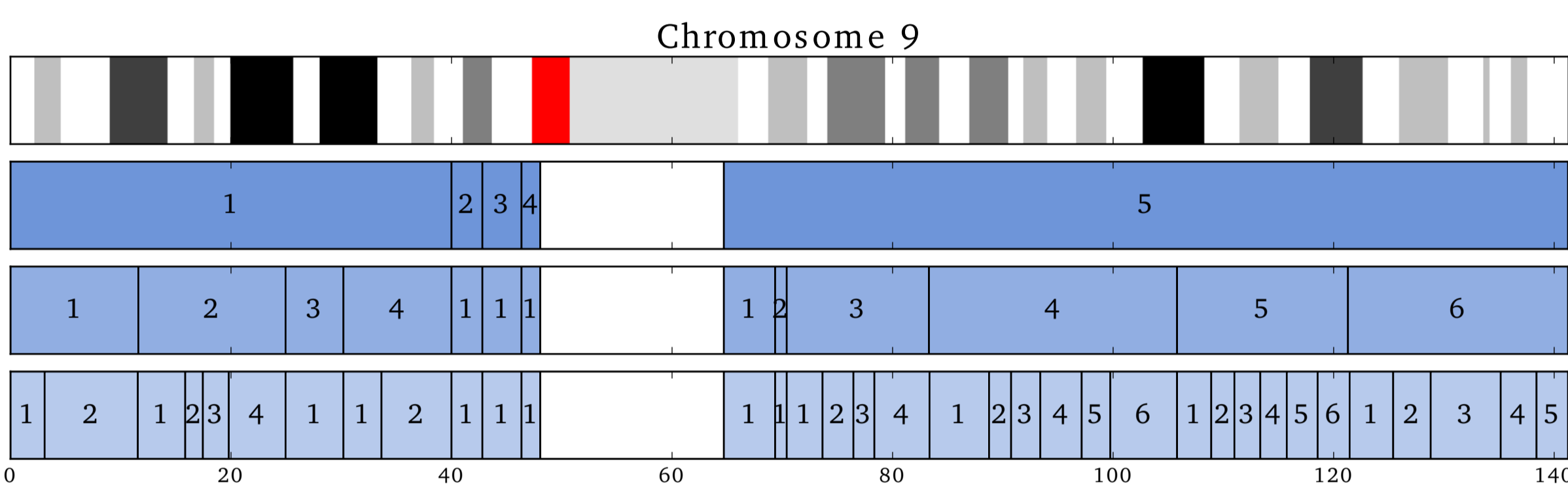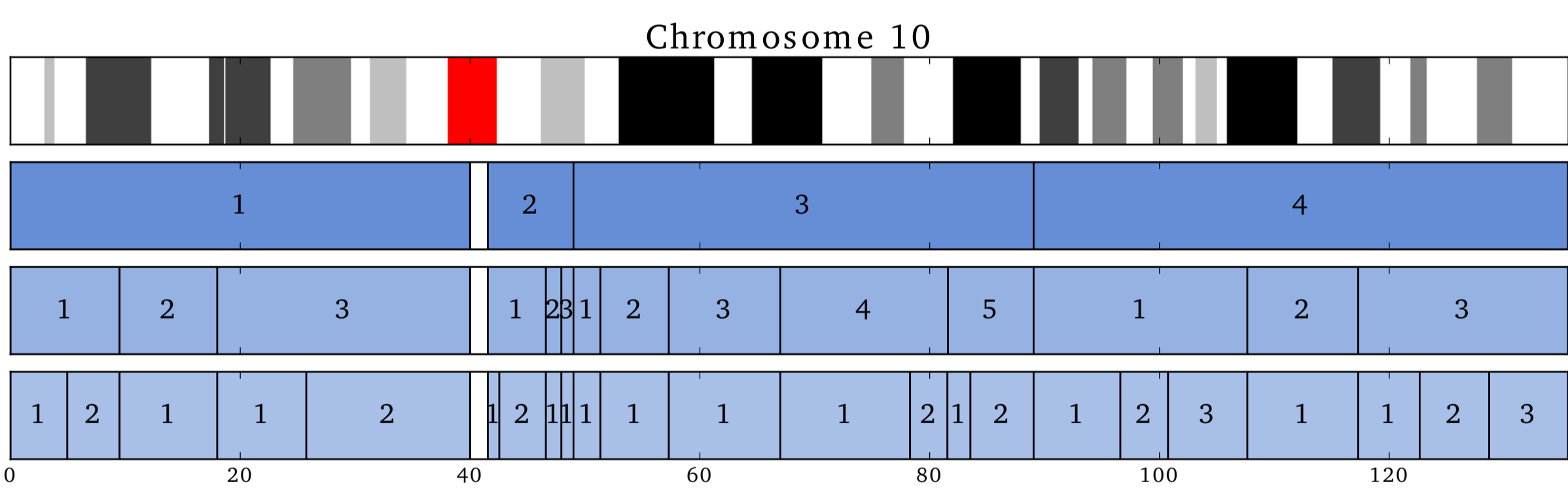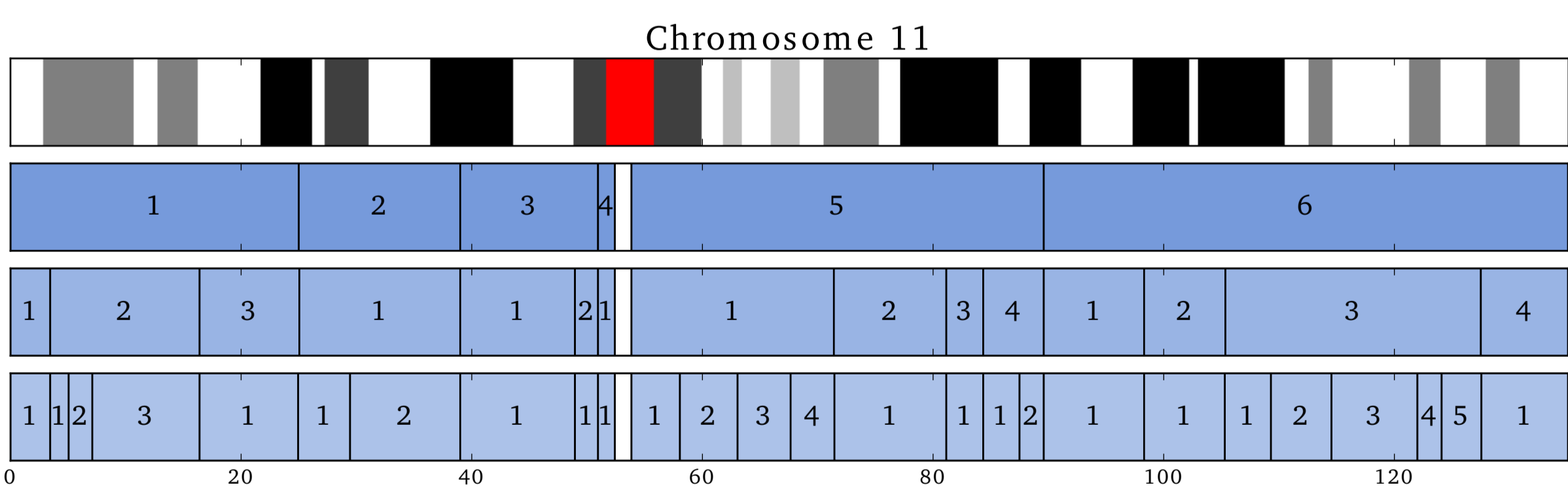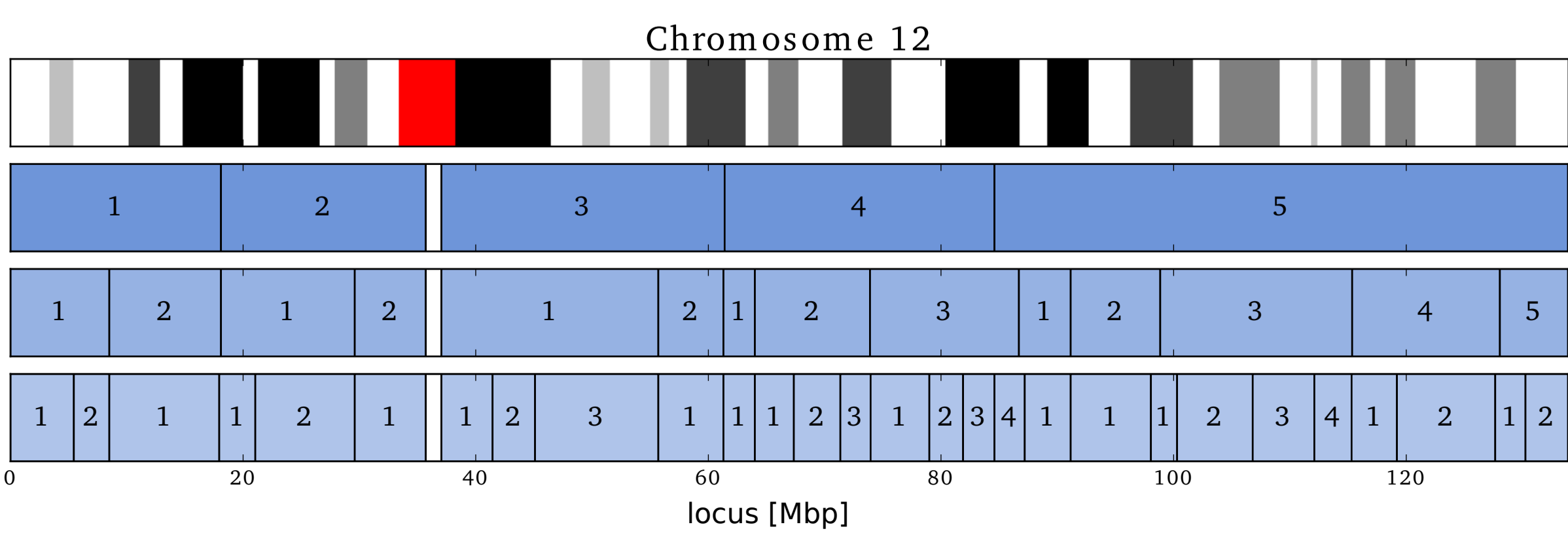

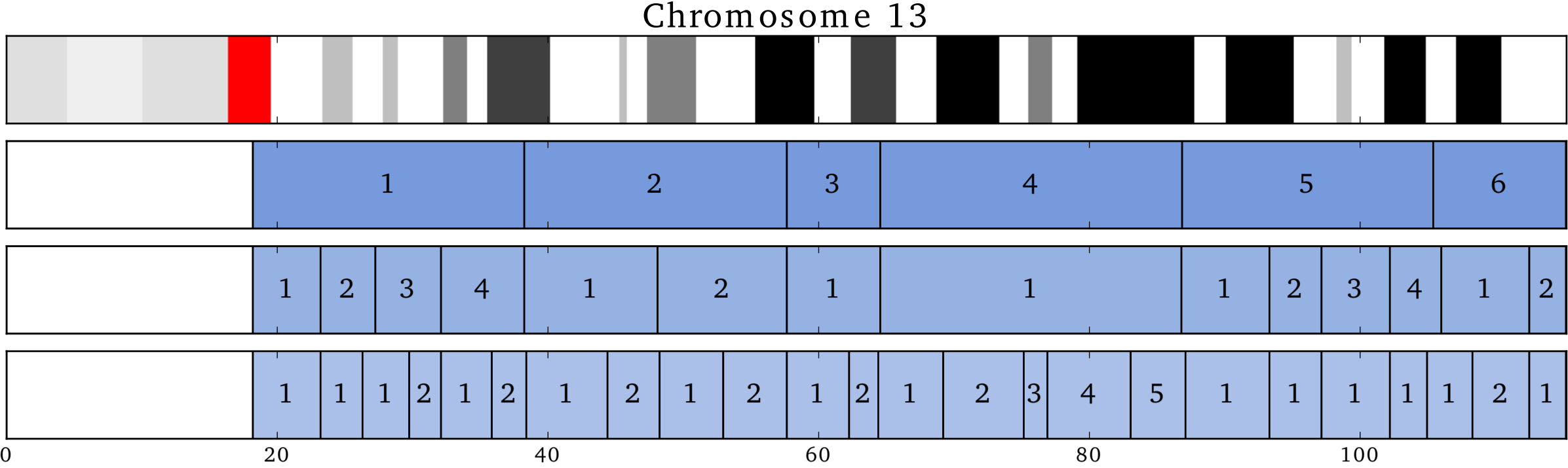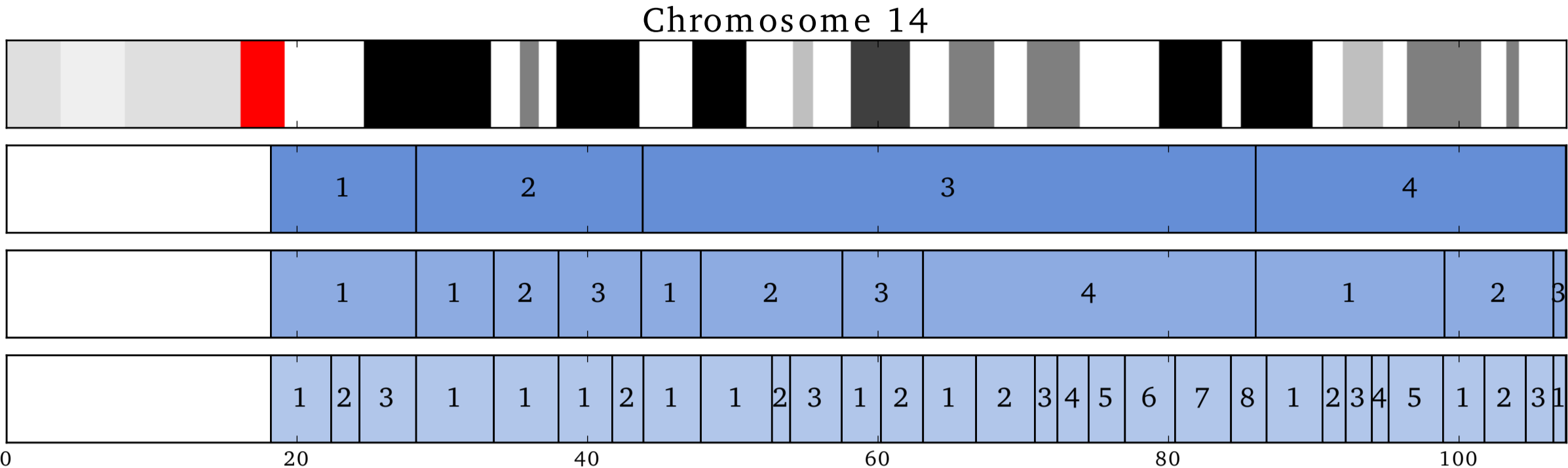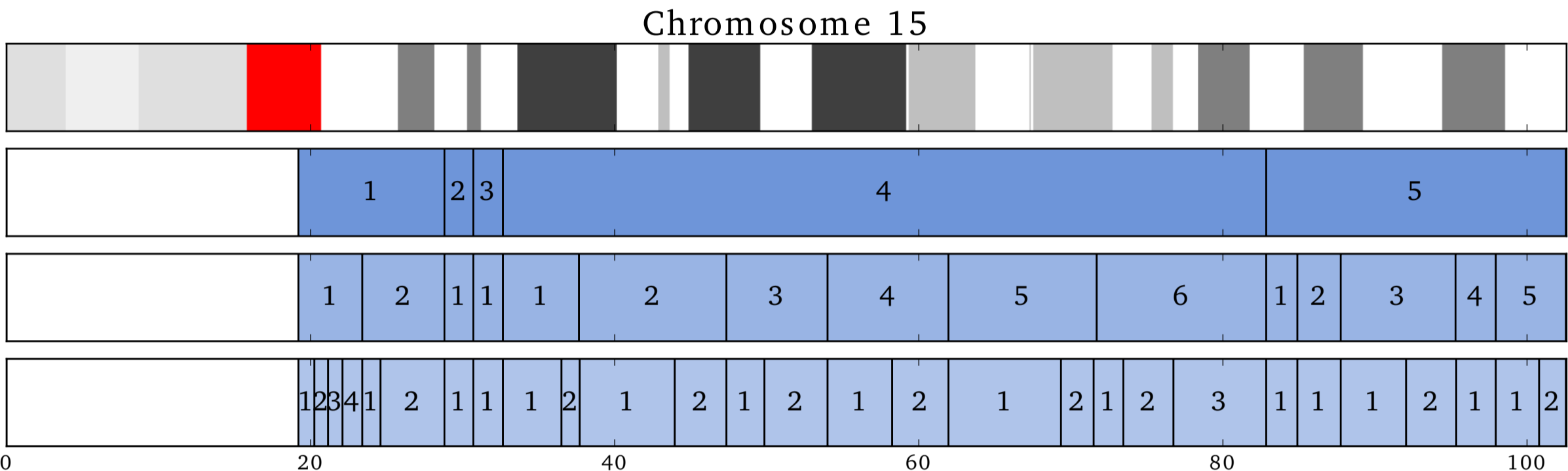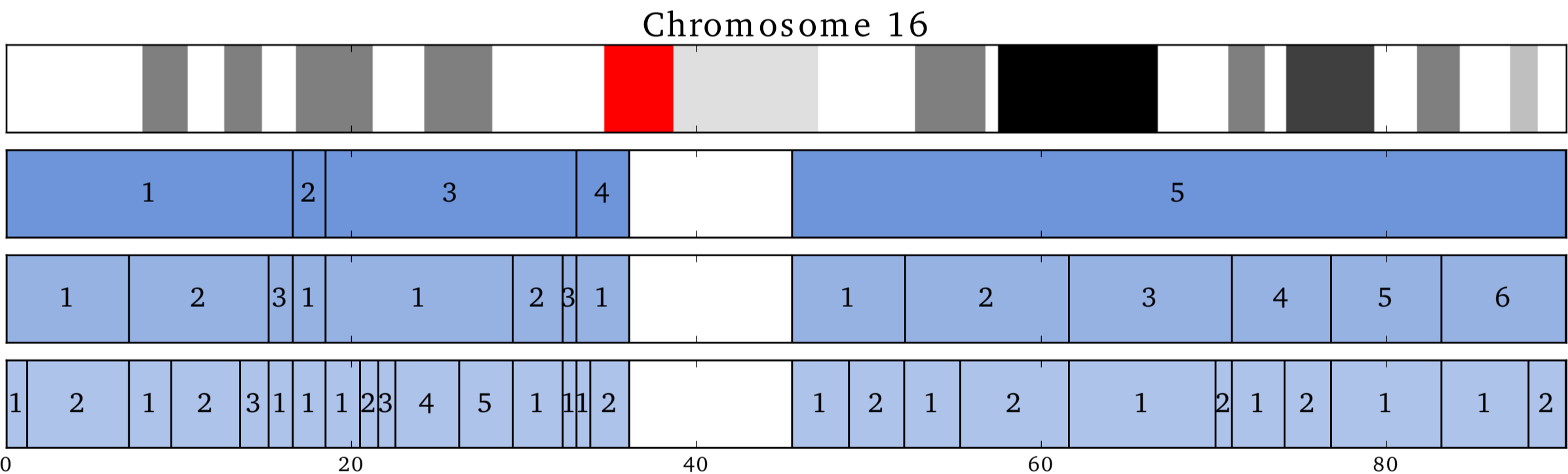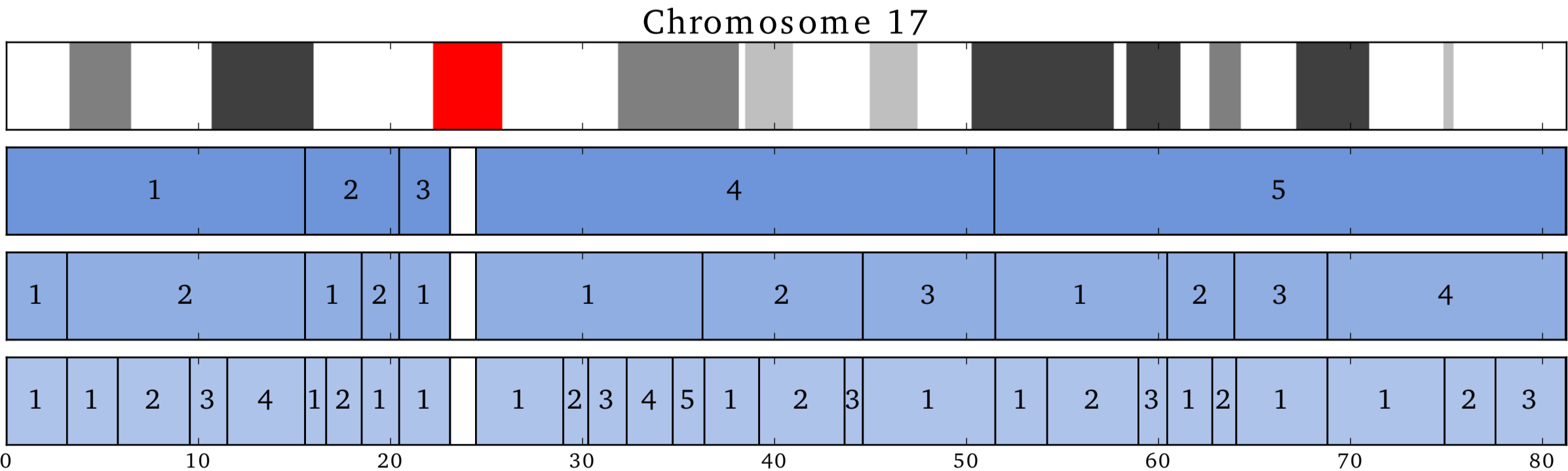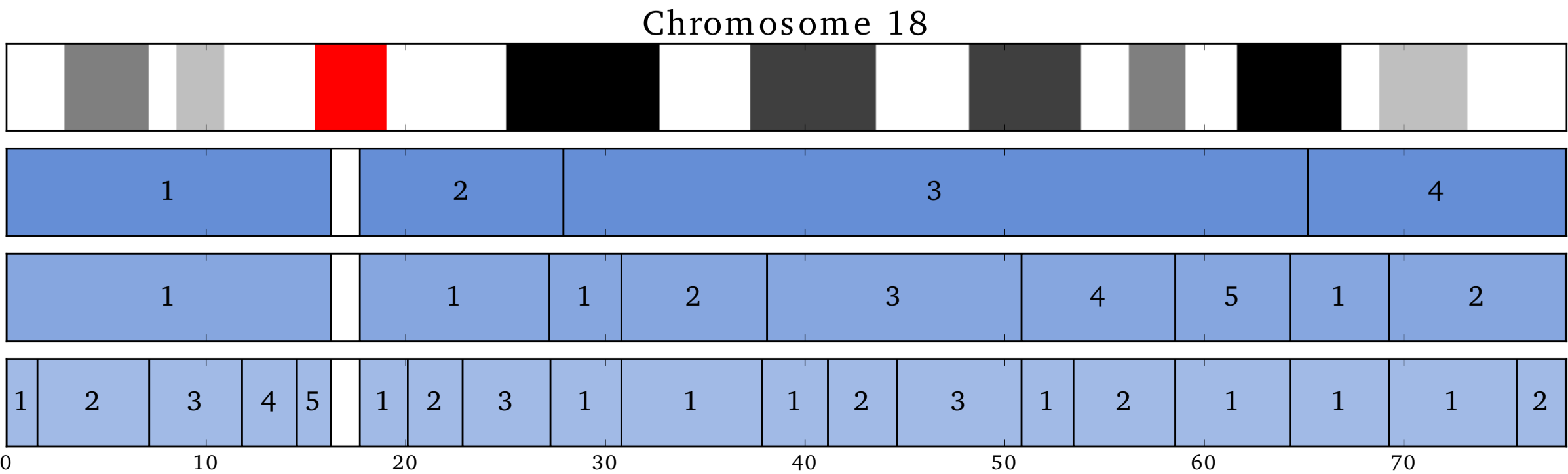

locus [Mbp]

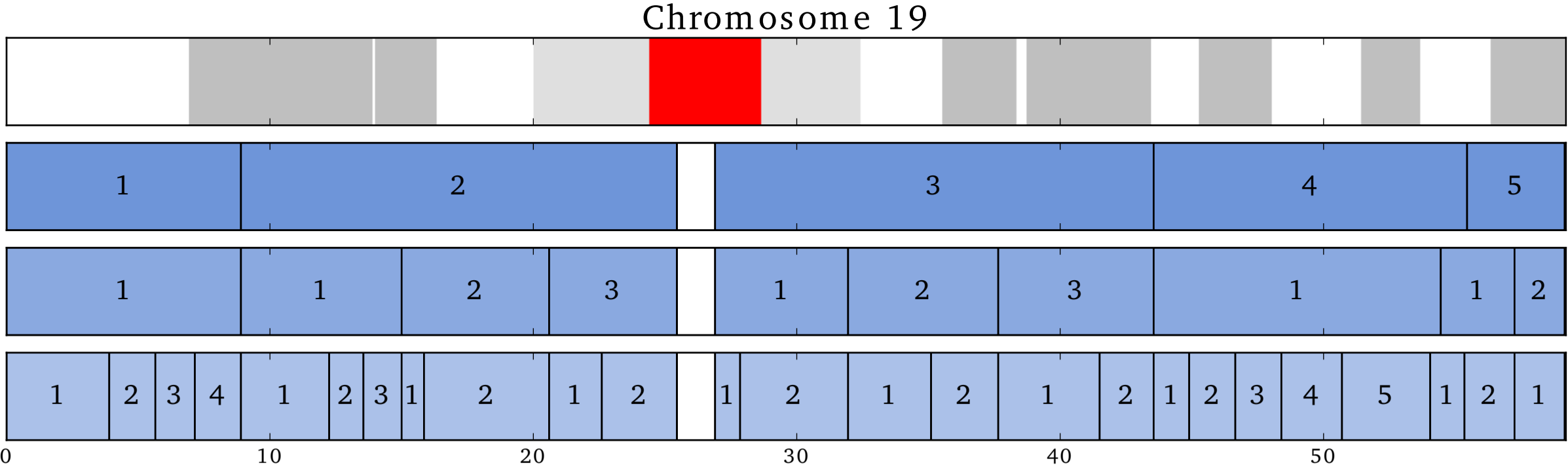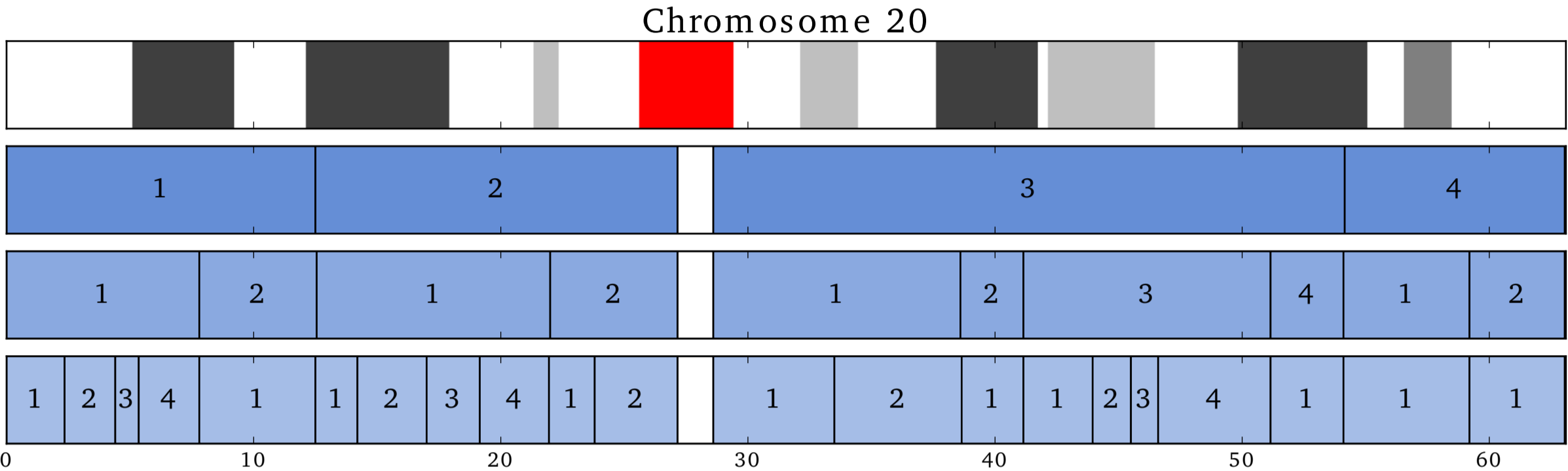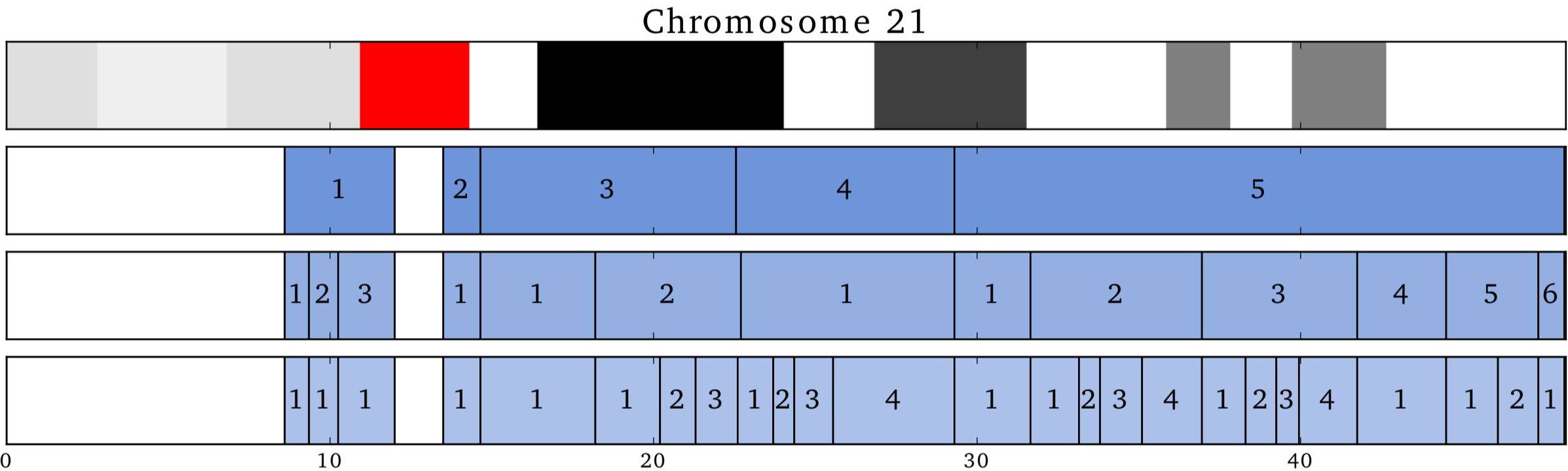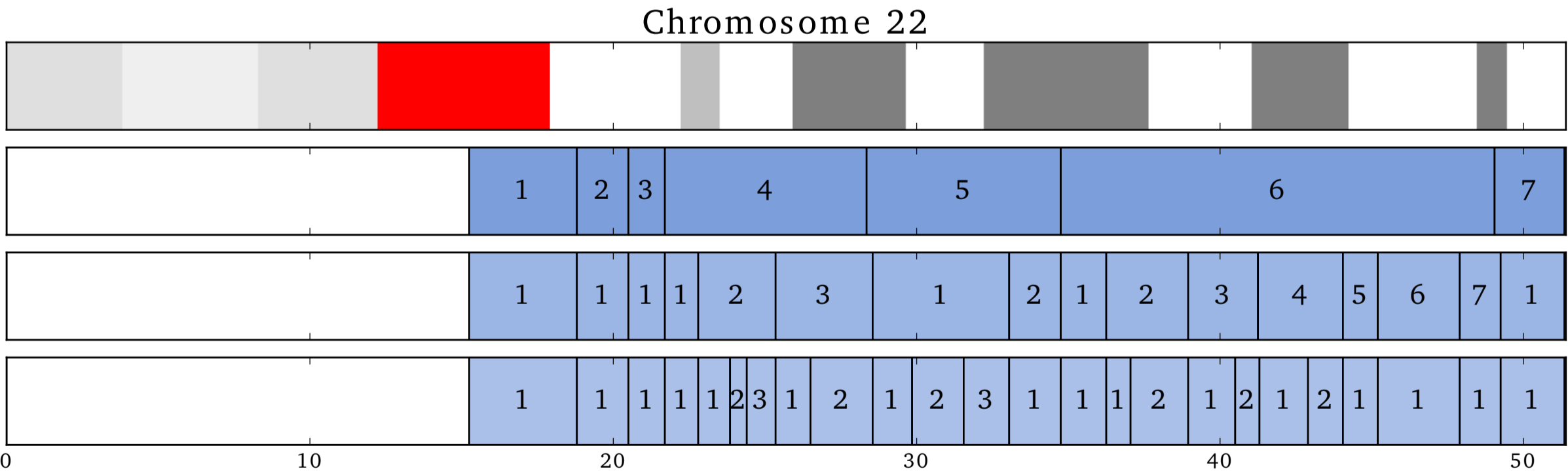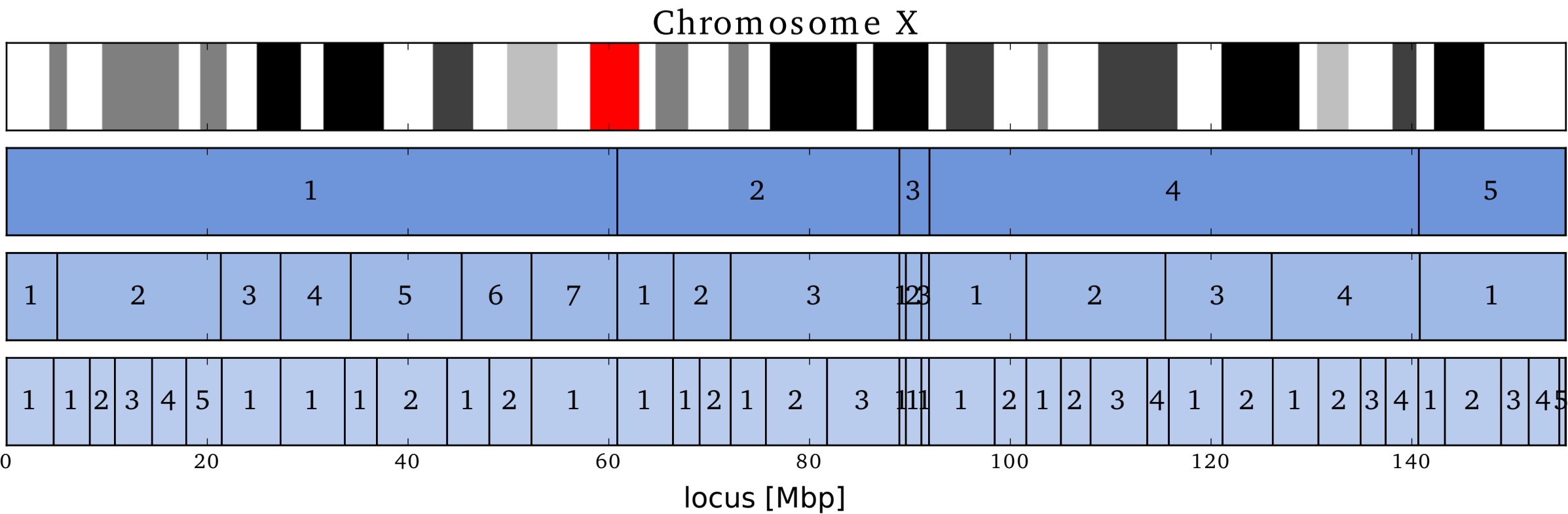

Supplement: S1 Fig — Three levels of hierarchy are presented and matched to the linear map of corresponding chromosomes with eu-/heterochromatic bands marked according to Giemsa staining [61]. (PDF) [file pcbi.1006686.s001.pdf]

Chromosome c

Chromosome c'

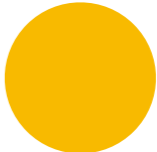

loci

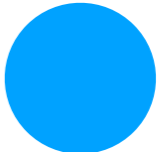

hub loci

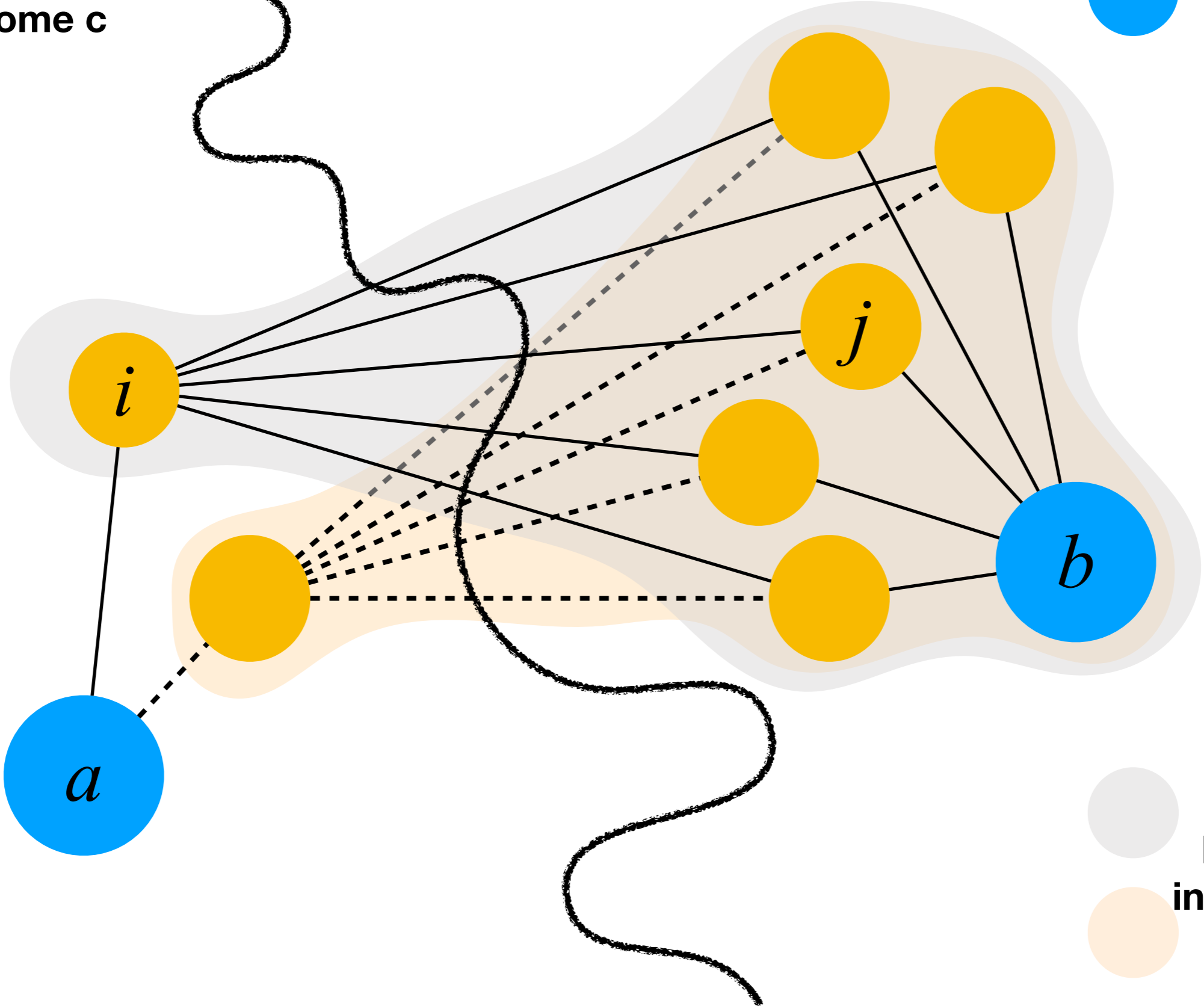

Supplement: S2 Fig — Given two hub loci a and b belonging to two different chromosomes, the effective interaction between them is estimated by summing up the fluxes between a and b passing through all possible pairs of intermediate loci i and j that belong to these two chromosomes. (PDF) [file pcbi.1006686.s002.pdf]

A

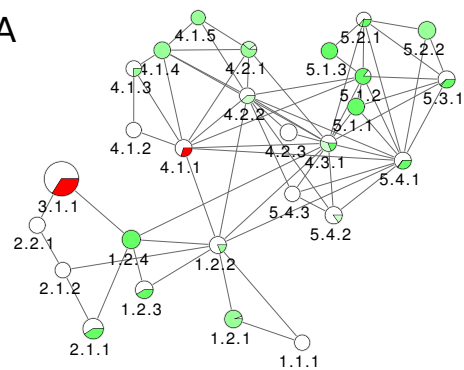

B

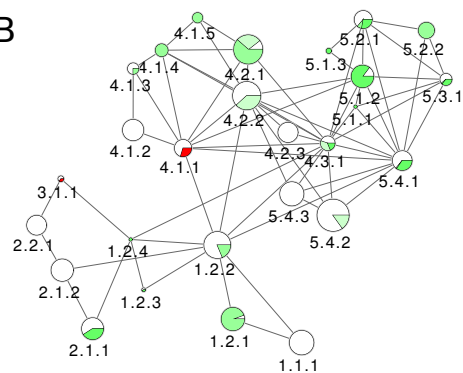

C

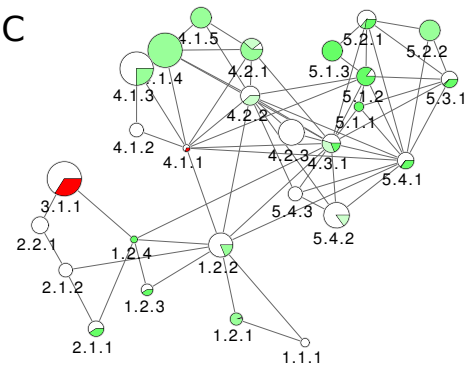

D

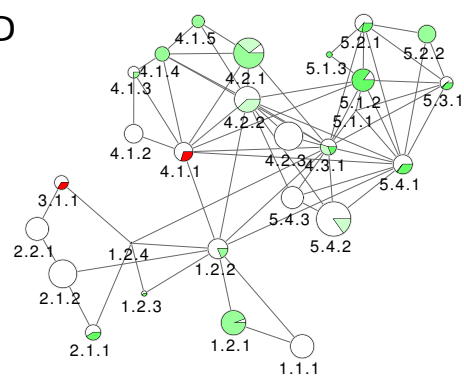

E

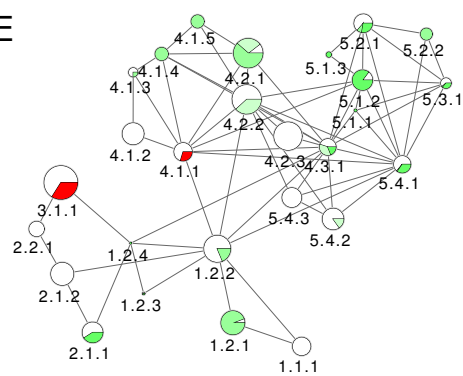

F

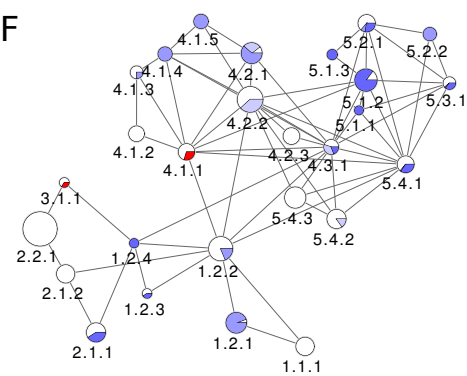

G

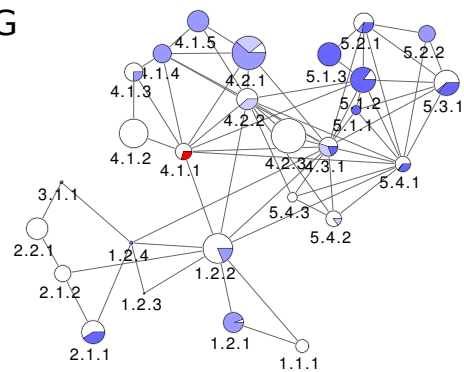

H

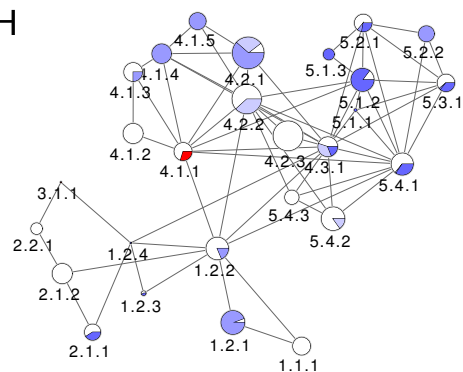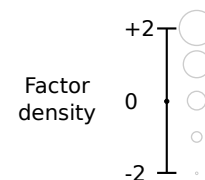

Supplement: S3 Fig — Edge widths correspond to effective interaction strengths, and node sizes in each panel represent Z scores for the following factors: (A) H3K9me3, (B) H3K27ac, (C) H3K27me3, (D) H3K4me1, (E) H3K4me3, (F) POL2, (G) POL3, and (H) RAD21. (PDF) [file pcbi.1006686.s003.pdf]

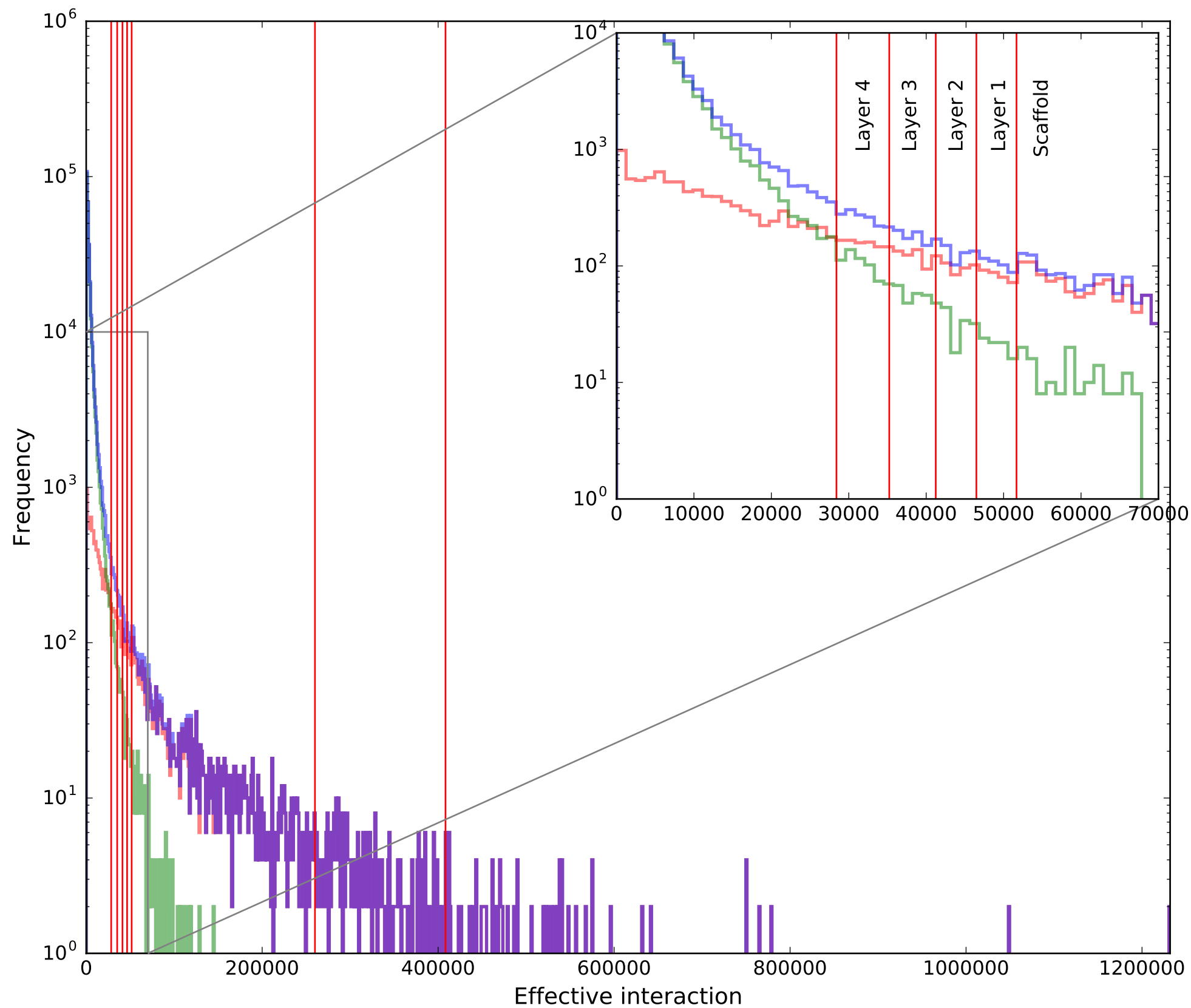

Supplement: S4 Fig — Vertical red lines show the respective cutoff values for classifying interaction strengths into the scaffold layer, and Layers 1 through 4. Scaffold-layer interactions are the strongest 2000 interactions, or the top 1.35% of all interactions. Layer 1 interactions comprise the top 1.35% to 1.5% of all interactions, compared with the scaffold layer. Layer 2 interactions represent the top 1.5% to 1.7% of interactions. Layer 3 interactions represent the top 1.7% to 2.0% of interactions. (PDF) [file pcbi.1006686.s004.pdf]

**A**

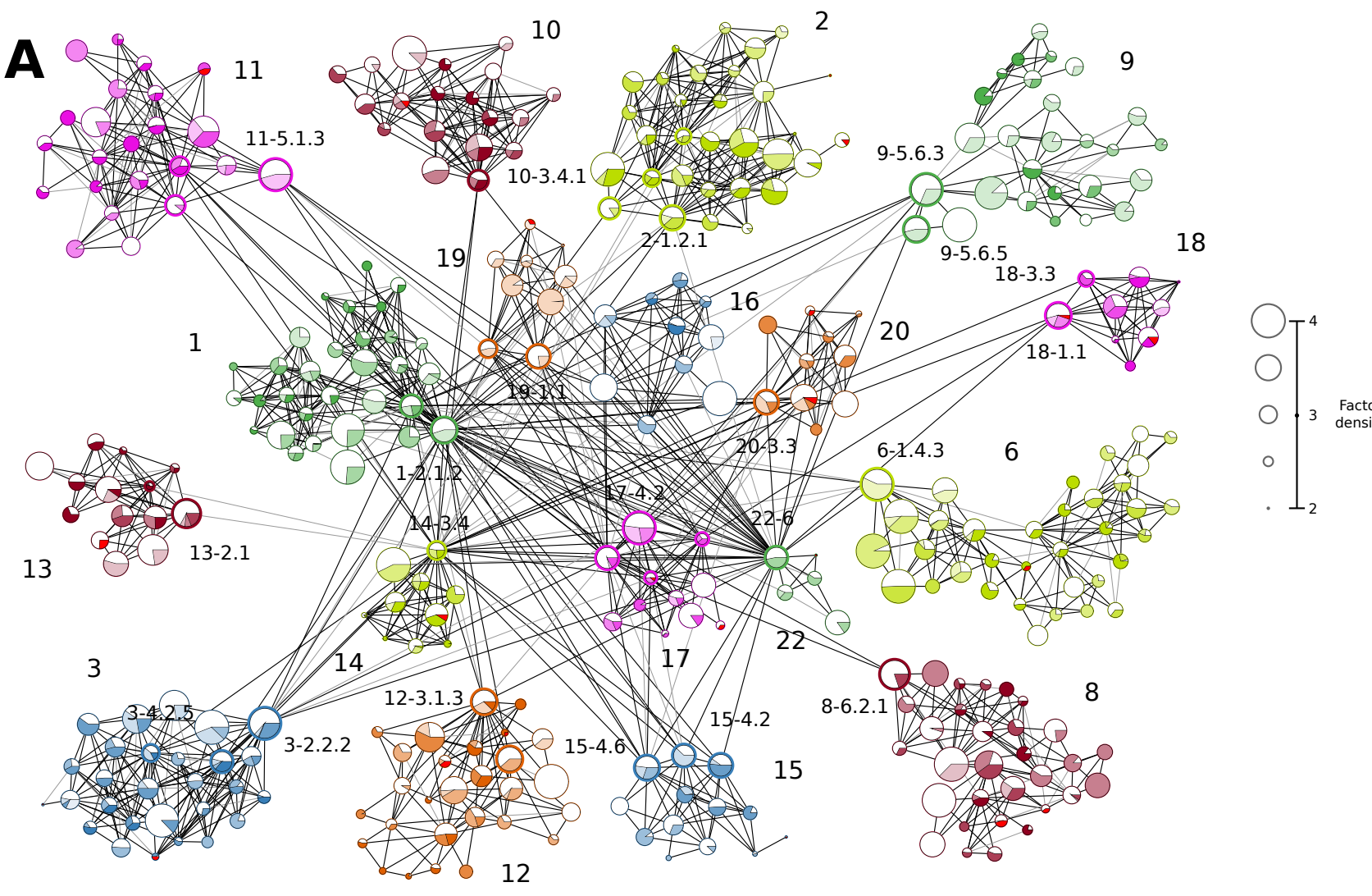

**B**

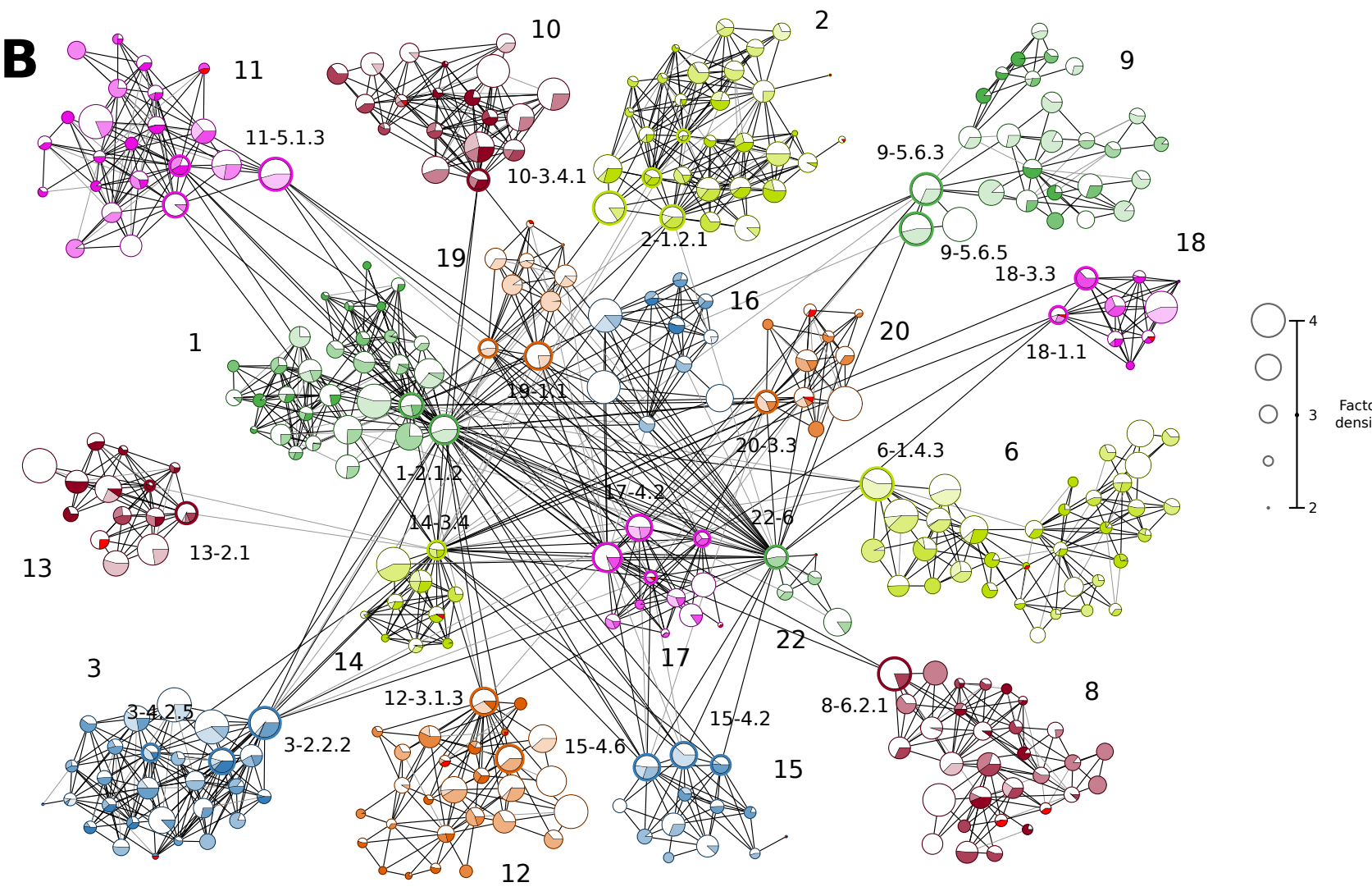

Supplement: S5 Fig — (A) H3K9ac, (B) DNase-Seq. (PDF) [file pcbi.1006686.s005.pdf]

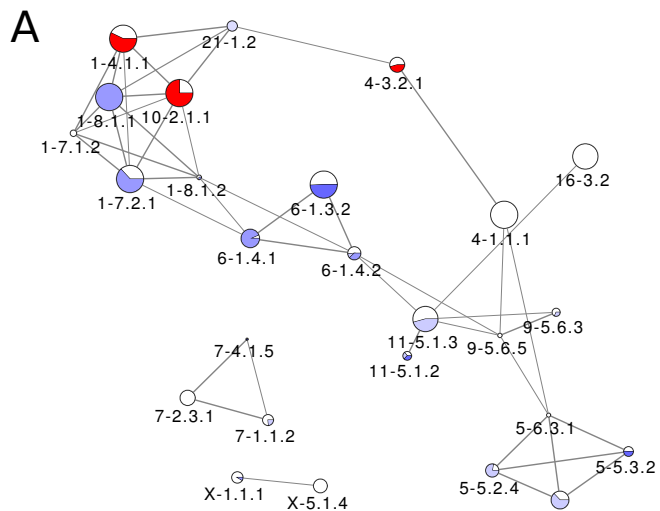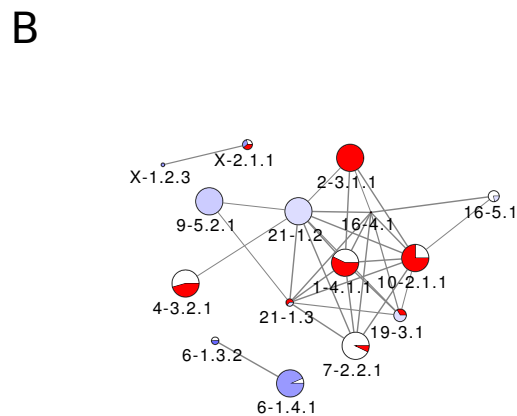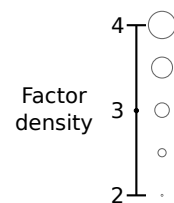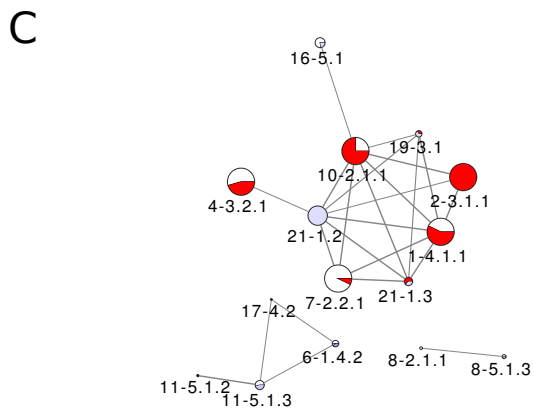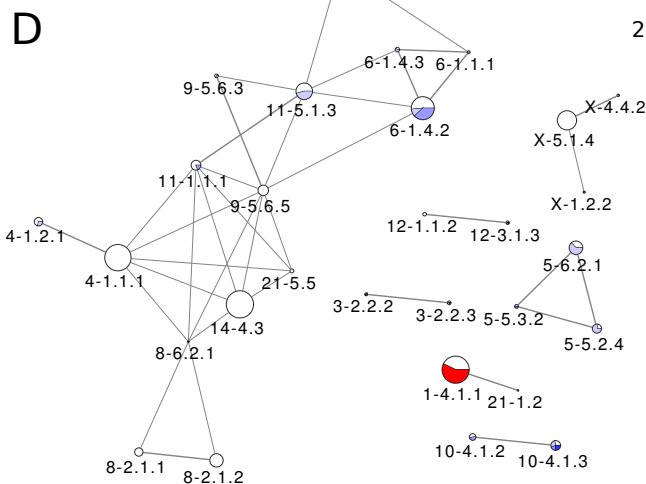

Supplement: S6 Fig — (A) POL2, (B) POL3, (C) RAD21, and (D) CTCF. (PDF) [file pcbi.1006686.s006.pdf]

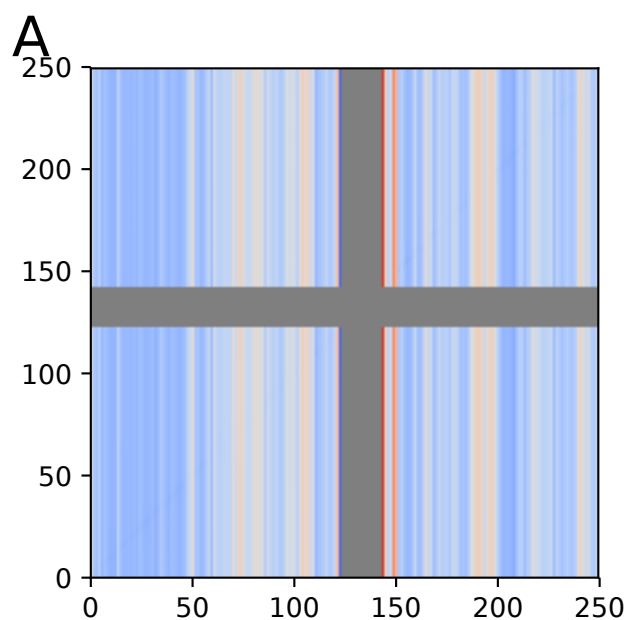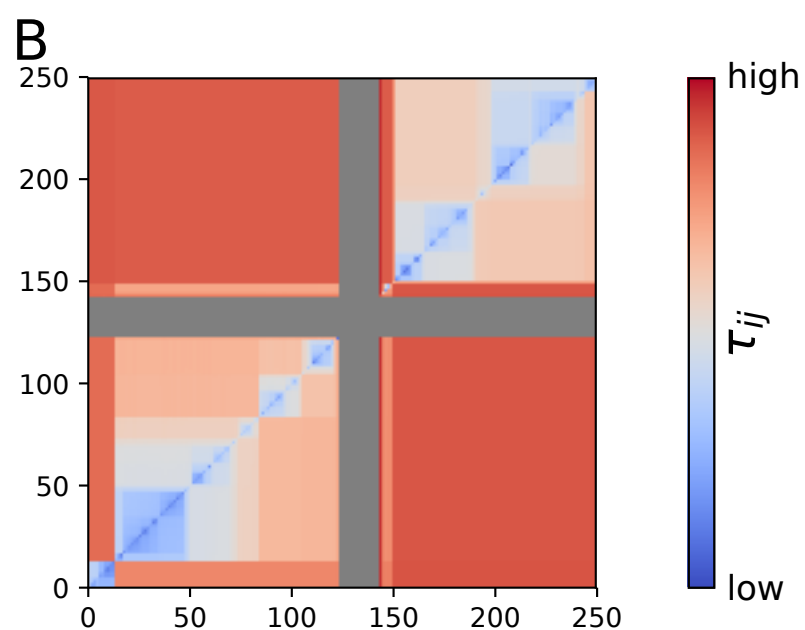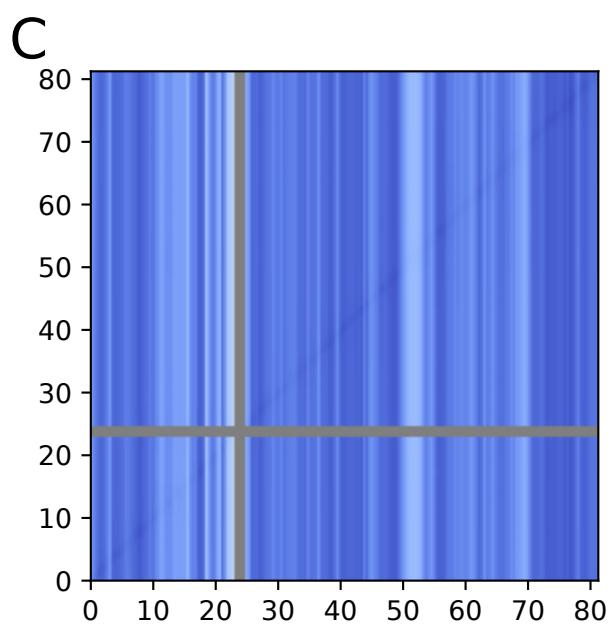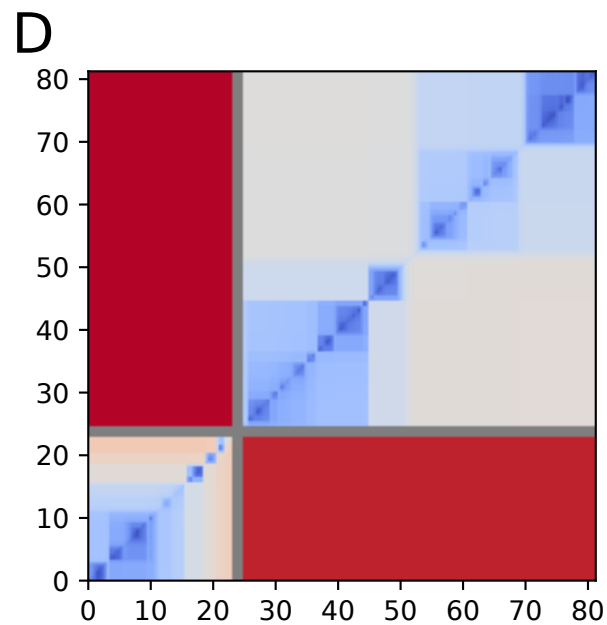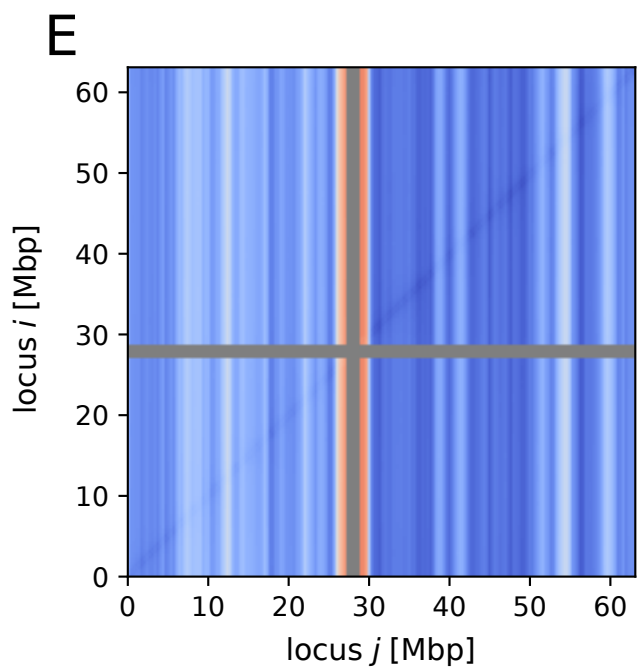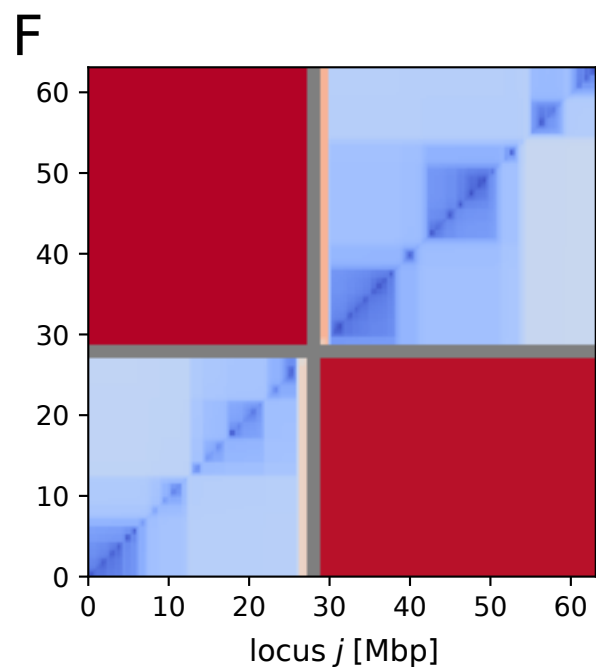

Supplement: S7 Fig — (A) Chromosome 1, β = 1, (B) Chromosome 1, β = 9, (C) Chromosome 17, β = 1, (D) Chromosome 17, β = 9, (E) Chromosome 20, β = 1, (F) Chromosome 20, β = 9. (PDF) [file pcbi.1006686.s007.pdf]

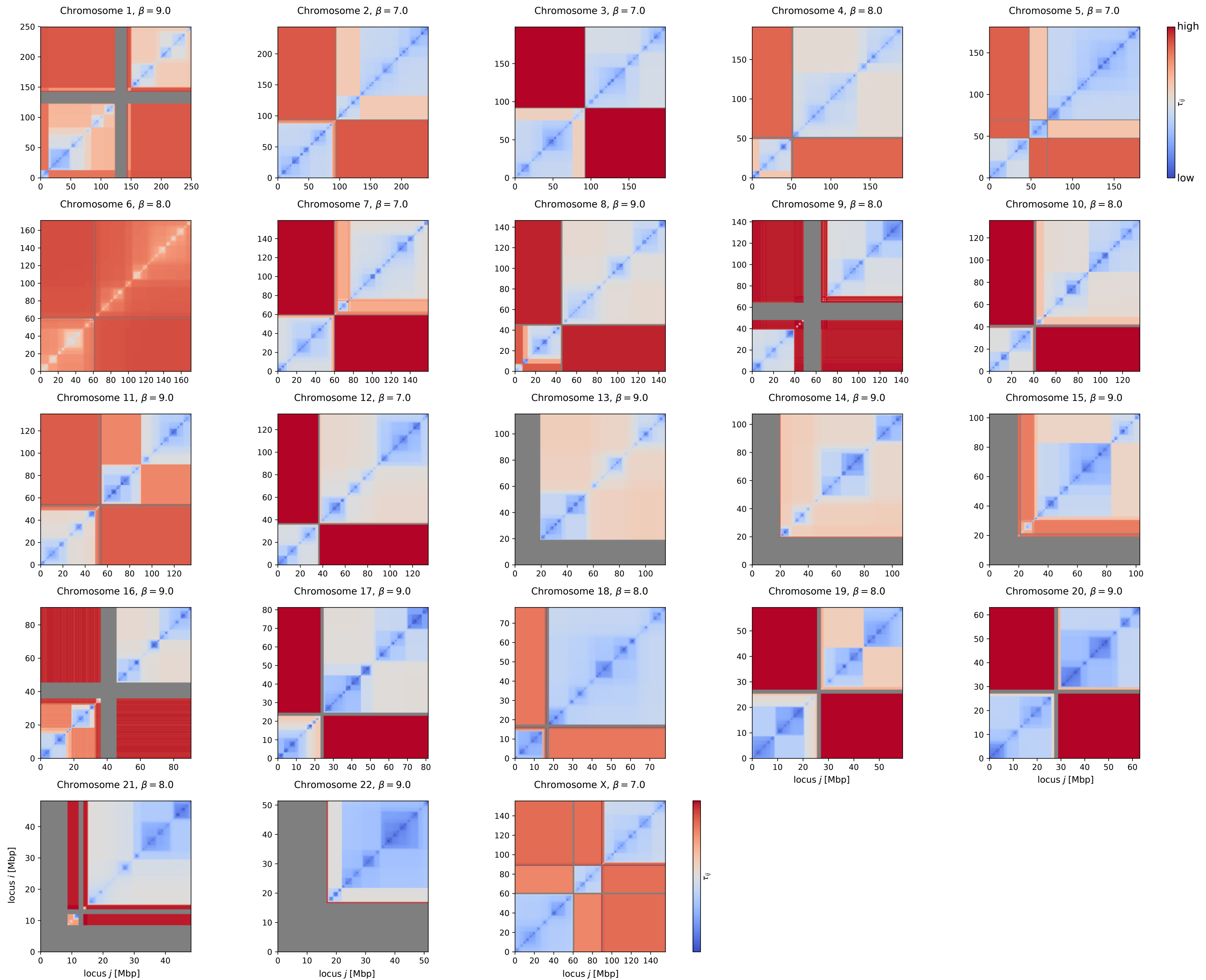

Supplement: S8 Fig — (PDF) [file pcbi.1006686.s008.pdf]

A

## Soft-partitioning

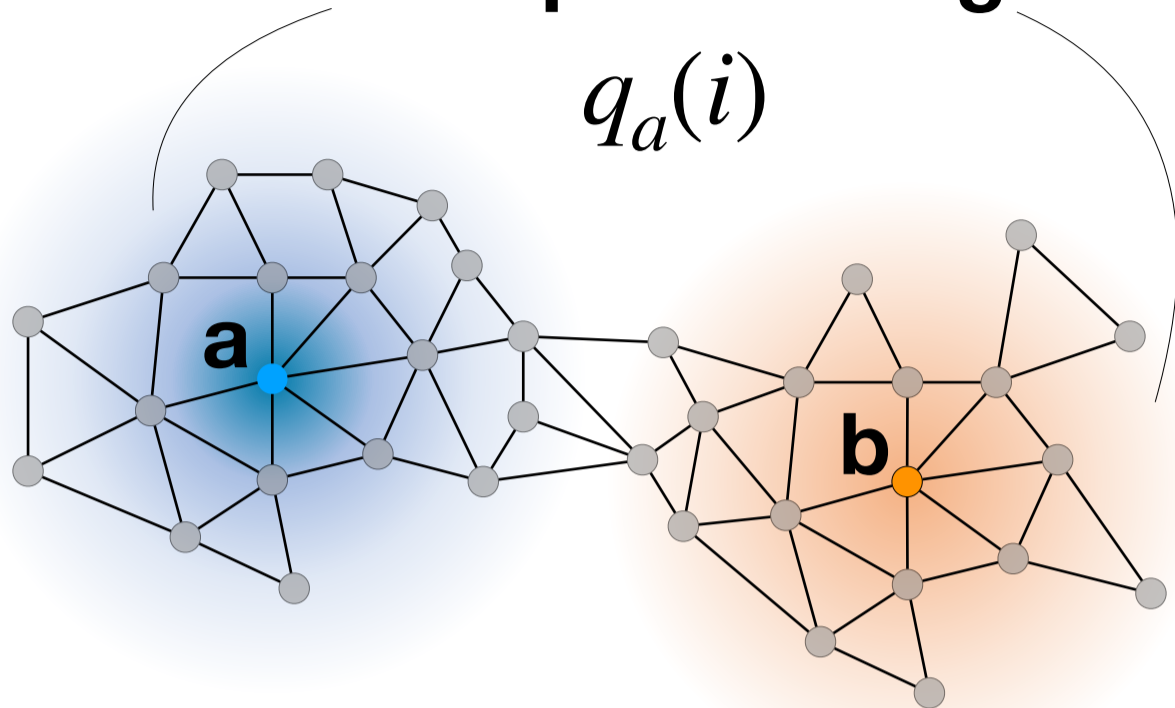

B

## Hard-partitioning

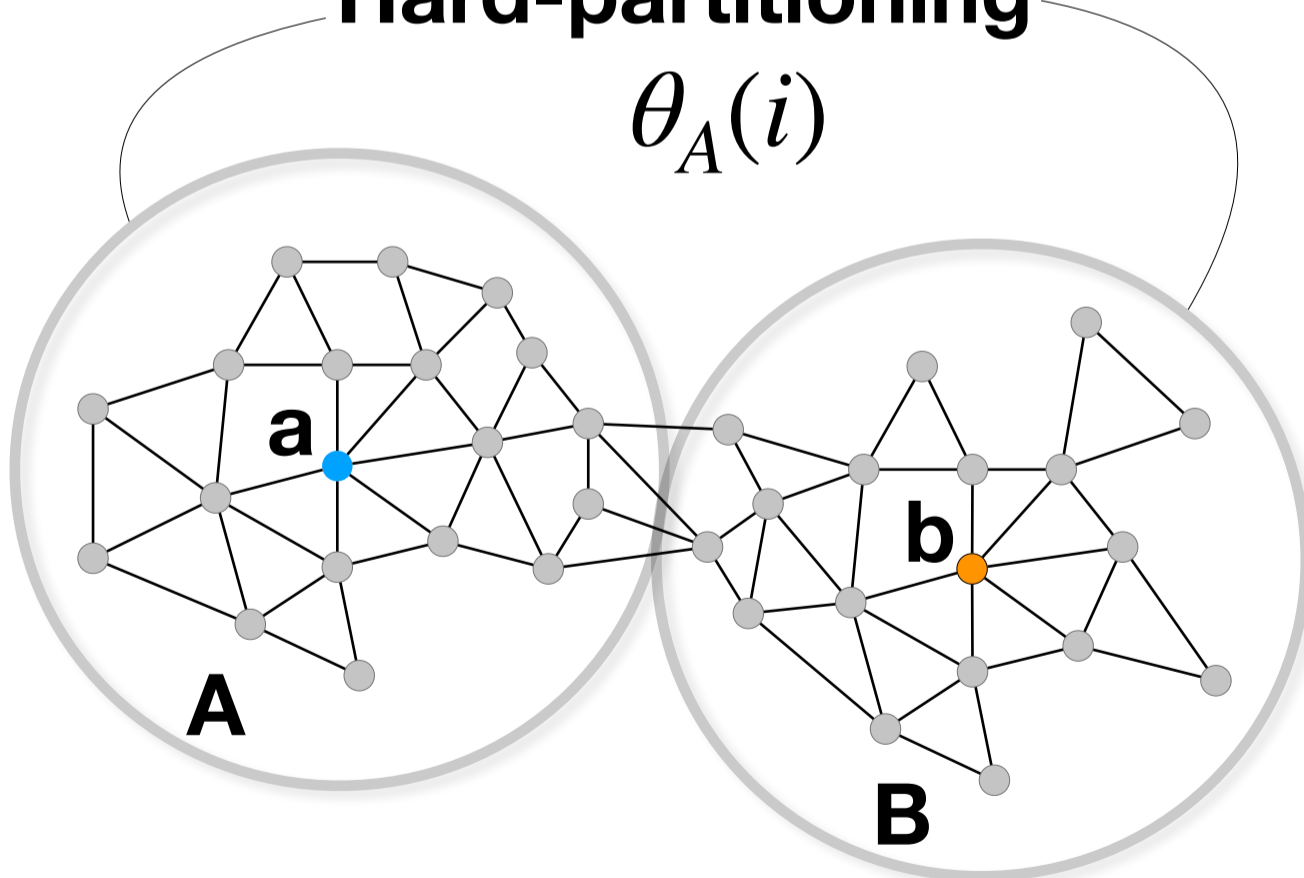

Supplement: S9 Fig — Illustration of the difference between the concepts of (A) soft partitioning and (B) hard partitioning. (PDF) [file pcbi.1006686.s009.pdf]

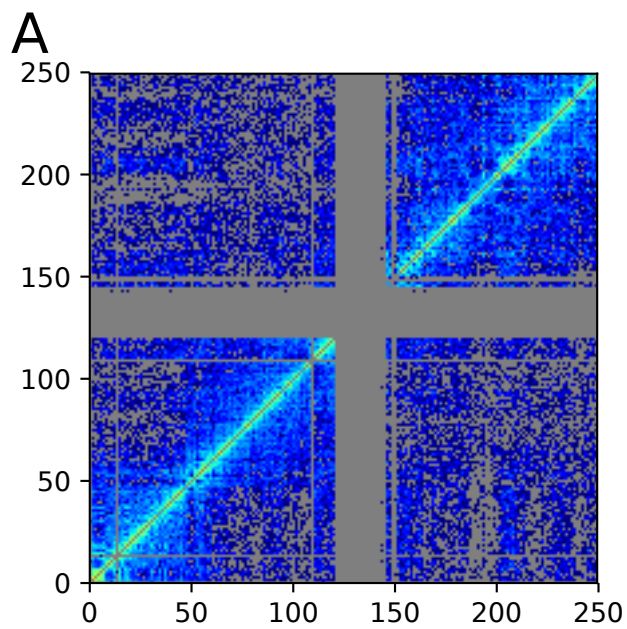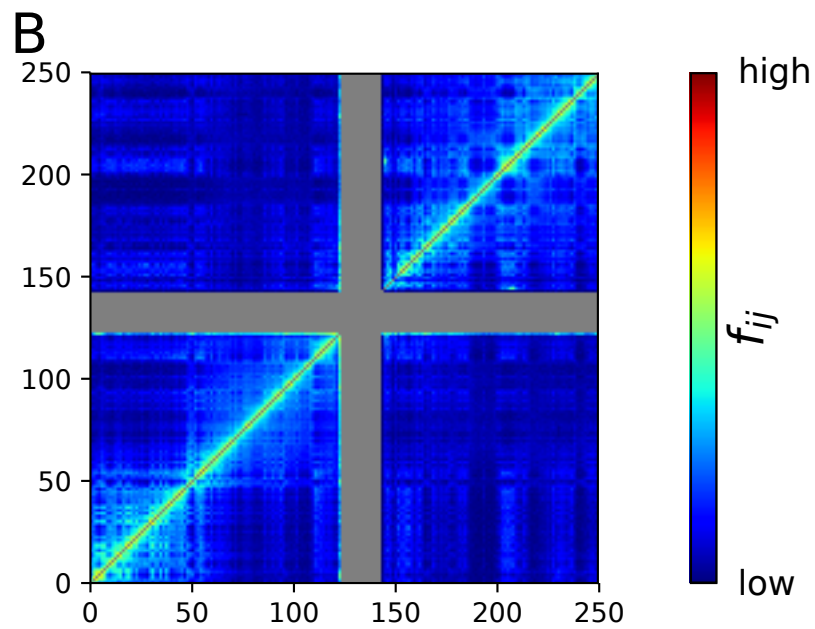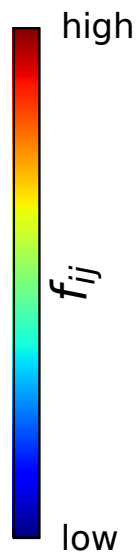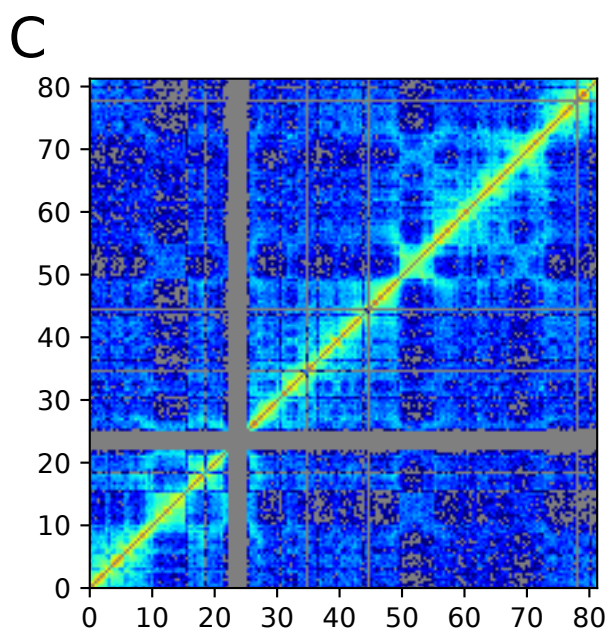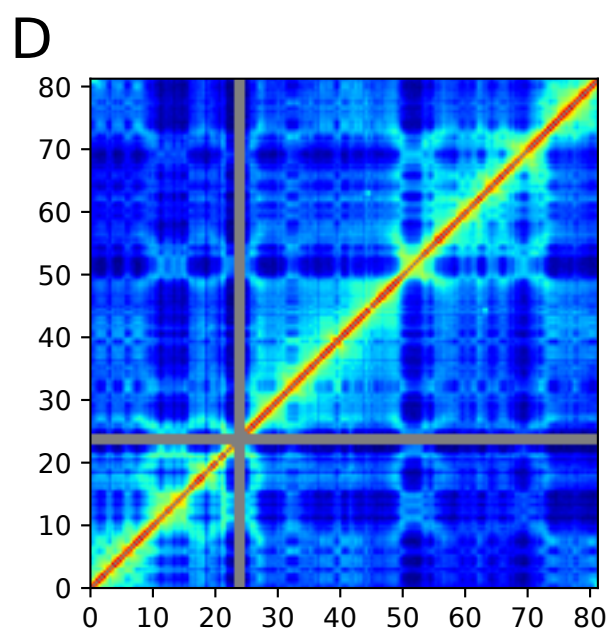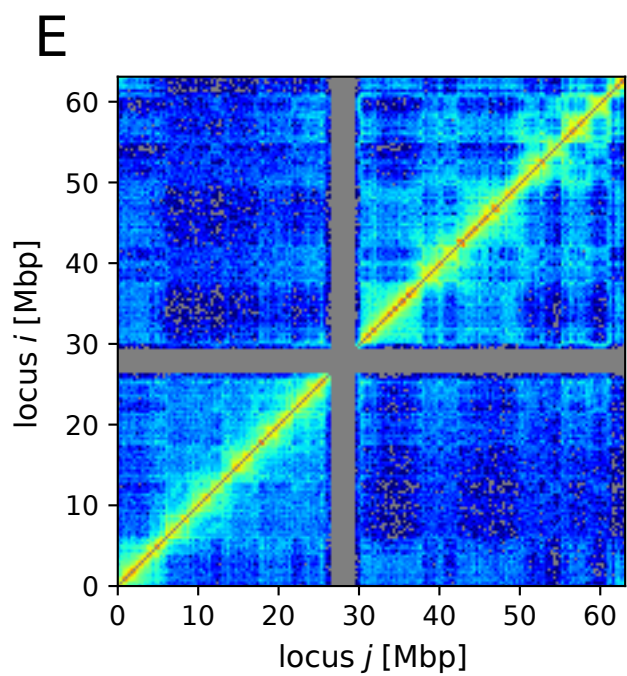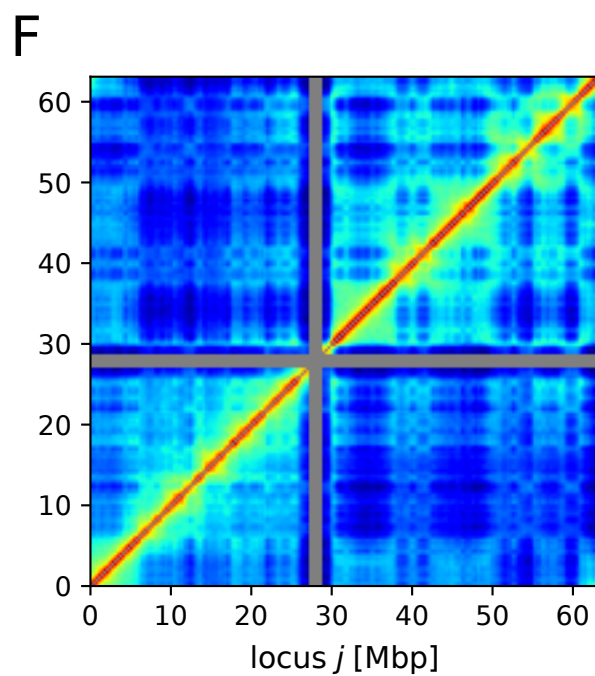

Supplement: S11 Fig — Comparison of the raw Hi-C data matrix with the result of Gaussian Filter (GF) preprocessing on (A-B) chromosome 1, (C-D) chromosome 17, and (E-F) chromosome 20. The width parameter σ = 200kbp, truncated at 4σ, was used for the Gaussian kernel. The original resolution of Hi-C data was 50kbp resolution. The two columns represent (A, C, E) raw matrices and (B, D, F) GF-preprocessed matrices. (PDF) [file pcbi.1006686.s011.pdf]

A

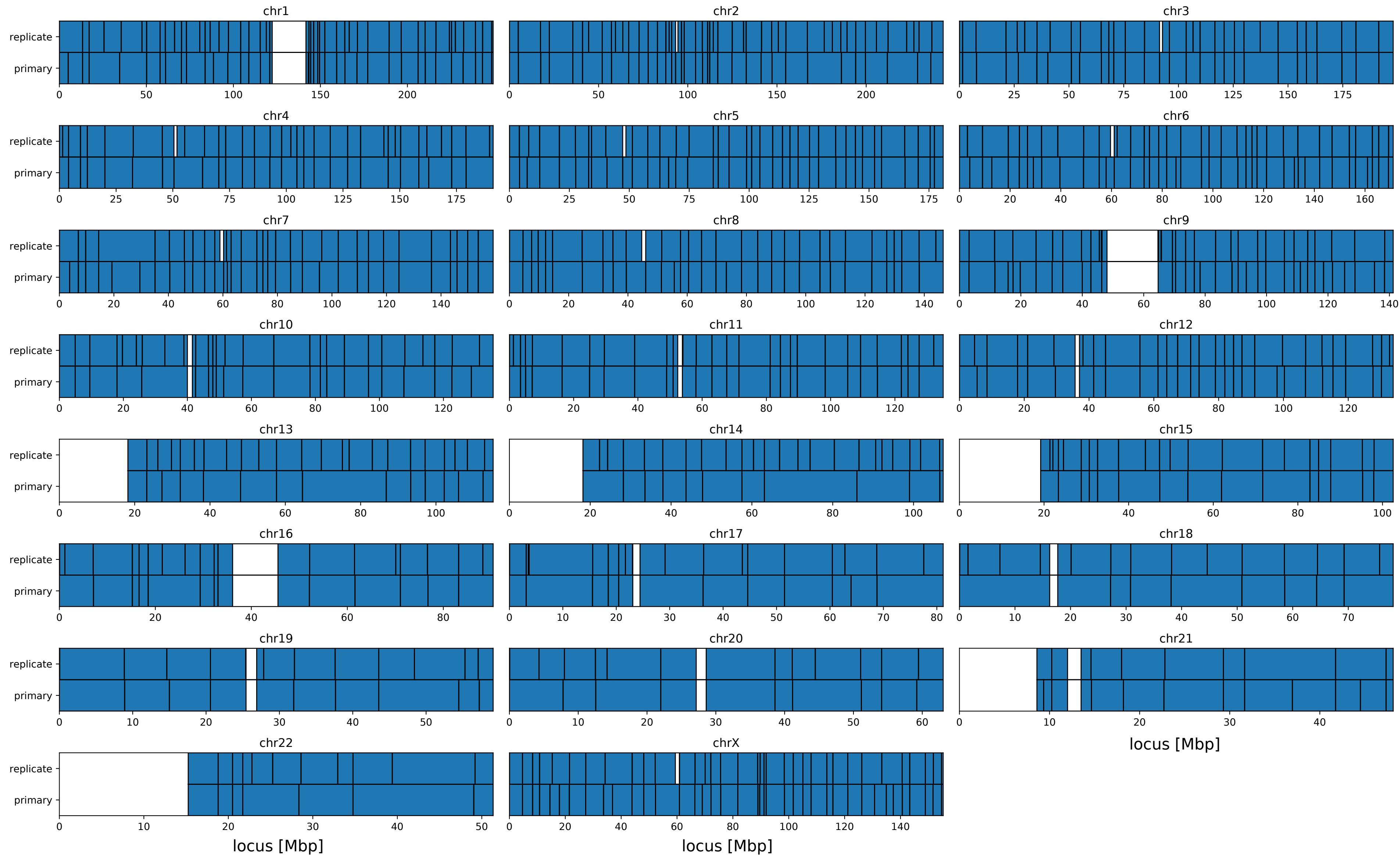

B

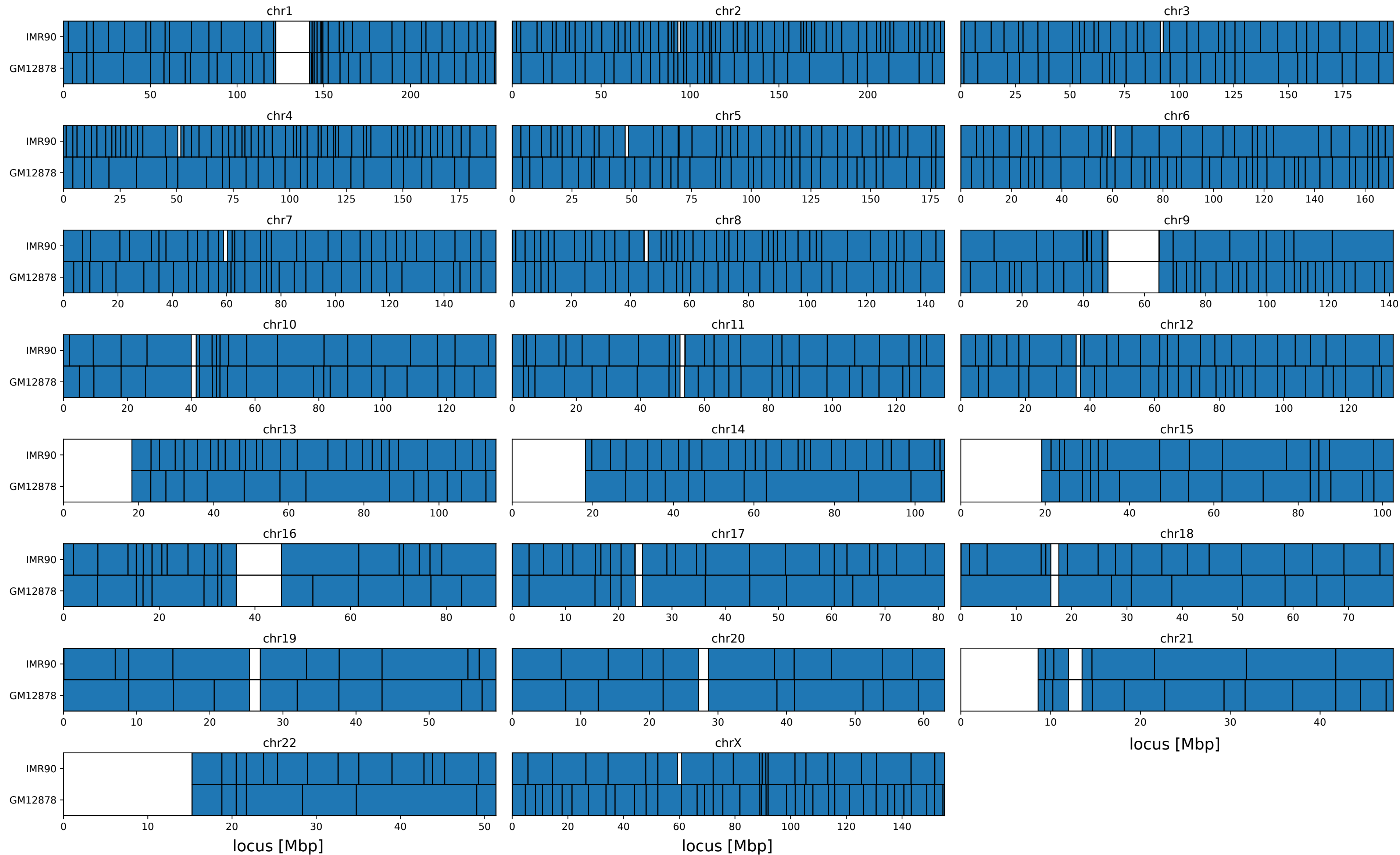

C

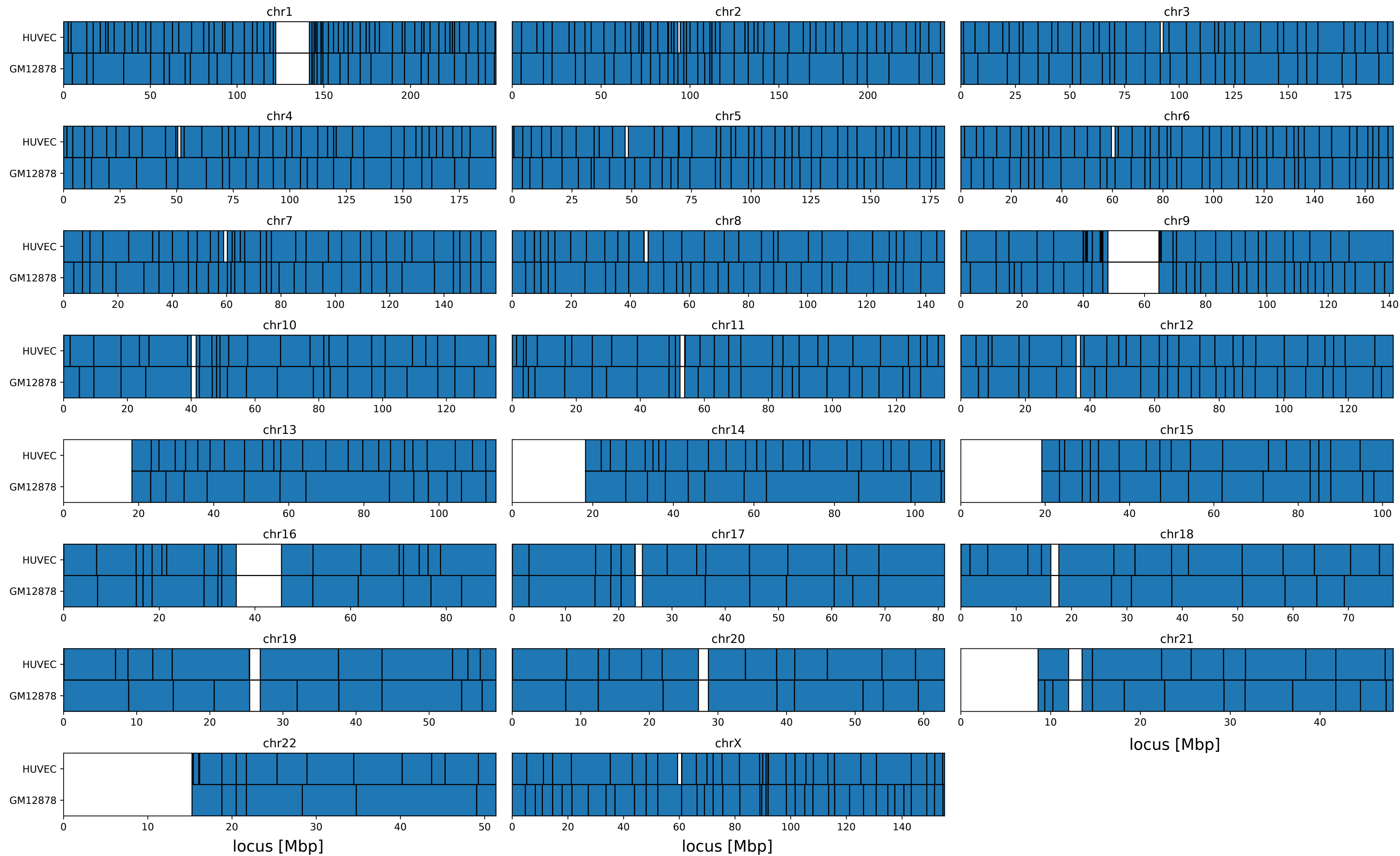

D

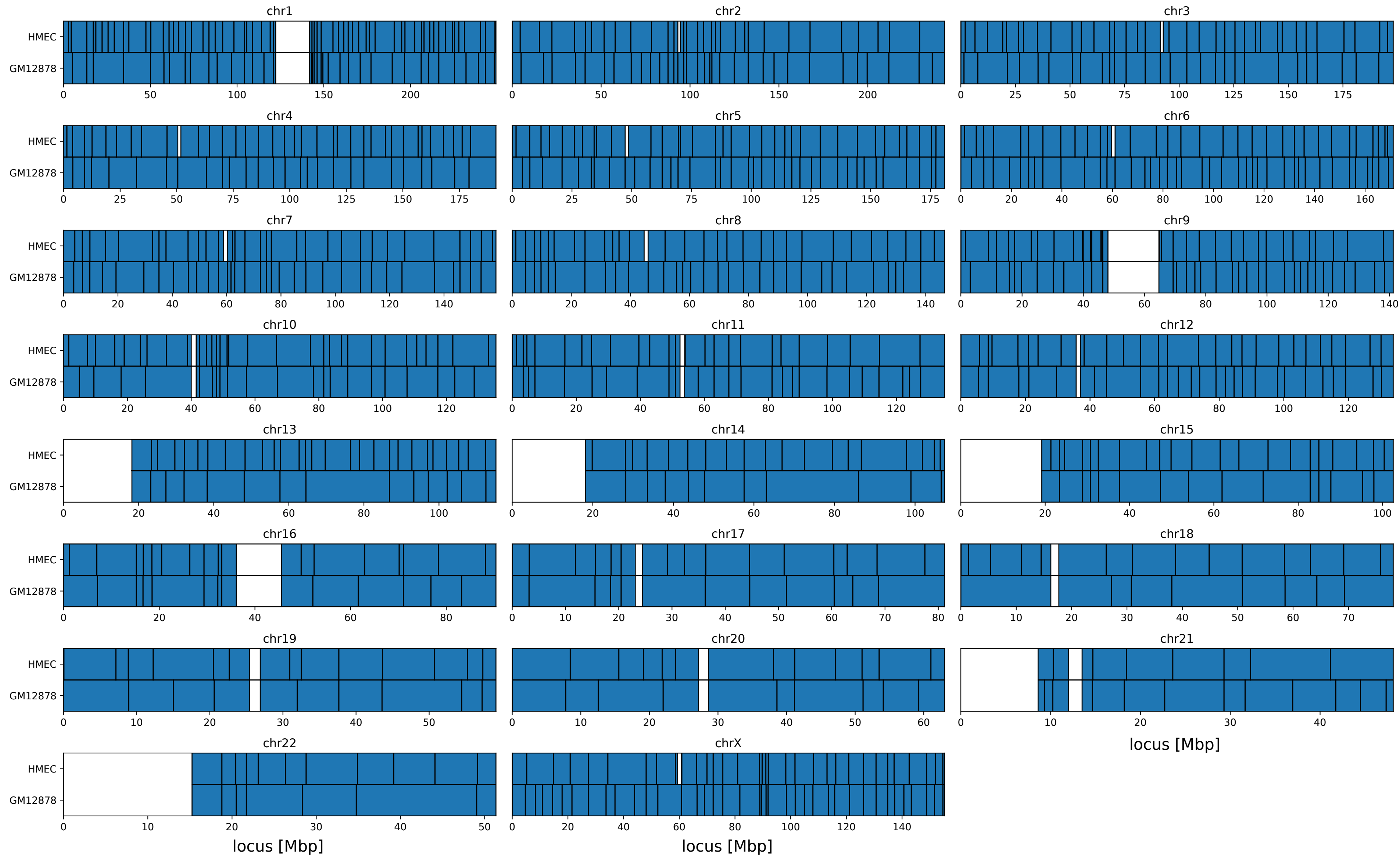

Supplement: S13 Fig — (A) GM12878_replicate, (B) IMR90, (C) HUVEC, (D) HMEC. (PDF) [file pcbi.1006686.s013.pdf]

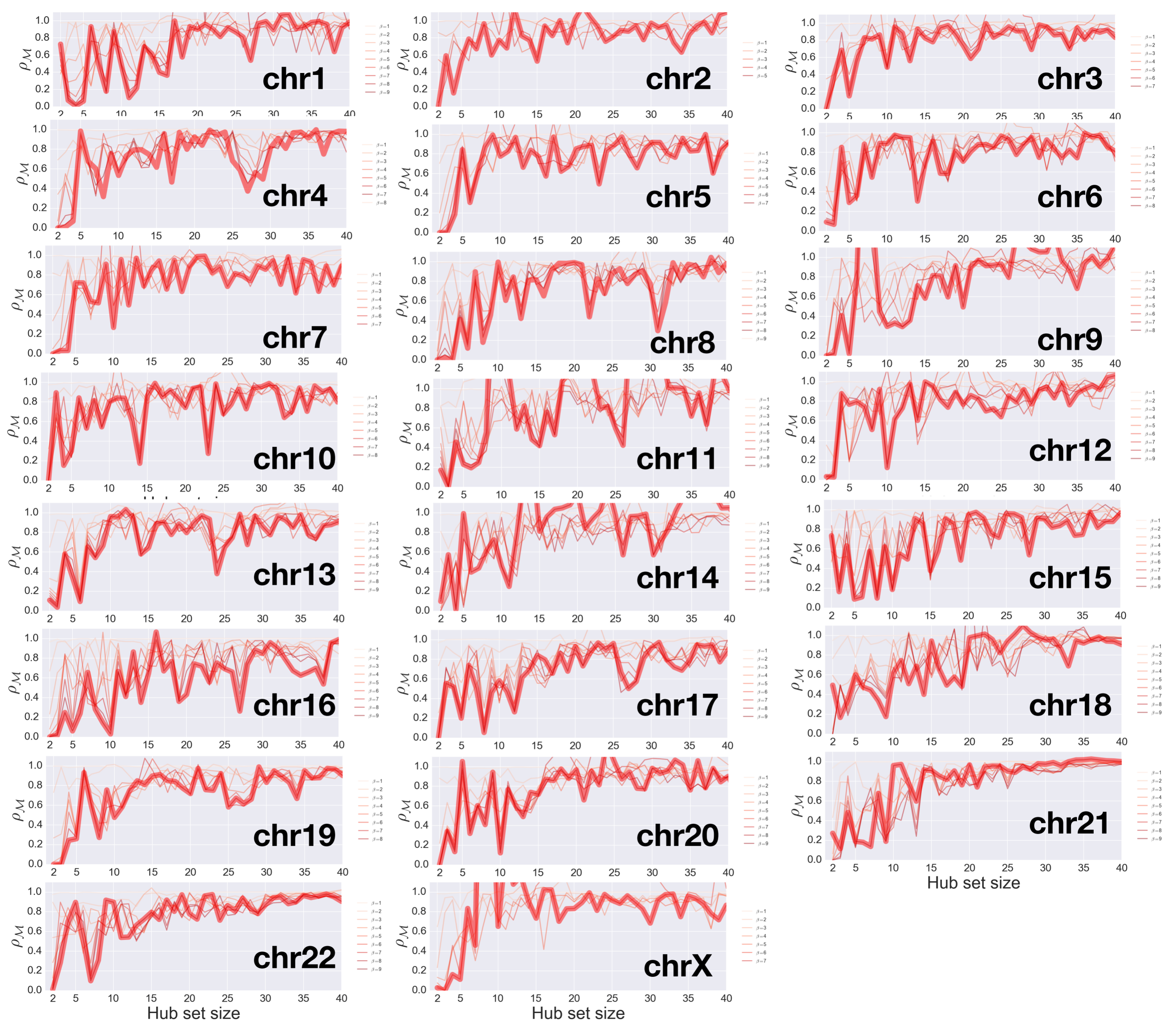

Supplement: S14 Fig — (PDF) [file pcbi.1006686.s014.pdf]

[illegible]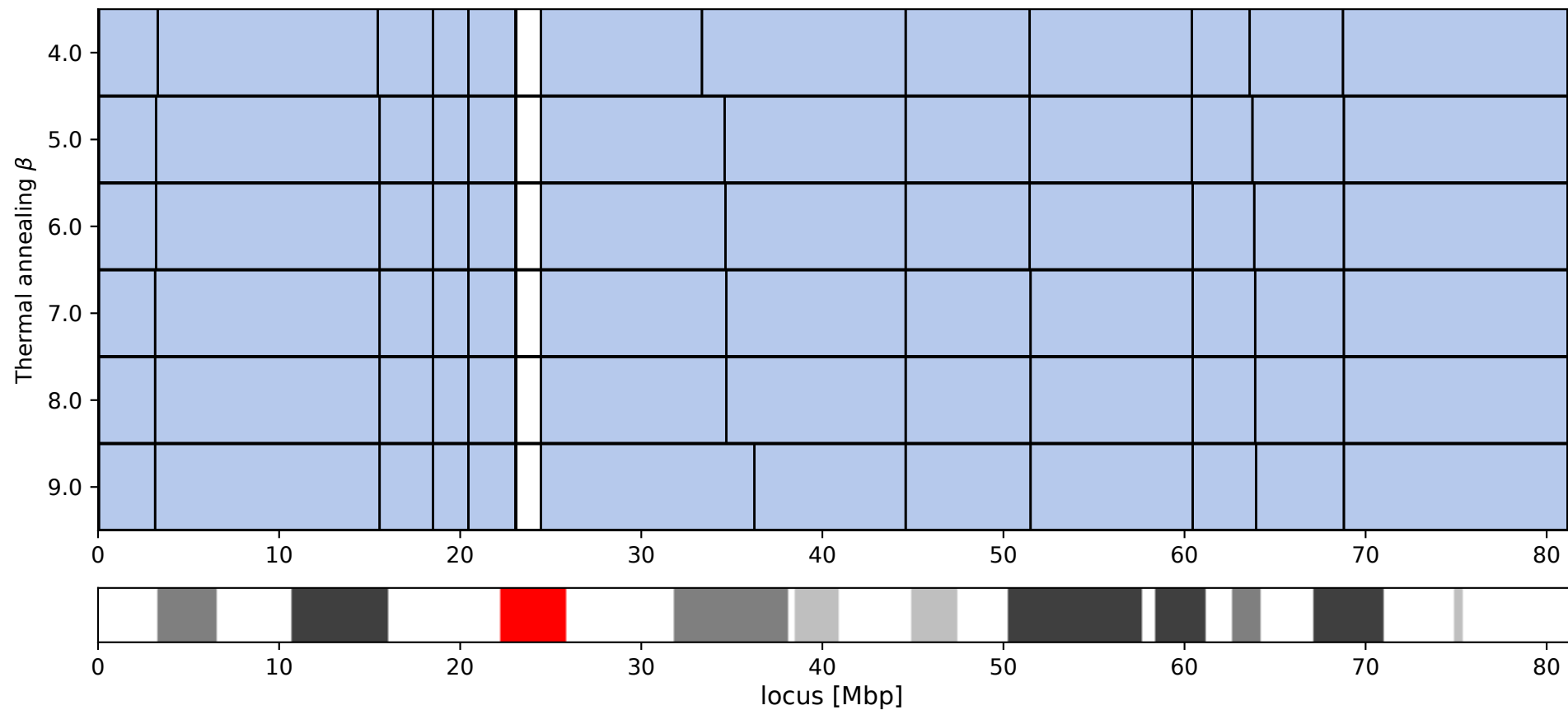

Supplement: S15 Fig — In the regime of high β, partitioning boundaries tend to be stable with respect to changes in the value of β. (PDF) [file pcbi.1006686.s015.pdf]

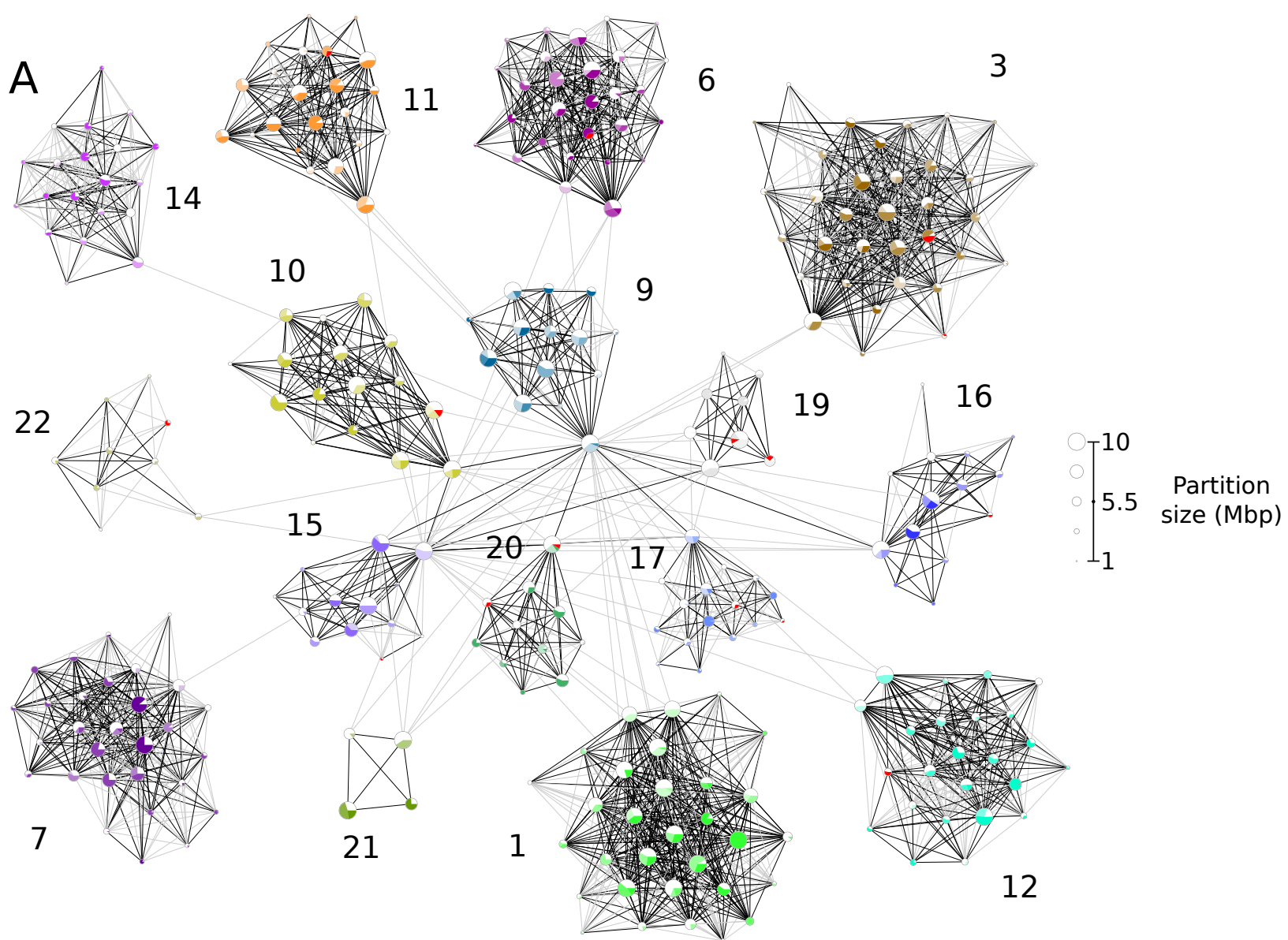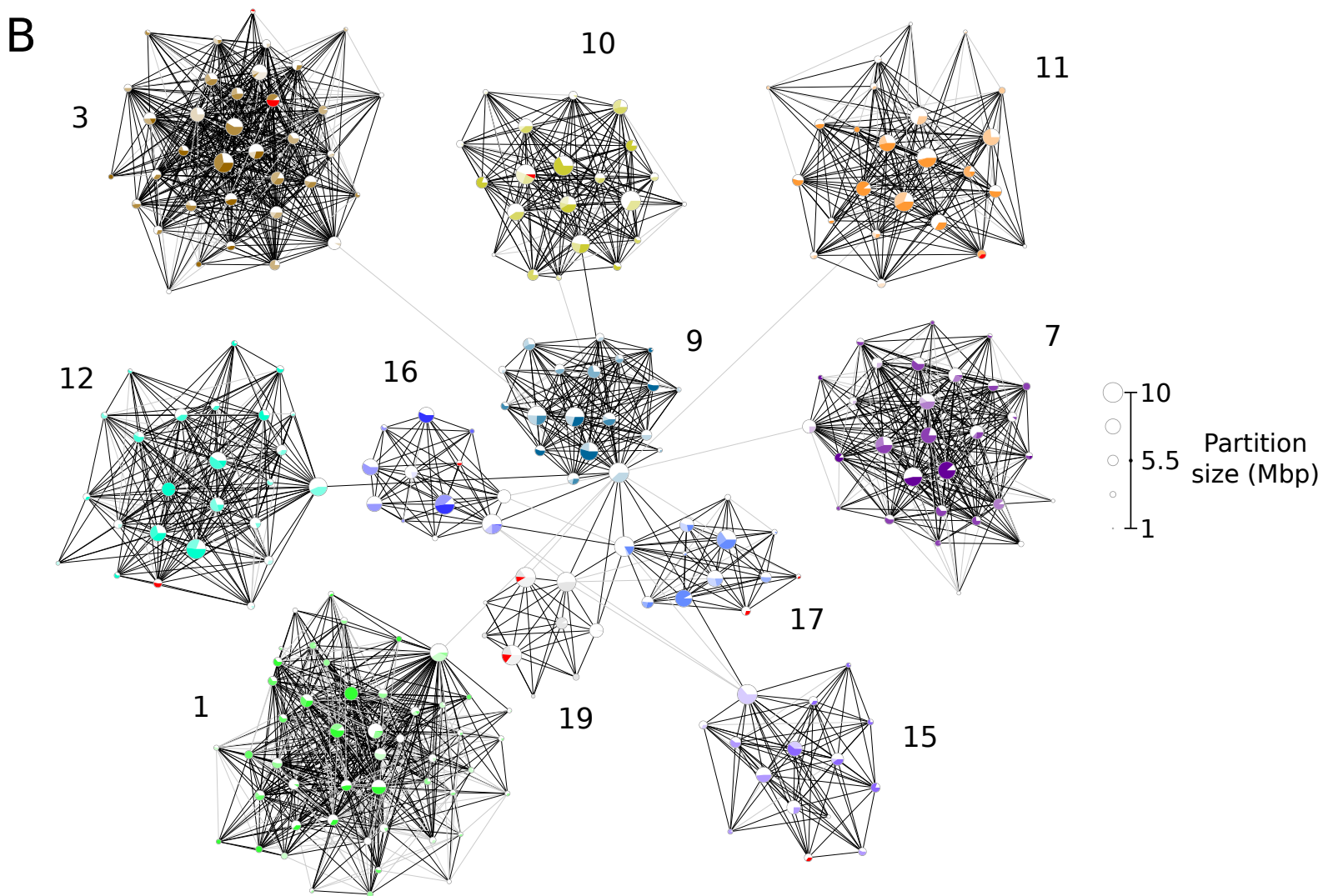

Supplement: S16 Fig — (A) IMR90, (B) HUVEC. (PDF) [file pcbi.1006686.s016.pdf]
