# Supplementary material for: Exploring chromatin hierarchical organization via Markov State Modelling
Source: PLoS Comput Biol. 2018 Dec 31;14(12):e1006686. doi: 10.1371/journal.pcbi.1006686 (PMC6355033; doi:10.1371/journal.pcbi.1006686)
Supplement: S10 Fig — In each of the following cases, we plot the partitioning diagrams for a chromosome at all levels that have metastability index ρM below a threshold value ρc: (A) Chromosome 1, ρc = 0.5, (B) Chromosome 17, ρc = 0.5, (C) Chromosome 20, ρc = 0.5, (D) Chromosome 1, ρc = 0.8, (E) Chromosome 17, ρc = 0.8, (F) Chromosome 20, ρc = 0.8. (PDF) [file pcbi.1006686.s010.pdf]

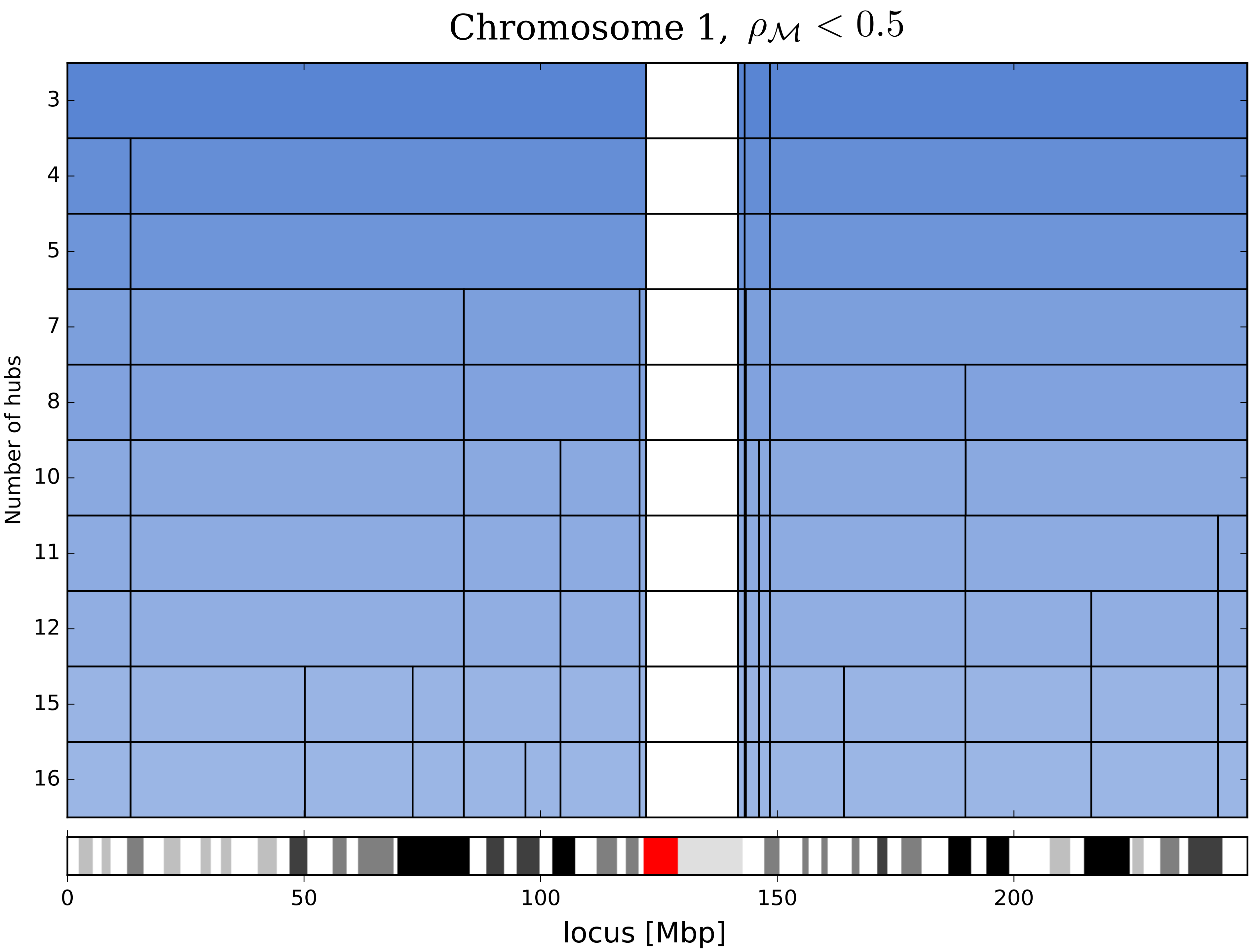

Chromosome 1, $\rho_{\mathcal{M}} < 0.5$
Number of hubs
3
4
5
7
8
10
11
12
15
16
0
50
100
150
200
locus [Mbp]

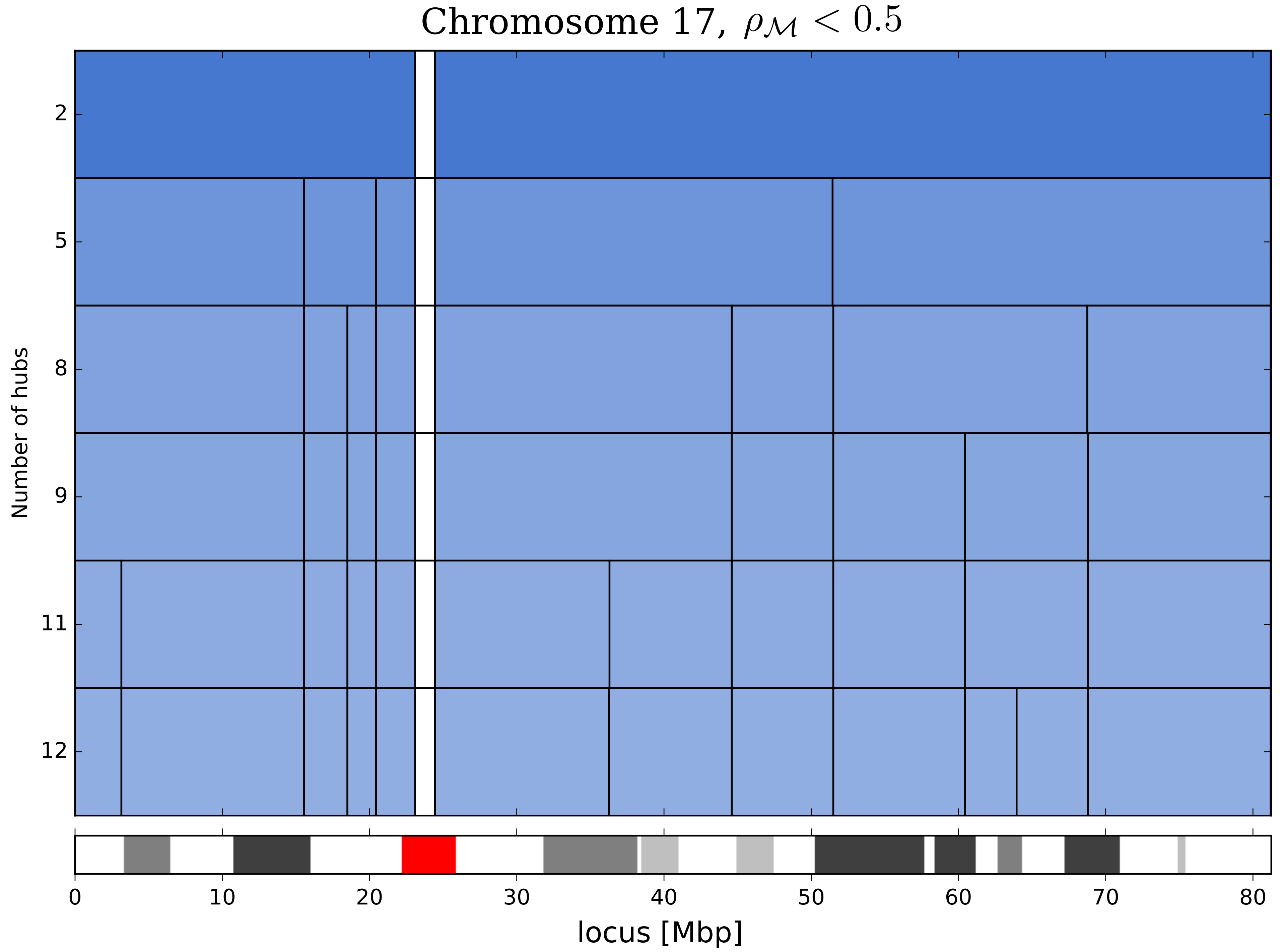

Chromosome 17, $\rho_{\mathcal{M}} < 0.5$
Number of hubs
2
5
8
9
11
12
0
10
20
30
40
50
60
70
80
locus [Mbp]

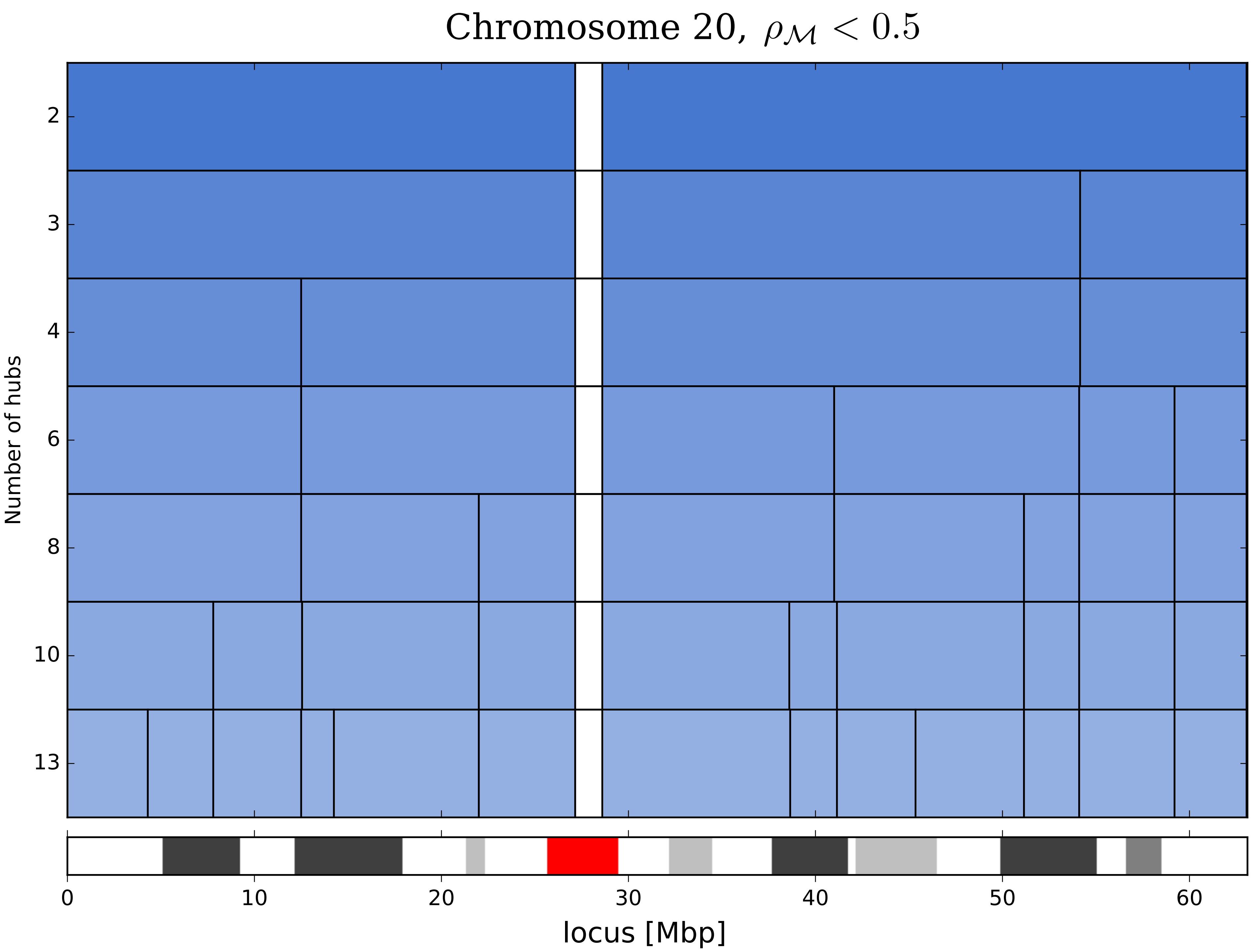
Chromosome 20, $\rho_{\mathcal{M}} < 0.5$
Number of hubs
2
3
4
6
8
10
13
0
10
20
30
40
50
60
locus [Mbp]

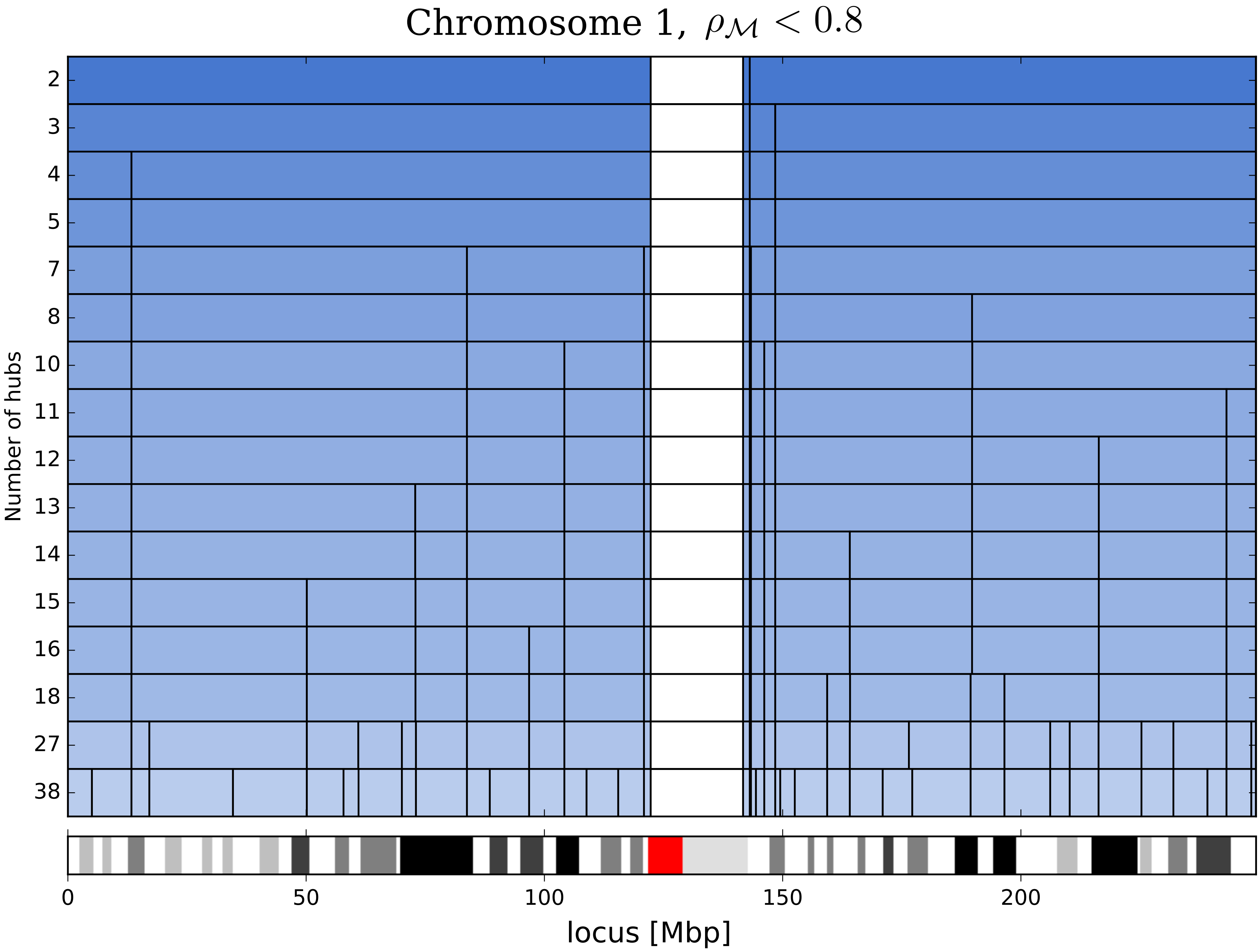
Chromosome 1, $\rho_{\mathcal{M}} < 0.8$
Number of hubs
2
3
4
5
7
8
10
11
12
13
14
15
16
18
27
38
0
50
100
150
200
locus [Mbp]

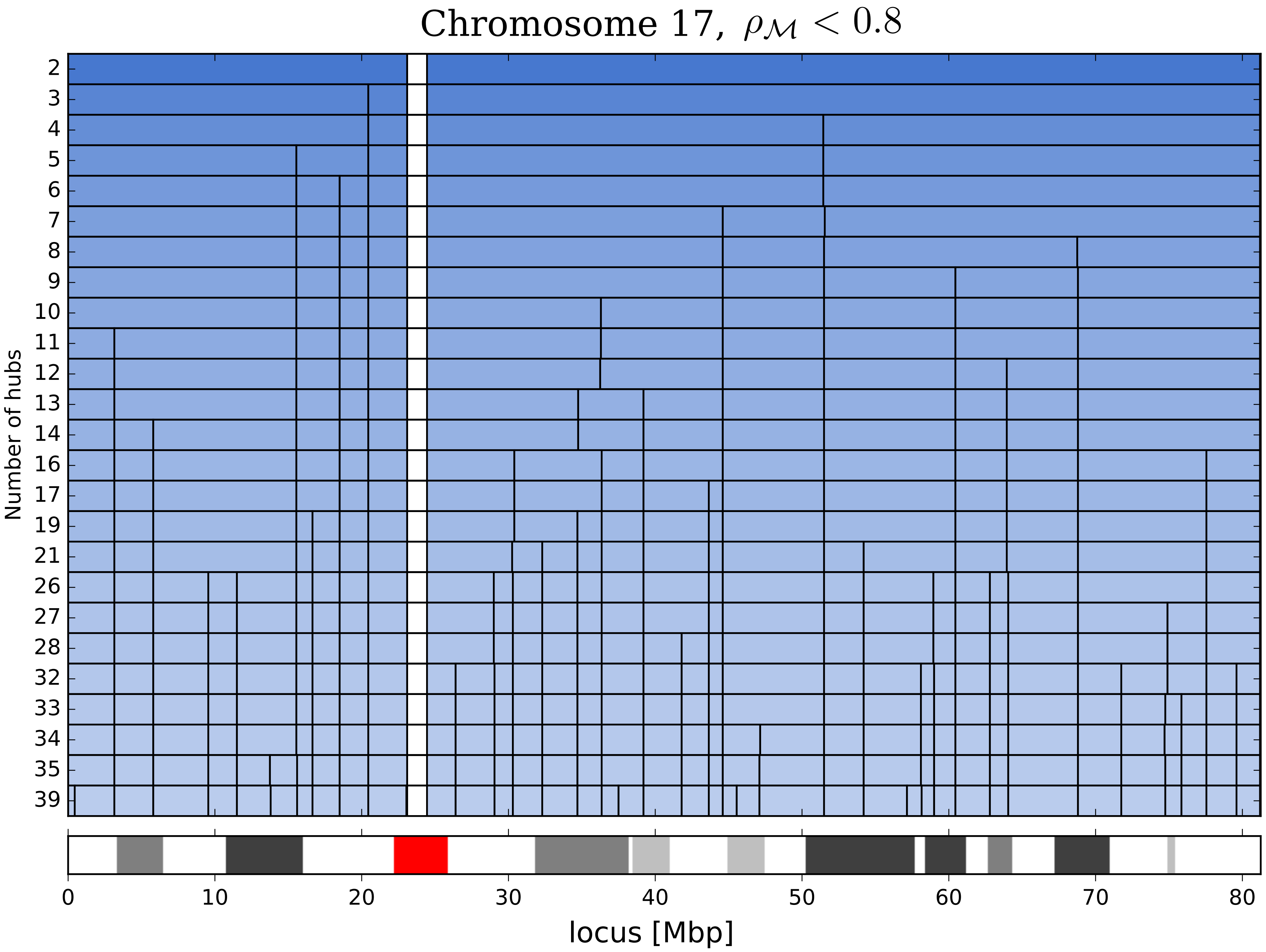

Chromosome 17, $\rho_{\mathcal{M}} < 0.8$
Number of hubs
2
3
4
5
6
7
8
9
10
11
12
13
14
16
17
19
21
26
27
28
32
33
34
35
39
0
10
20
30
40
50
60
70
80
locus [Mbp]

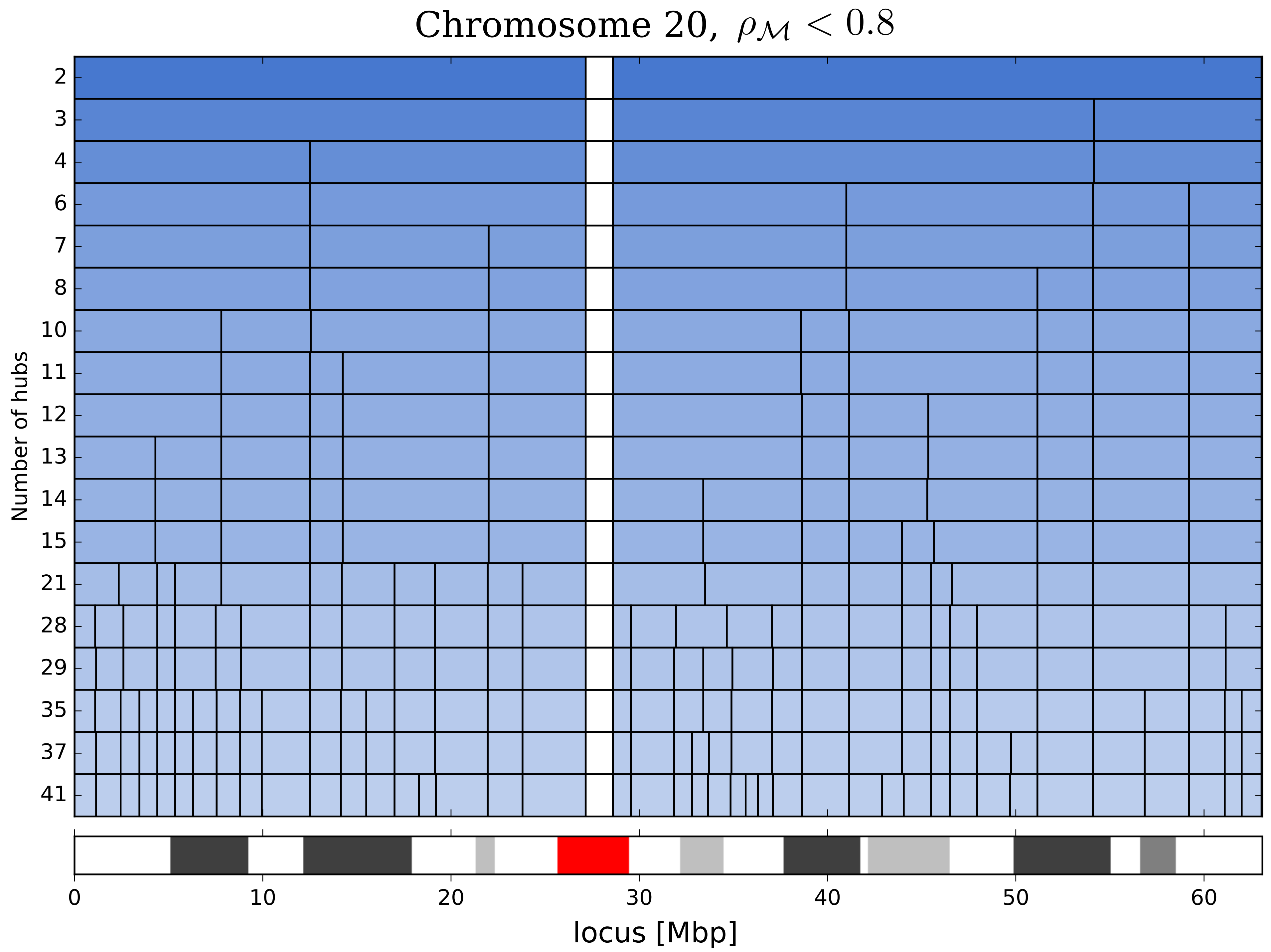
Chromosome 20, $\rho_{\mathcal{M}} < 0.8$
Number of hubs
2
3
4
6
7
8
10
11
12
13
14
15
21
28
29
35
37
41
0
10
20
30
40
50
60
locus [Mbp]
