# Supplementary material for: Exploring chromatin hierarchical organization via Markov State Modelling
Source: PLoS Comput Biol. 2018 Dec 31;14(12):e1006686. doi: 10.1371/journal.pcbi.1006686 (PMC6355033; doi:10.1371/journal.pcbi.1006686)
Supplement: S12 Fig — Each panel shows the factor enrichment levels for (A) euchromatin-euchromatin partition pairs, (B) heterochromatin-heterochromatin pairs, and (C) euchromatin-heterochromatin pairs. We defined euchromatic partitions (EC) as those with more than 80% of the partition having stain levels [61] G-negative or G-positive25, and heterochromatic partitions (HC) as those with more than 80% of the partition having stain levels G-positive50, G-positive75, or G-positive100. For each interaction pair, we computed the product of ChIP-Seq signal values for each of the transcription factors and histone modifications studied in this work, and plotted it against the effective interaction strength as a density plot. We show a log-log regression line for each case, stating the r-value and p-value in each case. (PDF) [file pcbi.1006686.s012.pdf]

A

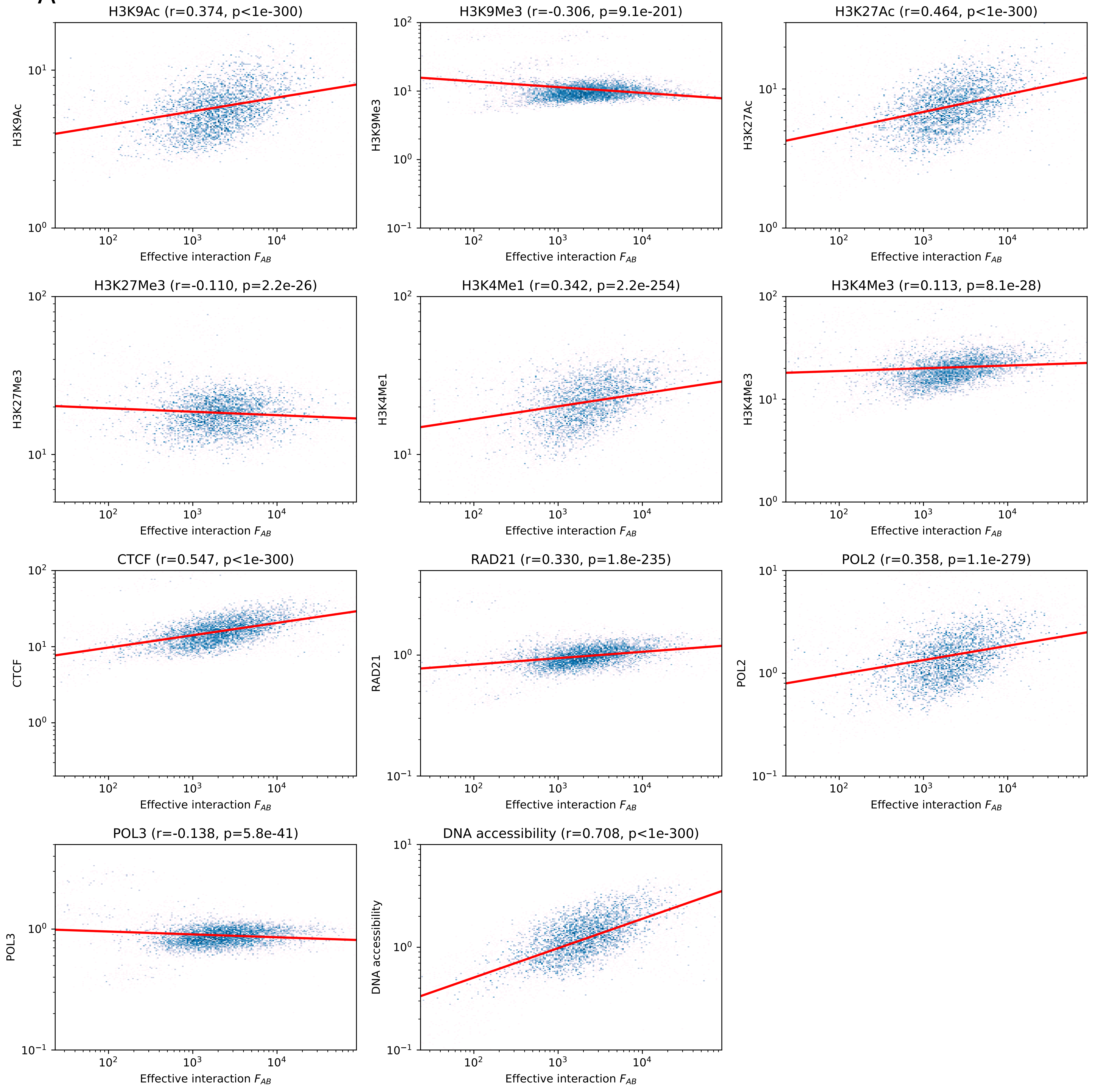
H3K9Ac (r=0.374, p<1e-300)
H3K9Ac
Effective interaction F_{AB}
H3K9Me3 (r=-0.306, p=9.1e-201)
H3K9Me3
Effective interaction F_{AB}
H3K27Ac (r=0.464, p<1e-300)
H3K27Ac
Effective interaction F_{AB}
H3K27Me3 (r=-0.110, p=2.2e-26)
H3K27Me3
Effective interaction F_{AB}
H3K4Me1 (r=0.342, p=2.2e-254)
H3K4Me1
Effective interaction F_{AB}
H3K4Me3 (r=0.113, p=8.1e-28)
H3K4Me3
Effective interaction F_{AB}
CTCF (r=0.547, p<1e-300)
CTCF
Effective interaction F_{AB}
RAD21 (r=0.330, p=1.8e-235)
RAD21
Effective interaction F_{AB}
POL2 (r=0.358, p=1.1e-279)
POL2
Effective interaction F_{AB}
POL3 (r=-0.138, p=5.8e-41)
POL3
Effective interaction F_{AB}
DNA accessibility (r=0.708, p<1e-300)
DNA accessibility
Effective interaction F_{AB}

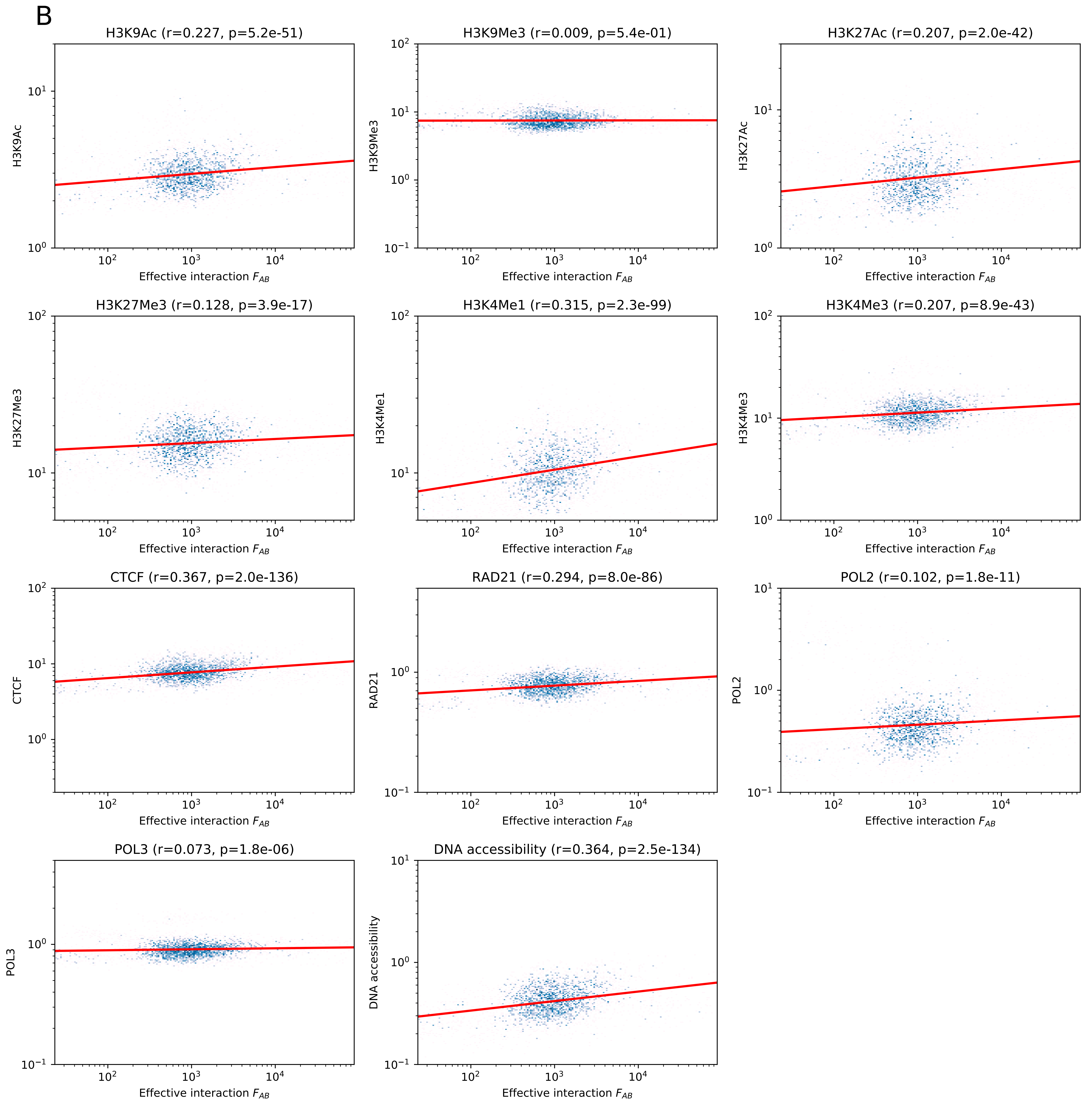

B
H3K9Ac (r=0.227, p=5.2e-51)
H3K9Me3 (r=0.009, p=5.4e-01)
H3K27Ac (r=0.207, p=2.0e-42)
H3K27Me3 (r=0.128, p=3.9e-17)
H3K4Me1 (r=0.315, p=2.3e-99)
H3K4Me3 (r=0.207, p=8.9e-43)
CTCF (r=0.367, p=2.0e-136)
RAD21 (r=0.294, p=8.0e-86)
POL2 (r=0.102, p=1.8e-11)
POL3 (r=0.073, p=1.8e-06)
DNA accessibility (r=0.364, p=2.5e-134)
H3K9Ac
H3K9Me3
H3K27Ac
H3K27Me3
H3K4Me1
H3K4Me3
CTCF
RAD21
POL2
POL3
DNA accessibility
Effective interaction F_{AB}
10^2
10^3
10^4

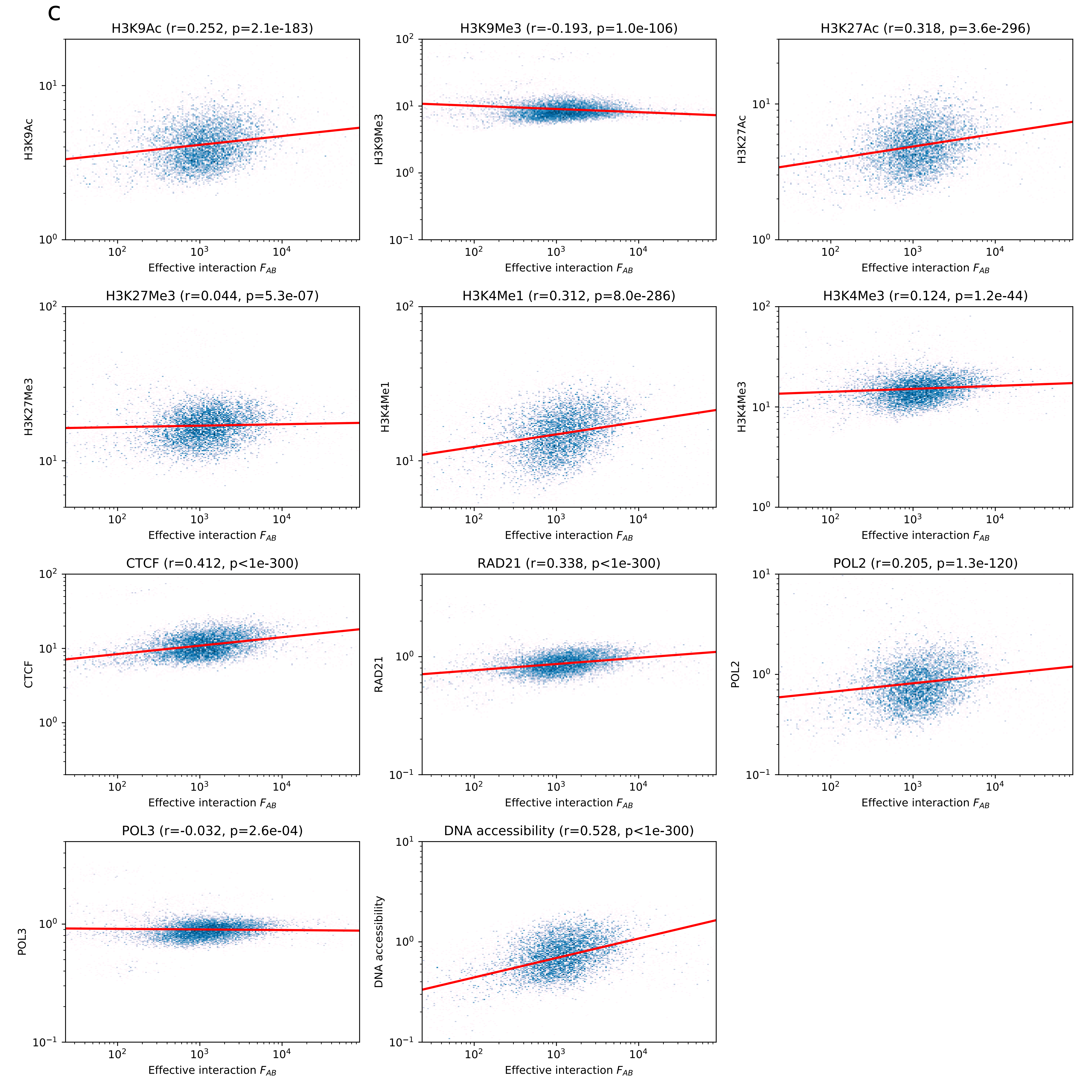
C
H3K9Ac (r=0.252, p=2.1e-183)
H3K9Me3 (r=-0.193, p=1.0e-106)
H3K27Ac (r=0.318, p=3.6e-296)
H3K27Me3 (r=0.044, p=5.3e-07)
H3K4Me1 (r=0.312, p=8.0e-286)
H3K4Me3 (r=0.124, p=1.2e-44)
CTCF (r=0.412, p<1e-300)
RAD21 (r=0.338, p<1e-300)
POL2 (r=0.205, p=1.3e-120)
POL3 (r=-0.032, p=2.6e-04)
DNA accessibility (r=0.528, p<1e-300)
Effective interaction F_{AB}
H3K9Ac
H3K9Me3
H3K27Ac
H3K27Me3
H3K4Me1
H3K4Me3
CTCF
RAD21
POL2
POL3
DNA accessibility
