# Supplementary material for: Exploring chromatin hierarchical organization via Markov State Modelling
Source: PLoS Comput Biol. 2018 Dec 31;14(12):e1006686. doi: 10.1371/journal.pcbi.1006686 (PMC6355033; doi:10.1371/journal.pcbi.1006686)
Supplement: S1 Table — These were the values used for the GM12878_primary dataset by Rao et al. [23]. (DOCX) [file pcbi.1006686.s017.docx]

| Chromosome | Thermal  annealing $\beta$ | Number of hubs $n$ | | |
| --- | --- | --- | --- | --- |
|  |  | First level | Second level | Third level |
| 1 | 9 | 8 | 16 | 38 |
| 2 | 7 | 5 | 15 | 34 |
| 3 | 7 | 5 | 10 | 29 |
| 4 | 8 | 8 | 17 | 27 |
| 5 | 7 | 6 | 16 | 38 |
| 6 | 8 | 3 | 14 | 41 |
| 7 | 7 | 4 | 10 | 34 |
| 8 | 9 | 6 | 13 | 31 |
| 9 | 8 | 5 | 12 | 35 |
| 10 | 8 | 4 | 14 | 23 |
| 11 | 9 | 6 | 15 | 26 |
| 12 | 7 | 5 | 14 | 28 |
| 13 | 9 | 6 | 14 | 24 |
| 14 | 9 | 4 | 11 | 30 |
| 15 | 9 | 5 | 15 | 28 |
| 16 | 9 | 5 | 14 | 27 |
| 17 | 9 | 5 | 12 | 27 |
| 18 | 8 | 4 | 9 | 19 |
| 19 | 8 | 5 | 10 | 25 |
| 20 | 9 | 4 | 10 | 21 |
| 21 | 8 | 5 | 13 | 25 |
| 22 | 9 | 7 | 16 | 24 |
| X | 7 | 5 | 18 | 39 |
