# Supplementary material for: Exploring chromatin hierarchical organization via Markov State Modelling
Source: PLoS Comput Biol. 2018 Dec 31;14(12):e1006686. doi: 10.1371/journal.pcbi.1006686 (PMC6355033; doi:10.1371/journal.pcbi.1006686)
Supplement: S3 Table — The color of cells denotes the effective interaction strength: red cells are scaffold-layer interactions, while orange cells are Layer 1 interactions. (PDF) [file pcbi.1006686.s019.pdf]

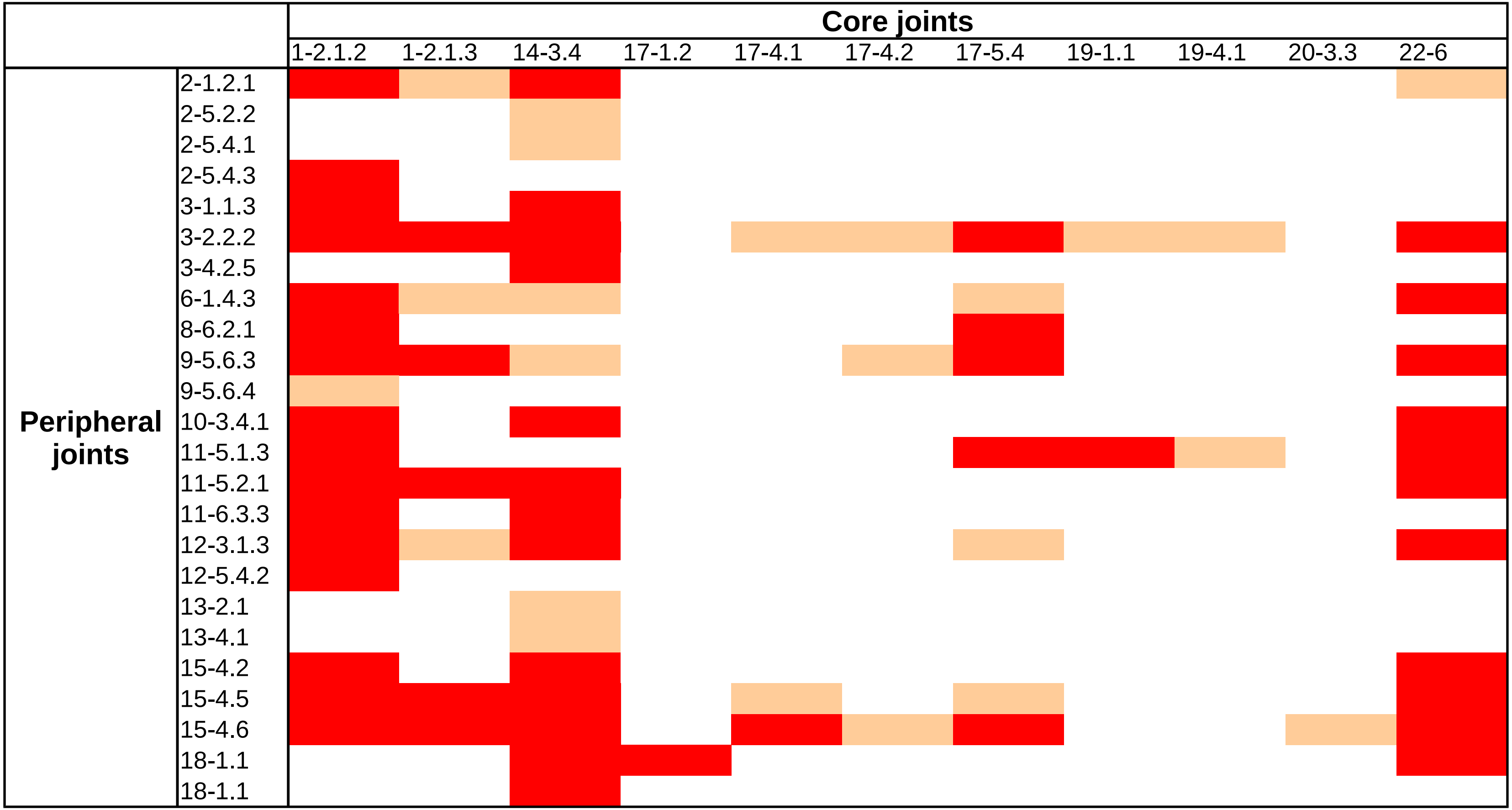
Core joints
1-2.1.2
1-2.1.3
14-3.4
17-1.2
17-4.1
17-4.2
17-5.4
19-1.1
19-4.1
20-3.3
22-6
Peripheral joints
2-1.2.1
2-5.2.2
2-5.4.1
2-5.4.3
3-1.1.3
3-2.2.2
3-4.2.5
6-1.4.3
8-6.2.1
9-5.6.3
9-5.6.4
10-3.4.1
11-5.1.3
11-5.2.1
11-6.3.3
12-3.1.3
12-5.4.2
13-2.1
13-4.1
15-4.2
15-4.5
15-4.6
18-1.1
18-1.1
