# Supplementary material for: Exploring chromatin hierarchical organization via Markov State Modelling
Source: PLoS Comput Biol. 2018 Dec 31;14(12):e1006686. doi: 10.1371/journal.pcbi.1006686 (PMC6355033; doi:10.1371/journal.pcbi.1006686)
Supplement: S6 Table — Hi-C interaction matrices were obtained by Rao et al. [23] (GEO accession GSE63525). (DOCX) [file pcbi.1006686.s022.docx]

|  | GM12878 primary | GM12878  replicate | IMR90 | HUVEC | HMEC |
| --- | --- | --- | --- | --- | --- |
| Partitioning NMI with GM12878 primary | 1.0 | 0.93 | 0.86 | 0.88 | 0.88 |
| Number of partitions | 538 | 618 | 630 | 669 | 624 |
| Average partition size (Mbp) | 5.4 | 4.7 | 4.6 | 4.3 | 4.6 |
| Number of chromosomes in largest cluster | 18 | 19 | 16 | 11 | -  (All chromosomes disconnected) |
| Chromosomes in largest cluster | 1, 2, 3, 6, 8, 9, 10 ,11 ,12, 13, 14, 15, 16, 17, 18, 19, 20, 22 | 1, 2, 3, 4, 6, 7, 9, 10 ,11 ,12, 14, 15, 16, 17, 18, 19, 20, 21, 22 | 1, 3, 6, 7, 9, 10, 11, 12, 14 ,15, 16, 17, 19, 20, 21, 22 | 1, 3, 7, 9, 10, 11, 12, 15, 16, 17, 19 | - |
| Number of partitions in significant inter-chromosomal interactions | 53 | 64 | 37 | 19 | 0 |
